# From the *p*-Factor to Cognitive Content: Detection and Discrimination of Psychopathologies Based on Explainable Artificial Intelligence

**DOI:** 10.1155/da/9943590

**Published:** 2025-05-19

**Authors:** Erkan Eyrikaya, İhsan Dağ

**Affiliations:** ^1^Department of Clinical Psychology, Graduate School of Social Science, Ankara University, Ankara, Türkiye; ^2^Department of Clinical Psychology, Faculty of Letters, Hacettepe University, Ankara, Türkiye

**Keywords:** BERT, cognitive content, I-Talk, linguistic marker, machine learning, NLP, *p*-factor, psychopathology, XAI

## Abstract

**Background and Aims:** Differentiating psychopathologies is challenging due to shared underlying mechanisms, such as the *p*-factor. Nevertheless, recent methodological advances suggest that distinct linguistic markers can help detect and differentiate these conditions. This study aimed to use cognitive content analysis with advanced natural language processing (NLP) and machine learning (ML) to (Study 1) distinguish among control, depression, anxiety, and depressive-anxiety groups and (Study 2) detect general psychopathology.

**Methods:** Data from 1901 participants (retained from 2551 respondents aged 18–43 years who completed the Beier sentence completion test [BSCT]) were analyzed. For Study 1, groups were formed using the Depression, Anxiety, and Stress Scale (DASS-21); negative mood was assessed via the Positive and Negative Affect Schedule (PANAS). For Study 2, the Brief Symptom Inventory (BSI) categorized general psychopathology and self-reported diagnostic status served as external validation. Two analytic approaches were employed: (1) textual analysis with a bidirectional encoder representations from transformers (BERT) model and (2) subscale-score analysis using a support vector machine (SVM). SHapley Additive exPlanations (SHAP) interpreted the ML models.

**Results:** In Study 1, the models distinguished control, depression, anxiety, and depressive-anxiety groups. Anxiety was marked by positive content, hope, and I-Talk, whereas depression involved negative, hopeless content. Depressive-anxiety combined features of anxiety with a pronounced negative outlook, suggesting a transitional phase where diminishing hope may bridge anxiety to depression. In Study 2, the models performed high in distinguishing the self-reported pathology diagnosis group (area under the curve [AUC]: 0.81 [BERT], 0.85 [SVM]) from subclinical samples but failed to differentiate the self-reported past diagnosis (AUC: 0.53 [BERT], 0.57 [SVM]) group from controls. This implies that cognitive changes in psychopathology may share a consistent underlying structure like *p*-factor.

**Conclusion:** These pioneer findings demonstrate that integrating advanced computational techniques can identify key linguistic markers and guide the development of language-based diagnostic tools, potentially transforming mental health diagnostics.

## 1. Introduction

The challenges in diagnosing and differentiating psychopathologies in the field of mental health constitute a significant issue both in clinical practice and within the theoretical framework. The causes of these difficulties vary, ranging from the use of heterogeneous classification systems in diagnostic processes to the challenges of conducting biopsychosocial assessments [1, 2]. However, the root cause can be traced back to the *p*-factor, a theoretical construct analogous to the intelligence *g*-factor, which represents a general psychopathology factor underlying various mental disorders [3]. The *p*-factor reflects a shared vulnerability across different forms of psychopathology, meaning that individuals with high levels of this factor are more likely to experience a broad spectrum of psychological difficulties [4].

Research suggests that the *p*-factor is associated with various neurobiological, cognitive, and environmental risk factors [5], including impaired executive functioning [6], childhood trauma [7], and genetic predispositions [8]. This general structure, which can be represented even by a single score [9], complicates the differentiation between specific disorders, as it captures the common variance across multiple mental health conditions. Nevertheless, it can be said that every psychopathology can have a characteristic thought content. This hypothesis, termed the cognitive content hypothesis, suggests that psychopathologies can be differentiated based on their specific cognitive contents [10]. Although the cognitive content hypothesis has critical value in both understanding the *p*-factor and distinguishing psychopathologies, research conducted to date has made limited progress [11].

With the aim of revealing this value, Study 1 focuses on one of the most challenging problems in psychology—differentiating depression and anxiety—based on natural language processing (NLP) and explainable artificial intelligence (XAI). In doing so, the study considered the limitations of prior research.

Previous research has often relied on linguistic inquiry and word count (LIWC) categories analyzed through classical statistics or machine learning (ML) [12–14]. However, evidence suggests that LIWC is more restrictive than direct language analysis [15]. Additionally, these studies frequently used social media data, which can be unreliable due to inaccurate self-presentation, bot influence, and cultural biases [16, 17]. To overcome these limitations, this study employed direct language analysis and collected data using the Beier sentence completion test (BSCT), a psychological analytic instrument.

Furthermore, some studies have suggested that “I-Talk” might be an indicator of negative affectivity or general distress [18, 19]. This because negative affectivity may increase self-focused attention, leading to greater I-Talk usage in the depression group; nevertheless, findings in this regard have been inconsistent and this factor has not been thoroughly examined in existing research. The effect of negative affect on I-Talk was assessed via the positive and negative affect schedule (PANAS) in this study.

Study 2, which aims to detect general psychopathology, serves two purposes: (i) to determine whether I-talk reflects general distress and (ii) to address the comorbidity problem in existing research. High comorbidity between depression and anxiety is common, as well as with other diagnoses [20, 21]. For this reason, it may be suggested that the linguistic indicators identified for depression in previous studies—except for three studies [12, 14, 22]—may actually reflect a broader spectrum of psychopathology, including anxiety. Therefore, Study 2 aims, on the one hand, to overcome the limitations of previous studies and provide a more comprehensive understanding of general distress and its linguistic markers, and on the other hand, to establish whether the *p*-factor can also be observed in cognitive content.

Finally, in addition to the NLP analyses, a second analysis was conducted using support vector machine (SVM). The SVM models were trained using the total word count, the counts of I-Talk, anxiety words, and depression words from the LIWC categories, and the subdimension scores of the BSCT assessed by the judges. This approach enhanced the reliability and validity of the findings by incorporating both linguistic and psychological assessments.

## 2. Methods

### 2.1. Participants and Procedure

The study sample comprised 2551 individuals aged 18 years and older, drawn from the general population in Türkiye. The sample consisted of 69% female and 31% male participants, with a mean age of 28.73 years (standard deviation (SD) = 9.4; range = 18–67 years).

Eligibility criteria included: (1) being between 18 and 43 years old, (2) having at least a university student status, and (3) excluding participants who provided more than two blank or nonsensical responses in the completion tasks. After applying these criteria, the final sample consisted of 1901 participants.

Participants were recruited between September 14, 2022, and March 14, 2023, via the online survey platform SurveyMonkey. The survey link was disseminated using snowball and convenience sampling methods through social media platforms (e.g., Twitter, WhatsApp) and university networks. Participation was voluntary, and no incentives were provided. Only individuals who electronically consented to participate were granted access to the online questionnaire. Ethical approval for this study was obtained from Hacettepe University Ethics Committee on September 14, 2022 (Approval No: E-12908312-300-00002390477).

### 2.2. Group Formation

Both Study 1 and Study 2 were tested using two different analytical methods. However, since the requirements of each method varied, this led to differences in the study samples depending on the chosen analysis approach. This section provides details on the process of constructing the groups used in the analyses. From the application of exclusion criteria to the creation of groups, the flow diagram of the whole process is given in Supporting Information Figure S1.

#### 2.2.1. Step 1: Normalization of Word Count

This study employed two distinct analytical approaches: (1) direct language analysis independent of human judgment using BERT and (2) an analysis based on BSCT subdimensions using SVM. The choice of analytical method influenced both the group formation process and the study sample, as word count was considered a potential confounding factor.

To address this, normalization procedures were applied according to the specific requirements of each method. To maintain consistency in text length across participants, the BERT-based analysis was normalized by the number of tokens, while the SVM-based analysis was normalized by the number of words. These adjustments ensured that linguistic variations were attributed to psychological or cognitive factors rather than differences in text length.

Following normalization, the initial sample included 1690 participants for BERT (SVM: *N* = 1684). To minimize potential biases related to mental health conditions, participants with a history of or current psychopathological disorder were excluded, resulting in a final sample of 1202 participants (SVM: *N* = 1196).

#### 2.2.2. Step 2: Preparing External Validation Groups

Previous research has typically excluded participants who self-reported psychological disorders. However, in this study, we utilized this information as an external validation set rather than an exclusion criterion. The rationale behind this approach was to enhance the robustness of our findings while offering a novel methodological contribution.

To achieve this, we have created separate self-reported groups from people who stated that they are psychopathology in the past or now. This decision was based on the premise that individuals can reliably report whether they have received a psychopathological diagnosis. Unfortunately, we did not have a chance to verify their statements. For this reason, self-reported diagnostic groups could not be used for Study 1, which required specific diagnostic groups, but we used them to create external validation groups for Study 2 (see, section Creating External Validation Tests).

#### 2.2.3. Step 3: Group Creating for Study 1

The aim of Study 1 was to differentiate between the control, depression, anxiety, and depressive-anxiety groups. To achieve this, participants in the final sample (BERT: *N* = 1202, SVM: *N* = 1196) were categorized based on their scores on the Depression, Anxiety, and Stress Scale (DASS-21). Using the scale's cutoff points, they were classified into four groups: Control (Control_G), Depression (Depression_G), Anxiety (Anxiety_G), and Depressive-Anxiety (Dep_Anx_G).

##### 2.2.3.1. Sample Description in Study 1

The Control_G demonstrated a balanced composition, with the majority being female participants (BERT: 68%, SVM: 67%) and a mean age of 27.2 years across both models. Most participants were undergraduate (84%) in both models. The Depression_G exhibited a slightly higher female representation (BERT: 65%, SVM: 70%), with a mean age of ~26.6 years. Educational levels were consistent across models, with 86% being undergraduate in BERT and 86% in SVM. In the Anxiety_G, females accounted for a significant majority (BERT: 83%, SVM: 75%), and participants were slightly younger, with mean ages of 23.89 (BERT) and 23.91 (SVM). Education levels remained stable, with 84% undergraduate in both models. The Dep_Anx_G showed the highest proportion of female participants (BERT: 76%, SVM: 70%) and mean ages of 24.74 (BERT) and 27.74 (SVM). Education levels were consistent, with the majority being undergraduate (BERT: 88%, SVM: 89%).

Overall, the demographic patterns remain broadly consistent across both models (Table 1), but the groups show slight variations in gender distribution, age, and education levels. Since the cross-validation (CV) method was be used and external validation datasets were not created, we proceeded directly to the analysis process without making group comparisons for Study 2.

#### 2.2.4. Step 4: Group Creating for Study 2

Study 2 aims to demonstrate that certain linguistic markers traditionally attributed solely to depression in the literature may, in fact, belong to a broader category of psychopathology, at least including anxiety. To achieve this, the dataset from Study 1 (BERT *N* = 1202, SVM *N* = 1196) was re-segmented using the Brief Symptom Inventory (BSI) instead of the DASS-21, forming the basis of Study 2.

To create a general psychopathology group, Global Severity Index (GSI) derived from the BSI was utilized. Participants were classified into two groups: control group (GSI < 1.0) and a subclinical group (Subclinical_G: Control_G: GSI < 1.0).

Given the sufficient sample size for binary classification, we constructed a randomly selected 20% hold-out test set for both BERT and SVM analyses. These test sets were separated from the initial data sets (BERT: *N* = 1202, SVM: *N* = 1196) before model training commenced, ensuring an external validation dataset.

##### 2.2.4.1. Creating External Validation Tests

Firstly, the consistency between BSI scores and self-reported psychopathology was examined. Specifically, for individuals who reported having a past diagnosis of psychopathology (Self_Past_Dia_G), we ensured that their GSI was below 1. Conversely, for those who reported experiencing current psychopathology (Self_Pat_Dia_G), we required their GSI to be greater than 1. Based on these criteria, the following datasets were constructed (see Supporting Information Figures S2 for BERT and S3 for SVM):

• HO_Cont_vs_Self_Pat_Dia_Test: A dataset combining the hold-out test control group (HO_Cont_G) and the current self-reported psychopathology group (Self_Pat_Dia_G).
• HO_Cont_vs_Self_Past_Dia_Test: A dataset combining the hold-out test control group (HO_Cont_G) and the past self-reported psychopathology group (Self_Past_Dia_G).
• Self_Past_vs_Pat_Dia_Test: A dataset consisting of both past (Self_Past_Dia_G) and current (Self_Pat_Dia_G) self-reported psychopathology groups.

Despite the presence of potential confounding factors^1^—such as the timing of diagnosis—we believe that the findings of Study 2 offer valuable contributions to the field, particularly in improving methodological approaches for psychopathology classification.

##### 2.2.4.2. Sample Description in Study 2

The demographic characteristics of the initial baseline datasets (BERT and SVM) are presented in Supporting Information Tables S1 and S2. For the BERT analysis (*N* = 1202, SVM *N* = 1196), the sample was predominantly female (BERT: 72%, SVM: 72%), with a mean age of 25.1 years (SD = 5.48). Similarly, for the SVM analysis, the mean age was 25.91 years (SD = 6.19).

After separating the holdout data from the initial data set, the final training data set is obtained. Demographic characteristics of the training dataset, hold-out and external validation tests are given in Table 2.

In the hold-out test set (BERT: *n* = 241, SVM: *n* = 240), the proportion of female participants remained high (BERT: 71%, SVM: 70%), with an overall mean age of 25.89 years (SD = 5.76) for BERT and 28.1 years (SD = 6.27) for SVM.

The demographic characteristics of the classification datasets varied across groups. In the HO_Cont_vs_Self_Pat_Dia_Test dataset, which included 241 participants for BERT and 240 for SVM, the proportion of female participants was 75% in both models. The mean age for BERT was 26.74 years (SD = 6.6), while for SVM, it was 26.33 years (SD = 6.4).

Similarly, in the HO_Cont_vs_Self_Past_Dia_Test dataset, which consisted of 194 participants for BERT and 193 for SVM, the proportion of female participants was 70% in both models. The mean age for BERT in this dataset was 26.5 years (SD = 6.56), whereas for SVM, it was slightly higher at 27.66 years (SD = 6.93).

Lastly, in the Self_Past_vs_Pat_Dia_Test dataset, which included 231 participants for BERT and 229 for SVM, the proportion of female participants was 70% for BERT and 79% for SVM. The mean age in this dataset was 26.39 years (SD = 6.31) for BERT and 27.45 years (SD = 6.89) for SVM.

### 2.3. Group Comparison

The potential confounding effects of demographic variables, emotional state, and total word count on the findings were considered. To address this, each group was compared both internally and with the training set in terms of gender, age, education level, income level, word count, PANAS scores.

Given the multiple comparisons conducted, *p*-values were adjusted using the Benjamini–Hochberg correction [23, 24]. The analyses revealed that none of the test sets significantly differed from the training set in terms of demographic characteristics (Supporting Information Table S15). However, for both studies, the HO_Cont_vs_Self_Past_Dia_Test set showed significant differences in emotional state compared to the training set. This discrepancy was expected, as both groups in this test set exhibited highly similar levels of negative and positive affect, whereas the training set included participants from a subclinical psychopathology group, leading to a difference in affective scores.

Before conducting comparisons within the test sets, the minimum expected cell frequency assumption of the chi-square test was verified [25]. Since at least 80% of the cells were required to have frequencies greater than five, the doctoral and master's degree categories were merged for analysis. The results indicated that only the BERT HO_Cont_vs_Self_Pat_Dia_Test set showed a significant gender effect. However, Cramér's *V* (0.196) indicated a small effect size. In contrast, no significant differences were found in other test sets where the control group and self-reported psychopathology groups were included.

Furthermore, the effect of demographic variables on word count was examined across all subsets. To ensure statistical validity, normality and homogeneity of variance assumptions were assessed. Given that Shapiro–Wilk tests indicated non-normal distributions [26], Mann–Whitney *U* tests were used for comparisons. The results revealed no significant differences in word count across test sets based on demographic variables (Supporting Information Table S16). Given these findings, and considering the small effect size of gender differences in the HO_Cont_vs_Self_Pat_Dia_Test set, no corrective actions were deemed necessary.

#### 2.3.1. Assessing the Impact of Demographic Similarities on Model Performance

To rule out the possibility that performance differences in external validation were due to demographic or other test-set characteristics, comparisons were conducted between Self_Past_vs_Pat_Dia_Test and the hold-out test set across gender, education level, SES, age, word count, PANAS negative, and PANAS positive scores. No significant differences were detected (Supporting Information Table S17), strengthening the assumption that the test sets were demographically comparable.

#### 2.3.2. Comparing Key Groups to Interpret Model Performance Variability

Since the test sets did not significantly differ demographically, direct comparisons of self-reported psychopathology groups and subclinical groups were conducted to gain insights into model performance variability. For this purpose, the following group comparisons were performed: (1) Self_Past_Dia_G vs. HO_Cont_G, (2) HO_SubC_G vs. Self_Pat_Dia_G.

These groups were compared in terms of I-Talk frequency, depression, anxiety, and BSI scores (Supporting Information Table S18 and Supporting Infomation Figure S19).

The results revealed significant differences between Self_Past_Dia_G and HO_Cont_G in terms of depression-related word count (BERT: *U* = 3617.5, *p*  < 0.05, *r* = 0.20) and anxiety-related word count (BERT: *U* = 3505, *p*  < 0.05, *r* = 0.22). Additionally, a significant difference in I-Talk frequency was found between HO_SubC_G and Self_Pat_Dia_G (BERT: *U* = 7762.5, *p*  < 0.05, *r* = 0.17; SVM: *U* = 7385.5, *p*  < 0.05, *r* = 0.19). In the SVM group, a significant difference also emerged between HO_SubC_G and Self_Pat_Dia_G in terms of anxiety word count (*U* = 7683.5, *p*  < 0.05, *r* = 0.17).

Overall, the multiple comparisons indicated that while demographic variables were evenly distributed across groups, certain linguistic features significantly differed. However, these linguistic differences were inconsistent (except I-Talk) across analysis methods, and their effect sizes were small. Thus, it can be inferred that self-reported groups and subclinical groups may exhibit slightly differences in terms of traditional statistical methods, yet their impact on model performance remains limited.

Conclusion, for Study 1, a subset of 1202 participants was derived from the original dataset of 2551 individuals collected from the general population. For Study 2, an expanded dataset of 1505 participants were constructed, including external validation sets. To ensure comparability across test conditions, the control group remained constant within all external validation sets.

The conclusions drawn from these comparisons are based on 100 distinct models tested 100 times for each group, utilizing diverse datasets generated through CV. The use of consistent group structures in external test sets enhances comparability and reinforces the robustness of the findings. Flow diagrams for all group formation processes are given in Supporting Information Figure S1.

### 2.4. Measures

#### 2.4.1. The Beier Sentence Completion Test

The BSCT is a semi-structured projective tool developed by the American psychologist Delton C. Beier to assess individuals' general attitudes, tendencies, and desires [27]. Unlike other sentence completion tests, the BSCT is distinguished by its extensive set of 67 prompts, each specifically designed to evaluate 13 distinct subdimensions of life [27]. Higher negative scores show higher levels of problems in the corresponding area ([28], for scoring process, see Supporting Information).

##### 2.4.1.1. Why was BSCT Preferred for Data Collection?

Language is a fundamental means through which individuals perceive and interpret their experiences, shaping the meanings they attribute to them [29]. Research indicates that self-referential texts are particularly well-suited for identifying linguistic markers, as they provide direct insights into an individual's cognitive and emotional processes [30]. Given that the BSCT is a structured written assessment designed to capture cognitive content related to subjective experiences across 13 life subdomains, responses to this test are expected to reflect psychopathological cognitive patterns.

Empirical studies support this assumption, demonstrating that BSCT subscales are influenced by underlying psychopathological conditions and are sensitive to clinical interventions [28, 31–33]. Therefore, considering its capacity to reveal psychopathological cognition, the BSCT was deemed a more appropriate tool for linguistic analysis.

Moreover, utilizing the BSCT strengthens the study by enabling a dual-approach analysis. Through BERT, cognitive content can be analyzed independently of human interpretation, leveraging NLP techniques. Concurrently, SVM analyses incorporate human input and digitization, allowing for nuanced interpretation. By integrating these two methods and considering the results as complementary, the study enhances its reliability while increasing the explanatory power and comprehensiveness of its findings.

#### 2.4.2. Depression, Anxiety, and Stress Scale

The DASS developed by Lovibond and Lovibond [34] consists of 42 items in total. The scale used in this thesis is DASS-21, which is the short form of DASS-42. The Turkish adaptation of the scale was conducted by Sarıçam [35]. The adaptation study was conducted with data collected from both psychiatric patients and two different subject groups without any psychological problems. According to the results of the exploratory factor analysis, the 21-item scale consisted of three sub-dimensions (depression, anxiety and stress). When confirmatory factor analysis was performed, it was found that the three sub-dimensional structure had excellent fit index and fit index values acceptable in clinical and normal sampling. Each sub-dimension of the scale, which is evaluated on a four-point Likert-type rating, consists of seven items. In this study, Cronbach's alpha coefficients were calculated as 0.88 for depression subscale, 0.87 for stress subscale and 0.84 for anxiety subscale. DASS-21 categorizes these psychopathologies into severity levels using five distinct cutoff points.

#### 2.4.3. Brief Symptom Inventory

The BSI was developed by Derogotis [36] to screen for general psychopathology. The validity and reliability study of the scale in Türkiye was conducted by Şahin and Durak [37]. The validity and reliability study was carried out with four separate studies in different sample groups. Cronbach's alpha internal consistency coefficients obtained for the whole BSI ranged between 0.93 and 0.96. The inventory, which consists of 53 items in total, is Likert-type and the items are scored between 0 and 4. As a result of the test, three global indices can be obtained. These are respectively Gloval Severity Index, Positive Symptom Total Index and Positive Symptom Discomfort Index. GSI, one of the global indices, evaluates whether psychiatric symptoms are at pathological level. The GSI, which is calculated by dividing the total score obtained from the scale by the total number of items (53), being above 1.0 indicates the presence of psychopathological symptoms, and being below 1.0 indicates that the symptoms are not on the psychopathological border.

#### 2.4.4. Positive and Negative Affect Schedule

The PANAS was developed by Watson et al. [38] to examine a person's emotional state. The scale consists of a total of 20 items, 10 of which measure negative emotional state, while the other 10 items assess positive emotional state. The evaluation of the scale is based on a five-point Likert-type rating. Turkish adaptation of the scale was made by Gençöz [39]. The internal consistency coefficient of the whole scale was 0.86 for the positive emotion subscale and 0.83 for the negative emotion subscale.

#### 2.4.5. Linguistic Inquiry and Word Count

LIWC software is a word counting program that provides information about the language of the written text it analyses in about 80 linguistic categories. These categories include pronouns (I, you, etc.), emotion words (happy, sad, etc.), cognitive words (thinking, attention, etc.), conjunctions (but, but, etc.), movement words (go, come, etc.), and anxiety words (anxiety, worry, etc.) [40]. The adaptation study into Turkish was conducted by Müderrisoğlu [41]. LIWC dictionary was used to calculate I-Talk_O_Count, I-Talk_G_Count, Depression Word Count, and Anxiety Word Count required for SVM analyses.

I-Talk, characterized by the use of first-person singular pronouns (e.g., ‘I,' ‘me,' ‘my'). In Turkish, ‘I' can be expressed explicitly with the pronoun ‘ben' (e.g., Ben depresyondayım [I am depressed]) or implicitly through grammatical structures like verb conjugations and possessive suffixes (e.g., Depresyondayım [I am depressed]). Accordingly, SVM analyses were performed in two formats: one for explicit 'I' statements (I_Talk_O_Count) and another encompassing both implicit and explicit forms (I_Talk_G_Count).

We have collected the necessary information through the demographic form in the collection of demographic data and the formation of external validation groups.

### 2.5. Model Training

The objective of Study 1 was to develop a four-class classification model to distinguish between the control group, anxiety group, depression group, and depressive-anxiety group. In contrast, the goal of Study 2 was to classify individuals into a general psychopathology group.

To achieve these objectives, written responses from the BSCT were utilized as input for model training. This approach allowed for the analysis of cognitive content through two distinct methods: direct linguistic analysis and human-judged subdimension scores.

For the linguistic analysis, BERT models were trained, whereas for BSCT subdimension score-based analysis, SVM models were employed. Throughout the entire training and testing process, CV was implemented to ensure robust performance estimation.

#### 2.5.1. Cross-Validation

CV is a critical technique in ML used for assessing model generalizability and robustness. In this approach, CV, the dataset is partitioned into a predefined number of folds [42]. For example, if the number of folds is chosen as 10, the entire dataset is split into 10 folds; 9 folds are used for model training, and the remaining 1 fold serves as the validation set. This process is repeated 10 times, ensuring that each fold is used as a test set once. Consequently, CV results in as many model trainings as the number of folds.

From a psychological research perspective, CV can be seen as a methodological tool for ensuring the replicability of findings across different datasets. Given the ongoing replication crisis in psychology, some researchers argue that CV should be considered a gold standard for psychological research [43–45]. By systematically validating models on different subsets of data, CV enhances the reliability of findings and strengthens the generalizability of psychological findings.

Beyond its role in replication, CV is also essential for mitigating overfitting—a phenomenon where a model memorizes patterns in the training data but fails to generalize to unseen data [46]. By implementing CV, researchers can obtain a more reliable estimate of model performance and reduce the risk of overfitting [47, 48]. However, this repeated training process comes at a computational cost, which becomes even more demanding when using repeated CV. In this variant, the fold-splitting process is randomly repeated multiple times to enhance reliability.

Another advanced form of CV is nested CV, which consists of an inner loop for model selection (finding optimal model parameters) and an outer loop for model evaluation. This approach minimizes bias and provides an unbiased estimate of the true error rate [49]. By separating model selection from performance evaluation, nested CV is regarded as one of the most rigorous validation techniques in ML, offering a more accurate assessment of a model's true generalizability.

Parameter optimization is an approach that systematically tests various hyperparameters to identify the configuration that maximizes model performance. However, this process increases computational costs. Due to the high computational demands of BERT analyses, which require Graphics Processing Unit (GPU)-based processing, we conducted an optimal parameter search only for SVM models. This resulted in differences in CV process.

**Figure 1:**
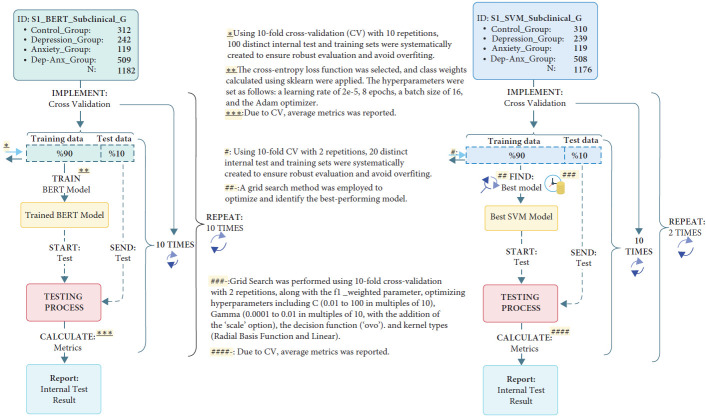
Flow diagram of the BERT and SVM training processes for Study 1. Symbols (*∗*, *∗∗*, *∗∗∗*) indicate steps in the BERT training process (cross-validation, training configuration, and performance reporting), while (#, ##, ###, ####) correspond to SVM steps: cross-validation, model selection, hyperparameter tuning, and performance reporting.

**Figure 2:**
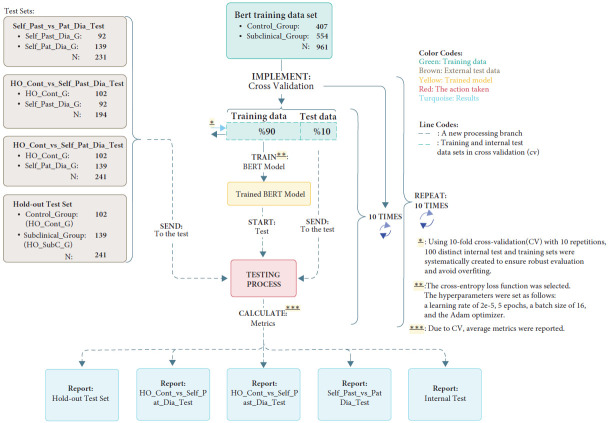
Flow diagram of the BERT training process for Study 2. Symbols (*∗*, *∗∗*, *∗∗∗*) indicate steps in the BERT training process, including cross-validation, training configuration, and performance reporting. (The flow diagram for SVM is provided in Supporting Information Figure S9).

In both studies, BERT analyses were conducted using 10-repeated 10-fold CV. However, for SVM analyses, Study 1 employed 2-repeated 10-fold CV, while Study 2 used 1-repeated 10-fold CV. A flowchart detailing the entire model training process is provided in Figures 1 and 2.

#### 2.5.2. Transfer Learning

Transfer learning is a ML approach that leverages the knowledge acquired by a pre-trained model and applies it to a new problem, rather than training a model from scratch [50]. This method enables the model to update existing relationships or learn new ones based on the target dataset, rather than learning all relationships from scratch.

In language-based tasks, transfer learning is predominantly implemented using large pretrained models, such as BERT [51]. The process begins with pretraining a large language model on extensive text data, where the model learns generalizable linguistic features from unlabeled corpora [52]. Subsequently, fine-tuning is performed on a smaller labeled dataset, enabling the model to adapt to specific tasks. During fine-tuning, certain model parameters are adjusted, allowing for high performance even with limited data and shorter training times [53].

The amount of data required for effective fine-tuning depends on the specific task, dataset characteristics, and applied techniques. However, due to its ability to enhance performance, reduce training time, and minimize data requirements, transfer learning is considered a game-changing advancement in NLP applications [54].

##### 2.5.2.1. BERT Model

There are many pretrained language models based on transfer learning (e.g., Electra, DistilBERT). However, this study employs BERT as the primary model for text analysis, leveraging its efficiency, scalability, and superior performance in NLP tasks. Indeed, the specific reason for choosing BERT in this study is that the Turkish BERT model possesses a richer vocabulary compared to other available alternatives. This characteristic enhances its ability to capture linguistic nuances more effectively in Turkish text analysis, making it the most suitable choice for the task at hand.

Additionally, since pretrained models better capture linguistic structures, they often outperform traditional lexicon-based approaches, such as LIWC. For example, in a study comparing LIWC and fine-tuned BERT models for detecting anxiety-related language, BERT demonstrated superior performance [15]. Therefore, it was important to choose the BERT model to be comparable with similar studies in the literature (e.g [55]).

#### 2.5.3. BERT Training

Despite its numerous advantages, BERT has a significant drawback—its GPU-dependent training process makes it computationally expensive and time-consuming. Given these constraints, this study fine-tuned BERT without hyperparameter optimization, instead relying on commonly used default values in the field to balance computational cost and efficiency.

For fine-tuning, the BERT model was first loaded using the Hugging Face library. Once the model was configured, training was initiated using CV. The fine-tuning process followed a 10-fold, 10-repetition CV approach, which involved the following steps:

(1) A stratified sub-dataset (inner-test set) was randomly extracted, comprising 10% of the total dataset. (2) The model was trained on the remaining 90% of the data and evaluated on the 10% test set. (3) The process was repeated with a different 10% subset while using the remaining data for training. (4) Since the study employed a 10-repetition, 10-fold CV framework, this process was repeated 100 times, and the average performance metrics were reported. A flowchart summarizing the training parameters and fine-tuning process is provided in Figures 1 and 2.

#### 2.5.4. Support Vector Machine Models

For the analysis utilizing BSCT subtest scores, the SVM method was chosen. This decision was based on several key advantages of SVM over alternative approaches. SVM is highly interpretable and explainable, making it well-suited for psychological research. Additionally, it performs well on small datasets and offers robust generalization capabilities. Compared to deep learning models, SVM is computationally more efficient while also providing flexible solutions to common challenges such as class imbalances.

One of the primary strengths of SVM is that it can yield substantial performance improvements over traditional statistical methods. However, like other classical ML techniques, SVM analysis requires extensive data preprocessing. Before applying the model, raw data must be transformed into an appropriate format for analysis, and potential issues such as multicollinearity must be addressed.

##### 2.5.4.1. Data Preprocessing

In this section, the preliminary processing for SVM analysis was mentioned.

*2.5.4.1.1. Step 1: Data Preparation*. The most time-consuming and resource-intensive phase of this study was data preparation for SVM analysis. The dataset consisted of 2551 sentence completion responses, all of which required manual scoring. Given the high cost and time demand of studies requiring human raters, an optimized approach was implemented to enhance efficiency: one referee scored the whole test, while the other two referees scored 20% of the test items in common with each other and with the first referee.

To reduce the burden on the primary rater and increase both scoring efficiency and reliability, a sentence grouping technique was used. Specifically, responses were clustered within each question based on 100% text similarity. This strategy improved evaluation efficiency while ensuring that the reliability assessment accurately reflected true agreement levels.

Without this approach, randomly selecting 20% of responses could have led to clusters of highly similar answers, potentially inflating reliability estimates. By employing structured grouping, this methodological issue was effectively mitigated, ensuring a more valid and representative assessment of inter-rater reliability.

*2.5.4.1.2. Step 2: Scoring*. To facilitate the scoring process for raters, a BSCT scoring tool was developed using the C# programming language. The raters were provided with BSCT sentence responses uploaded into the program, along with a detailed scoring guideline, and the scoring process was initiated (Supporting Information Figure S16).

The scoring was conducted by three expert raters experienced in the use of this test. To ensure inter-rater reliability, the Gwet AC1 test was employed, a reliability metric that has gained prominence in recent years, particularly in peer-reviewed studies in healthcare research [56–58]. The reliability calculation indicated that the BSCT sentence scores ranged between moderate (0.40) and excellent (1.0) inter-rater agreement, and only those meeting this reliability criterion were included in the analysis (Supporting Information Table S13).

*2.5.4.1.3. Step 3: Multicollinearity Analysis*. Before conducting SVM analysis, it was necessary to assess multicollinearity, a potential issue when predictor variables are highly correlated. Two widely used approaches for detecting multicollinearity were applied: (1) correlation analysis among features and (2) variance inflation factor (VIF) analysis.

It is recommended that VIF values should be below 10, and tolerance values should not be below 0.20 for the included variables [59, 60]. Within the scope of the analyses conducted in this context, it was observed that the VIF values of none of the variables exceeded 5 and the tolerance values were not below 0.25 (Supporting Information Table S14), while the correlation matrix showed that positive attitudes were moderately and highly correlated with negative attitudes. Despite this, no variable exhibited a correlation of 0.90 or above, confirming the absence of severe multicollinearity (Supporting Information Figure S18).

The final selection of variables for the SVM analysis was guided by the research objectives, dataset size, computational efficiency, and the interpretability of the resulting model. To optimize both financial and time-related costs, the following variables were selected: BSCT negative scores, total word count (Word_Count), and the number of word for depression, anxiety, and self-referential words derived from the LIWC categories.

#### 2.5.5. Support Vector Machine Training

Due to its cost-effectiveness, parameter optimization for this model was conducted using the GridSearch method, which is widely used in the field. The purpose of this optimization was to explore the optimal SVM parameters that would yield the best results. The parameter values tested in this process were selected based on commonly used values in existing literature. A flowchart detailing the model training process is provided in Figure 1 (for Study 2, see Supporting Information Figure S9).

In conclusion, the combined use of these two methods for analyzing cognitive content is expected to strengthen the findings. Additionally, the inclusion of both primary training sets and datasets varied through CV is believed to enhance the validity and generalizability of the results.

### 2.6. Model Explanation

As ML models become increasingly complex, understanding how they make decisions has become more challenging. This issue is particularly critical in fields such as healthcare and psychology, where the reliability and interpretability of model decisions are essential. XAI encompasses a range of techniques designed to make the decision-making processes of models more transparent. By utilizing XAI, it is possible to determine which inputs the model prioritizes and how these factors influence its decisions, thereby increasing transparency [61].

Ultimately, the role of XAI in this study was to reveal the features that influenced the decisions of the trained models. For BERT models, these features corresponded to words, while for SVM models, they corresponded to BSCT subdimension scores. This not only allowed for an examination of how model outputs align with psychological theory and clinical assessments but also presented an opportunity to advance theoretical and clinical interpretations of psychopathology.

#### 2.6.1. SHapley Additive exPlanations

SHAP, which is based on game theory, quantifies the contribution of each input feature to the model's output [62]. This approach allows for an objective assessment of the factors influencing model predictions, providing deeper insights into the decision-making process.

In this study, model interpretability was achieved using the SHAP library, which is widely regarded as the gold standard for explainability in the field. SHAP provides two primary types of explanations [63]: global explanations and local explanations.

Global explanations analyze the overall decision-making mechanism of the model, identifying the most influential variables across all predictions. These explanations help in understanding how the model behaves on the entire dataset and which features contribute most significantly to its predictions. Local explanations, on the other hand, focus on individual predictions, providing insights into why the model arrived at a particular decision for a specific input. This approach enhances transparency by allowing a more detailed examination of model outputs at the instance level.

In this study, both types of explanations were utilized to comprehensively interpret model decisions. For this purpose, after completing the model training process, each model was reprocessed sequentially according to its CV fold, and SHAP values were computed using the test data from each CV iteration. For SVM models, the “KernelExplainer” was applied, while for BERT models, the “PartitionExplainer” was used. Given that CV was implemented throughout both training and testing phases, aggregated SHAP values were computed to obtain a more stable and generalizable interpretability assessment.

### 2.7. Model Evaluation

This section outlines the evaluation metrics used to assess model performance. In Study 1, area under the curve (AUC) values based on the one-versus-rest (OvR) approach and the confusion matrix are presented to provide a comprehensive assessment of classification performance. In Study 2, which involves a binary classification task, both standard AUC values and overall performance metrics are reported.

#### 2.7.1. Receiver Operating Characteristic Curve

In this study, the receiver operating characteristic (ROC) curve and the AUC metric were employed to evaluate the performance of the trained models. AUC-ROC is one of the fundamental metrics for assessing the discriminative ability of a classification model [64, 65]. The ROC curve illustrates the trade-off between the true-positive rate (TPR) and the false-positive rate (FPR) across different classification thresholds. The AUC value, representing the area under the ROC curve, serves as a comprehensive measure of the model's classification performance.

#### 2.7.2. One-Versus-Rest

AUC-ROC analysis is primarily used for binary classification, but it can also be extended to multi-class classification problems. A widely adopted approach for this purpose is the OVR method, which transforms a multi-class classification problem into multiple binary classification tasks [66].

In the OVR approach, each class is treated as the positive class, while all other classes are collectively assigned as negative. This process is repeated for each class, effectively creating *N* separate binary classification problems for an *N*-class dataset. For each of these binary classifiers, a separate ROC curve is plotted, and an AUC score is computed, indicating how well the specific class is distinguished from the others. AUC values for each class, followed by the calculation of macro and micro-averaged AUC scores to represent overall performance.

In conclusion, in Study 1, AUC-ROC values were calculated separately for each class using the OVR approach and then visualized to facilitate interpretation. Additionally, confusion matrices were employed to evaluate model performance by analyzing classification errors. This dual evaluation approach allowed for an examination of which classes were predicted more accurately and which exhibited higher misclassification rates in Study 1.

## 3. Results

### 3.1. Study 1: Distinguishing Control Group, Depression, Anxiety, and Depressive-Anxiety

**Figure 3:**
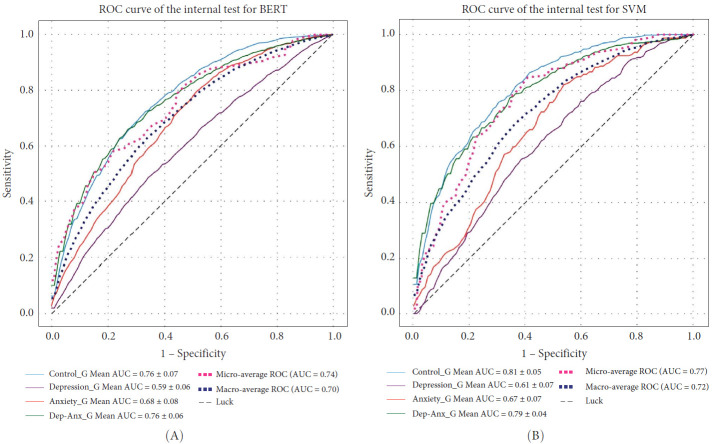
The ROC curves illustrate the predictive performance of the models in a one-vs-rest classification task, with (A) depicting the results for BERT and (B) for SVM. Macro-average AUC values (BERT: 0.70, SVM: 0.72) emphasize the modelsʼ overall capability to discriminate across all classes equally, while micro-average AUC values (BERT: 0.74, SVM: 0.77) highlight performance weighted by sample prevalence. Notably, both models excel in distinguishing the ‘Control_G' class (BERT: 0.76, SVM: 0.81), yet face greater difficulty with the ‘Depression_G' class (BERT: 0.59, SVM: 0.61). Overall, the results affirm that both models significantly outperform random classification (diagonal line).

For the control group, BERT achieved an AUC of 0.76 and SVM achieved 0.81; for the depression group, BERT achieved 0.59 and SVM achieved 0.61; for the anxiety group, BERT achieved 0.68 and SVM achieved 0.67; and for the depressive-anxiety group, BERT achieved 0.76 and SVM achieved 0.79. The question arises whether this level of performance is sufficient (Figure 3).

To the best of our knowledge, this is the first study to use a large language model and the sentence completion test (SCT) in this way for four-group classification. For this reason, direct comparisons with previous studies are not possible. However, the models have demonstrated reasonable performance, particularly when considering the inherent challenges of distinguishing closely related psychopathological categories.

Closer examination of the confusion matrix (Supporting Information Figure S13) provides further insight into the models' performance. Both models showed difficulty in differentiating between the anxiety group and the control group, with misclassification rates of 28% for BERT and 37% for SVM. Similarly, distinguishing between the depression group and the depressive-anxiety group posed challenges, with misclassification rates of 39% for BERT and 27% for SVM. Consequently, while attempting to distinguish among four groups, the models provided insights into the cognitive content of all three disorders.

SHAP analyses revealed significant differences in cognitive content between depression and anxiety, with depressive-anxiety appearing as an intersection of the two. Notably, depressive-anxiety incorporates nearly all markers of depression (Supporting Information Tables S4 and S5), underscoring its hybrid nature and the complexity of its classification.

**Figure 4:**
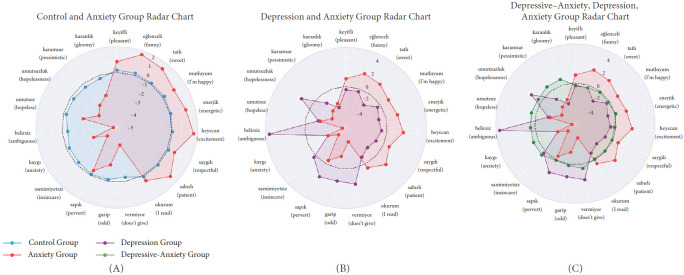
The words contributing most to classification within the relevant class are the aggregated SHAP values obtained by explaining all models using the SHAP library (see, Supporting Information Tables S4 and S5). The top five words contributing most to inclusion in each class are displayed in the radar chart above, with (A) comparing the Control Group and the Anxiety Group, (B) comparing the Anxiety Group and the Depression Group, and (C) comparing the Depressive–Anxiety, Depression, and Anxiety Groups. The black dashed line represents the zero-circle: words located within this circle contribute negatively to class inclusion, whereas words outside the circle contribute positively. For instance, the word energetic does not support inclusion in the depression class but positively contributes to inclusion in the depressive-anxiety and anxiety classes.

**Figure 5:**
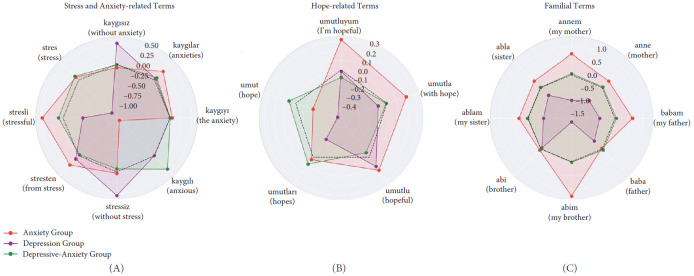
Radar charts illustrating thematic differences in linguistic content among Depression, Anxiety, and Depressive–Anxiety groups, according to the SHAP values obtained by explaining the BERT model in Study 1. (A) Focuses on stress and anxiety-related terms, showing that, unlike Depression, these terms are more strongly associated with Anxiety and Depressive–Anxiety groups. (B) Examines hope-related terms, revealing lower hope expressions in the Depression and Depressive–Anxiety groups compared to the Anxiety group. (C) Visualizes familial terms, with the Anxiety group demonstrating stronger associations with family-related expressions. These findings highlight distinct cognitive content patterns across psychopathology groups.

Furthermore, the SHAP values of the BERT model revealed several key findings (Figures 4 and 5):

(i) Cognitive content is significantly more positive in anxiety compared to depression. (ii) The depression group exhibits prominent peaks in the “without anxiety” and “without stress” dimensions, indicating cognitive content characterized by a lack of anxiety and stress. In contrast, the anxiety group shows higher values in dimensions such as “stressful” and “anxieties” reflecting a greater focus on general experiences of anxiety. The depressive-anxiety group displays a balanced profile across stress and anxiety terms, suggesting cognitive content similar to that of the anxiety group. (iii) Words related to hope and family effectively discriminate between anxiety and depression, while the depressive-anxiety group largely maintains a balanced profile. (iv) Although classical statistical analysis does not indicate a significant difference in terms of I-Talk2, it is observed that implicit I-Talk—used as a hidden subject in direct cognitions—is more dominant in anxiety and depressive-anxiety (also see Supporting Information Figure S5).

**Figure 6:**
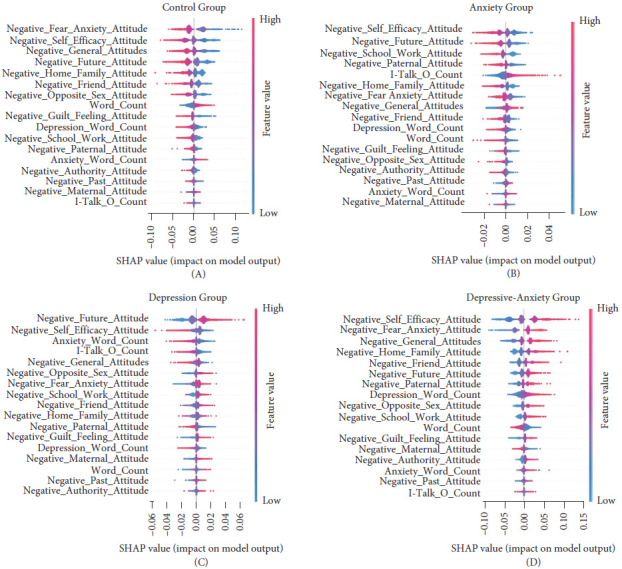
SHAP model explanations can be categorized into two types: local and global. Global importance summarizes feature relevance across all classes, while local importance provides insights specific to individual classes. The graphs above represent the locally aggregated SHAP values for the SVM models trained in Study 1 (for global importance, see Supporting Information Figure S10), illustrating the impact of features on the probability of belonging to the indicated class—(A) for the Control Group, (B) for the Anxiety Group, (C) for the Depression Group, and (D) for the Depressive–Anxiety Group. Each point corresponds to a data instance, with red dots indicating high feature values and blue dots indicating low values. The right side of the y-axis shows the probability of belonging to the specified class, while the left side indicates the probability of belonging to any other class. For instance, increasing I-Talk values contributes to the likelihood of being in the Anxiety and Depressive–Anxiety groups, whereas decreasing values are more predictive of the Depression and Control groups. In addition, local explanations of depression and anxiety groups related to I-Talk_G_Count are given in Supporting Information Figure S14.

Consistent results were found using the SVM model (Figure 6). Specifically, an increased use of I-Talk and anxiety words was associated with anxiety, while increased negative attitudes toward family and the future were associated with depression (SHAP Graph for I_Talk_G_Count, see Supporting Information Figure S14). The depressive-anxiety group, on the other hand, appears to have a distribution reflecting a combination of depression and anxiety characteristics.

#### 3.1.1. Negative Emotion Assessment

PANAS scores were compared between groups to assess negative affectivity (Supporting Information Section PAS1). The anxiety group reported slightly lower negative affect than the depression group (BERT: $\overset{-}{\text{X}}$ = 22.53; SVM: $\overset{-}{\text{X}}$ = 22.45 vs. BERT: $\overset{-}{\text{X}}$ = 23.65, *U* = 15997, *p*  > 0.05; SVM: $\overset{-}{\text{X}}$ = 23.70, *U* = 16002.5, *p*  > 0.05). However, the control group reported lower positive affect than the anxiety group (BERT: $\overset{-}{\text{X}}$ = 31.88; SVM: $\overset{-}{\text{X}}$ = 31.91 vs. BERT: $\overset{-}{\text{X}}$ = 33.30, *U* = 16430.5, *p*  > 0.05; SVM: $\overset{-}{\text{X}}$ = 33.33, *t* = −1.948, *p*  > 0.05). As a result, the difference in the above I-Talk did not associate with negative emotion (Supporting Information Section PAS1).

### 3.2. Study 2: Detection of General Psychopathology

The models demonstrated robust performance on both the internal dataset (BERT: 0.78 AUC; SVM: 0.78 AUC) and the hold-out dataset (BERT: 0.78 AUC; SVM: 0.79 AUC). However, the models revealed their best performance on the HO_Cont_vs_Self_Pat_Dia_Test, with AUC scores of 0.81 for BERT and 0.85 for SVM. Conversely, when comparing Self_Past_vs_Pat_Dia_Test, the models achieved slightly higher AUC scores (BERT: 0.80; SVM: 0.82) than on the hold-out test set. In contrast, the models could not differentiate between the HO_Cont_vs_Self_Past_Dia_Test, achieving AUC scores of 0.53 for BERT and 0.57 for SVM (see Supporting Information Figure S12 for ROC curve; Supporting Information Tables S10 and S11 for overall performance metrics).

**Figure 7:**
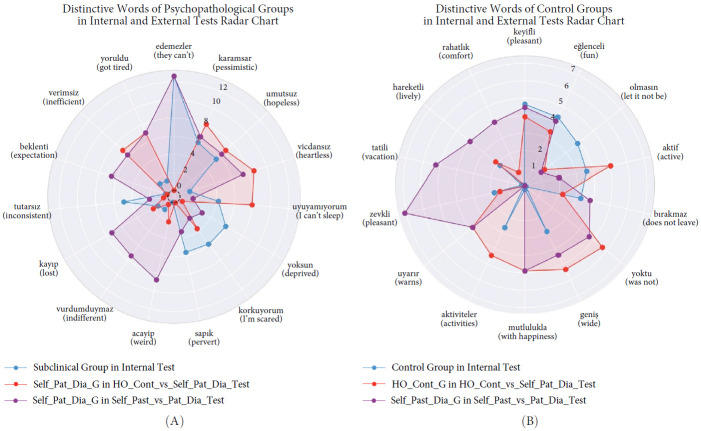
The top predictive words for each group, derived from the SHAP explanations obtained for Study 2 are visualized. (A) Radar chart compares the Self_Pat_Dia_G from two test sets—HO_Cont_vs_Self_Pat_Dia_Test (red) and Self_Past_vs_Pat_Dia_Test (purple)—with the Subclinical Group in the Internal Test Set (blue). (B) Radar chart focuses on Control Groups, comparing the HO_Cont_G from the HO_Cont_vs_Self_Pat_Dia_Test (red) and the Self_Past_Dia_G from Self_Past_vs_Pat_Dia_Test (purple) with the Control Group in the Internal Test Set (blue). Detailed word lists and their attributions can be found in Supporting Information Tables S6 and S7.

Since classical statistical analyses showed no differences in BSI scores^3^ between groups, what accounts for this differentiation among the test sets? Given that the external validation sets consisted of combinations of the same groups, this consistent performance difference can be attributed to cognitive content varying with the severity of psychopathology. For example, the SHAP values for the phrase “I can't sleep” are lowest in the internal test, highest in the HO_Cont_vs_Self_Pat_Dia_Test, and intermediate in the Self_Past_vs_Pat_Dia_Test (Figure 7). Therefore, the SHAP values related to word usage appear to be directly connected to the severity of psychopathology. To our knowledge, no prior studies have investigated this phenomenon, making direct comparisons impossible. However, this finding aligns with both the differentiation of I-Talk, depression word count, and anxiety word count in self-reported psychopathology groups and the explanations provided by the SVM model (Supporting Information Figure S11).

## 4. Discussion

### 4.1. General Psychopathology

Study 2 was designed to detect general psychopathology and aimed to demonstrate that linguistic markers previously identified for depression may, in fact, reflect a broader spectrum of psychopathology, including anxiety. Additionally, it sought to gain a deeper understanding of the concept of I-Talk. However, the fact that the models performed better in the HO_Cont_vs_Self_Pat_Dia_Test despite being trained on a subclinical group added a new dimension to the study's findings. Specifically, SHAP values increased when transitioning from the subclinical group in the internal test set to the Self_Pat_Dia_G and decreased when moving to the Self_Past_Dia_G. This finding suggests that the cognitive changes occurring during the development and treatment of psychopathology may have a common and consistent structure. Through this structure, BSCT responses, which reflect personal and unique experiences, enabled a distinction between the control and the Self_Pat_Dia_G while also revealing that the Self_Past_Dia_G became indistinguishable from the control group. These results may provide crucial insights into the process of psychopathology from onset to remission. For instance, examining the relationship between relapse and words that appear with low frequency in the Self_Past_Dia_G but with high frequency in other groups may be a valuable avenue for clinical research.

These findings are consistent with previous research demonstrating that cognitive changes begin long before a formal diagnosis is made, as highlighted in early diagnosis studies [67]. Furthermore, linguistic markers have been shown to vary with the severity of psychopathology [68, 69], and cognitive (linguistic) changes occur during or after treatment [68, 70]. In line with this, the changes in cognitive content observed in this study further support the view that the *p*-factor is a transdiagnostic structure related to cognitive processes [9]. These results provide significant contributions to understanding the underlying cognitive mechanisms of psychopathology and developing more effective treatment strategies.

An essential component of this transdiagnostic structure appears to be self-focused attention (I-Talk). Although the use of an SCT instead of free writing was expected to limit I-Talk, its differentiation between groups was notable. Moreover, its emergence as a crucial feature in the model's decisions highlights its strong effect. From this perspective, future studies exploring the connection between the *p*-factor and I-Talk could significantly enhance our understanding of psychopathology.

### 4.2. Depressive-Anxiety

Despite evidence supporting the *p*-factor as part of a transdiagnostic approach [71], it remains unclear whether the cognitive content hypothesis can effectively differentiate between psychopathologies. This study suggests that this uncertainty can be elucidated through XAI. The analysis reveals that anxiety differs from depression primarily in terms of positive cognitive content, anxiety-related words, family attitude, hope, and I-Talk. In contrast, depression is predominantly characterized by entirely negative and hopeless cognitive content. These defining features of depression complicate the differentiation between depression and depressive-anxiety. However, while depressive-anxiety and anxiety share similarities in cognitions—ranging from anxiety words to I-Talk—depressive-anxiety is uniquely marked by more negative cognitive content and diminished hope. Notably, prior studies indicate that anxiety often precedes depression in individuals diagnosed with depressive-anxiety, with the loss of hope and negative cognitive styles playing critical roles in its development [72, 73].

Additionally, previous research suggests that hope serves as a mediating variable in reducing anxiety symptoms, with treatment effects on hope significantly contributing to symptom improvement [74]. Consistent with these findings, this study demonstrates that the balanced distribution of depressive-anxiety between depression and anxiety, combined with the fluctuating pattern of hope-related words, indicates a continuous transition from anxiety to depressive-anxiety. As a result, the decrease in hope and the reduction of positive cognitive content emerges as two key factors contributing to the development of depressive-anxiety.

Furthermore, the observation that depressive-anxiety primarily originates from anxiety is supported by more than just these two factors identified through the BERT analysis. For instance, when comparing the SHAP graph of the SVM model for depressive-anxiety with the other, certain features such as an increase in anxiety word count and I-Talk, as well as a decrease in negative maternal attitude and word count, align with patterns observed in anxiety. In contrast, a significant portion of the remaining features corresponds to those associated with depression. Another example is that while attitudes toward the future are negative in depression, they tend to be positive in anxiety; yet, attitudes toward the past are negative in both conditions. Considering that hope is fundamentally a future-oriented construct, it can be argued that the emergence of depressive-anxiety is linked to the development of a negative outlook on the future as anxiety-driven coping mechanisms fail [75, 76]. This progression supports the notion that individuals with anxiety increasingly tend to experience depression over time.

To conclusion, this XAI-based study clearly demonstrates that implementing strategies aimed at increasing or enhancing hope in preventive interventions and therapy could potentially prevent the formation of depressive-anxiety groups. However, the crucial point is that although common mechanism manifests in cognitive content, the cognitive content differ sufficiently to allow for an acceptable degree of differentiation between the two closely related disorders. As a result, the key finding of this pioneering study is that longitudinal studies based on XAI and NLP may lead to the development of linguistic diagnostic and statistical manual of mental disorders (DSMs), potentially revolutionizing diagnostic criteria through language analysis.

### 4.3. Anxiety and I-Talk

The results of this study clearly demonstrate that I-Talk serves as an indicator of general psychological distress, with a specific connection to anxiety. This raises an important question: How can I-Talk function as an indicator of both anxiety and broader psychopathology? Aside from the potential connection of I-Talk with the *p*-factor, an explanation could be the anxiety buffer theory. According to the anxiety buffer theory, psychological dysfunctions are assumed to result from inadequate management of anxiety [77]. Therefore, considering the high comorbidity of anxiety disorders with other psychological conditions [20, 21], it can be argued that anxiety serves as a primary factor in the development of a wide range of psychopathologies. Consequently, I-Talk, as an indicator of anxiety, may emerge as a shared marker of various psychopathologies while being more prominent in anxiety disorders. This leads to the following conclusion: There may be a link between the *p*-factor—which is the common mechanism underlying all psychopathologies—and anxiety. This link may have made I-Talk a marker of both the p-factor and anxiety.

In contrast to previous studies comparing anxiety and depression [12, 14, 22], this study found that I-Talk clearly emerged as an indicator of anxiety. Why did this occur? There are three main reasons why this finding was more pronounced here compared to other studies: (i) The materials used in previous studies may not have been suitable for reflecting cognitive content. Unlike session transcripts with therapist interactions, or noisy social media data, SCT provide participants with the opportunity to freely express their cognitions in specific areas. In this context, the use of an analytical test may have not only improved the findings but also expanded their scope. (ii) The pursuit of psychological help usually occurs at a late stage [78, 79]. However, during this process, symptoms tend to intensify, and additional problems may arise [80]. This may be because a common mechanism causes the characteristic features of all disorders to become increasingly similar as their severity increases. This could have led to ambiguity in determining whether I-Talk is a symptom of depression or anxiety. (iii) A significant portion of previous research relied on LIWC and classical statistics, but the complexity of human nature can only be uncovered through sophisticated analyses that directly study cognition [15, 81]. For this reason, since the existence of a common mechanism requires a calculation beyond the number of words, significant differences were found in this study.

### 4.4. Limitations

Both Study 1 and Study 2 used models trained with psychometric scale-based grouped data. However, our findings were not validated in a clinically diagnosed sample. This limitation suggests that future research should further test the methodology on clinically verified individuals to assess its applicability in clinical settings.

Another limitation of Study 2 is the use of external validation datasets. The pathology groups in these datasets are based on participants' self-reported psychopathology without clinical interviews to confirm their diagnosis. Since self-reported diagnoses may be prone to biases and inaccuracies, our findings may not be directly generalizable to clinical samples. Future studies should address this limitation by comparing models trained on subclinical samples with both self-reported diagnosis groups and clinically diagnosed populations. This approach would provide a more comprehensive understanding of the applicability of AI-driven content analysis in mental health research.

Another limitation of external validation is that a significant proportion of participants did not report a diagnosis (Supporting Information Figure S15). Among those who did, the majority reported depression and anxiety-related conditions. This limits the diversity of the dataset and may restrict the generalizability of our findings to a broader spectrum of psychopathological conditions. To enhance generalizability, future studies should consider including individuals with a wider range of mental health conditions beyond anxiety and depression.

Finally, our study sample had a predominance of female participants. Although statistical comparisons revealed no significant differences in demographic variables that would affect our findings, this gender imbalance remains a limitation. Future research should aim for more gender-balanced samples to ensure the broader applicability of the results across different populations.

## 5. Conclusion

The key contribution of this research is to illustrate that although the common mechanism of psychopathologies leads to similarities in cognitive content, the analysis of cognitive content can be a critical tool for differentiating psychopathologies. Therefore, future longitudinal studies may lead to significant improvements in diagnostics and classification: (i) Tracking cognitive content can lead to a leap in diagnostics by identifying the onset and relapses of psychopathologies and the distinctive features of each. (ii) Linguistic DSMs can be created by revealing the cognitive content of each psychopathology. (iii) The formation and development of the common mechanism can be elucidated by linguistic studies on the basis of XAI.

## Figures and Tables

**Table 1:** Demographic overview of four groups (control, depression, anxiety, and depressive-anxiety groups) across two models (BERT and SVM).

	Control_G				Depression_G				Anxiety_G				Dep_Anx_G			
	BERT		SVM		BERT		SVM		BERT		SVM		BERT		SVM	
	*N*	%	*N*	%	*N*	%	*N*	%	*N*	%	*N*	%	*N*	%	*N*	%
Gender																
Women	211	68	210	66	158	65	157	66	99	83	98	82	388	76	389	77
Men	101	32	100	34	84	35	82	34	20	17	21	18	121	24	119	23
Age																
18–29	248	72	222	71	173	72	169	71	104	87	104	87	430	85	430	85
30–43	88	28	88	29	69	28	70	29	15	13	15	13	79	15	78	15
Income level																
Lower-Middle	16	5	16	7	18	7	18	7	8	7	8	7	61	12	63	12
Middle	108	35	107	38	89	37	90	38	44	37	44	37	223	44	220	43
Middle-upper	188	60	187	55	135	56	131	55	67	56	67	56	225	44	225	44
Education level																
Associate's or bachelor's degree	262	84	260	86	208	86	205	86	100	84	100	84	452	88	451	89
Master degree	39	12	39	12	30	12	30	12	14	12	14	12	49	10	49	10
Doctorate degree	11	4	11	2	4	2	4	2	5	4	5	4	8	2	8	1

**Table 2:** Demographic Overview of the Training Dataset and External Validation Groups in Study 2 Based on Two Distinct Models (BERT and SVM).

	Train data set								External validation group															
	Control group				Subclinical group				HO_Cont_G				HO_SubC_G				Self_Pat_Dia_G				Self_Past_Dia_G			
	BERT		SVM		BERT		SVM		BERT		SVM		BERT		SVM		BERT		SVM		BERT		SVM	
	*N*	%	*N*	%	*N*	%	*N*	%	*N*	%	*N*	%	*N*	%	*N*	%	*N*	%	*N*	%	*N*	%	*N*	%
Gender																								
Women	279	69	277	68	416	75	418	76	66	65	68	67	106	76	102	74	114	82	113	82	69	75	69	76
Men	128	31	129	32	138	25	132	24	36	35	34	33	33	24	36	26	25	18	25	18	23	25	22	24
Age																								
18–29	297	73	287	71	465	84	462	84	70	69	78	76	114	82	113	82	100	72	99	72	56	61	56	62
30–43	110	27	119	29	89	16	88	16	32	31	24	24	25	18	25	18	39	28	39	28	36	39	35	38
Income level																								
Lower-middle	25	6	27	7	56	10	62	11	6	5	5	5	18	13	13	47	7	5	7	54	4	4	4	6
Middle	136	34	129	32	240	43	234	43	36	35	43	42	57	41	60	43	58	42	56	41	32	35	32	39
Middle-upper	246	60	250	61	258	47	254	46	60	59	54	53	64	46	65	9	74	53	75	5	56	61	55	55
Education level																								
Associate's or bachelor's degree	350	86	343	85	483	88	487	88	84	82	90	84	124	89	115	83	125	90	124	90	71	77	71	78
Master degree	45	11	49	12	60	10	53	10	15	15	11	13	13	9	20	14	11	18	11	8	19	21	18	20
Doctorate degree	12	3	14	3	11	2	10	2	3	3	1	1	2	1	3	2	3	2	3	2	2	2	2	2

*Note*: The training data set is the sample after the hold-out data set has been separated. External validation groups are the groups used to create external validation test sets.

## Data Availability

Data for this study are not publicly available due to the sensitive nature of the language provided by participants. However, the data that support the findings of this study may be available from the corresponding author upon reasonable request.

## Endnotes

^1^Information on the time of diagnosis, whether he/she receives regular support, whether he/she takes medication or not is provided by Supporting Information Figures S20–S23.

^2^I_Talk_O_Count was significantly greater in anxiety compared to depression (For BERT, η² = .004; SVM, η² = .004), with no significant between-group differences observed for I_Talk_G_Count (*p* > 0.05) (Supporting Information Section PAS1 and Supporting Information Figure S5).

^3^There is no significant difference in terms of BSI between Subclinical Group in hold-out test set ($\overset{-}{\text{X}}$ = 1.83) and Self_Pat_Dia_G ($\overset{-}{\text{X}}$ = 1.94), nor between the HO_Cont_G ($\overset{-}{\text{X}}$ = 0.55) vs. Self_Past_Dia_G ($\overset{-}{\text{X}}$ = 0.62) (*p* >  0.05).

## Supporting Information

Supporting Information Supporting Information. Figure S1: Flow diagram outlining the steps followed to create groups. Figure S2: Process of generating external test sets for the BERT model in Study 2. Figure S3: Process of generating external test sets for the SVM model in Study 2. Figure S4: Distribution of PANAS values for groups created for the BERT model in Study 1. Figure S5: It compares the average values of variables (I-Talk Count, Depression and Anxiety Word Count, PANAS scores) across Control, Depression, Anxiety, and Dep-Anx groups in Study 1. Figure S6: Distribution of I-Talk values for groups created for the BERT model in Study 1. Figure S7: Distribution of PANAS values for groups created for the SVM model in Study 1. Figure S8: It illustrates the distribution of I-Talk scores for Control, Depression, Anxiety, and Dep-Anx groups in Study 1 for the SVM analyses. Figure S9: The flow diagram of SVM analysis in Study 2. Figure S10: Global feature importance graph for the SVM analysis in Study 1. Figure S11: Global feature importance graph for the SVM analysis in Study 2. Figure S12: AUC-ROC values for the SVM and BERT models in Study 2. Figure S13: Confusion matrix for the models generated through cross-validation for Study 1. Figure S14: Model explanation for the SVM analysis using I_Talk_G_Count in Study 1. Figure S15: Self-reported diagnoses of pathology groups from Study 2. Figure S16: Workflow illustrating the scoring process of collected data by referees and its preparation for analysis. Figure S17: BCST scoring tool designed to assist referees, including example sentences. Figure S18: Correlation matrix evaluating multicollinearity for the SVM analysis. Figure S19: It compares the average values of variables (I_Talk Count, Depression and Anxiety Word Count, and PANAS scores) across HO_Cont_G, Self_Pat_Dia_G, HO_SubC_G, and Self_Pat_Dia_G groups in Study 2. Figure S20: Bar graph depicting the timing of the last treatment for the self-past diagnosis group created for the BERT model in Study 2. Figure S21: Bar graph depicting the timing of the last treatment for the self-past diagnosis group created for the SVM model in Study 2. Figure S22: It shows the distribution of current treatment status within the self-pathology diagnosis group based on the time since diagnosis, psychological support status, and medication use in Study 2 for the BERT analyses. Figure S23: It presents the distribution of current treatment status in the pathology diagnosis group based on time since diagnosis, psychological support status, and medication use for the SVM analysis in Study 2 for the SVM analyses. Table S1: Demographic characteristics of the sample for the BERT model in Study 2. Table S2: Demographic characteristics of the sample for the SVM model in Study 2. Table S3: Words with the highest impact on model decisions in Study 1. Table S4: Words and SHAP values that have the most impact on being included in the relevant class in Study 1. Table S5: Words and SHAP values that have the most impact on not being included in the relevant class in Study 1. Table S6: Top 50 words most predictive of pathology groups in the HO_Cont_vs_Self_Pat_Dia_Test and Self_Past_vs_Pat_Dia_Test sets, as well as the subclinical group in the internal test for Study 2. Table S7: Top 50 words most predictive of control groups in the internal test, HO_Cont_vs_Self_Pat_Dia_Test, and Self_Past_vs_Pat_Dia_Test sets. Table S8: Average model performance metrics for BERT and SVM in Study 1. Table S9: Comparison of SVM analysis results when the variable I_Talk_O_Count was replaced with I_Talk_G_Count in Study 1. Table S10: Average model performance metric for BERT and SVM in Study 2. Table S11: Comparison of SVM analysis results when the variable I_Talk_O_Count was replaced with I_Talk_G_Count in Study 2. Table S12: Comparison of SVM analysis results (AUC) when the variable I_Talk_O_Count was replaced with I_Talk_G_Count in Study 2. Table S13: GWET AC1 values calculated based on referee scoring. Table S14: VIF values for SVM models. Table S15: Multiple comparisons confirming that the external validation test sets in Study 2 share similar demographic characteristics, word count, and emotional content with the training set. Table S16: Internal comparisons of the external validation test sets created for Study 2. Table S17: Multiple comparisons examining differences between self-reported pathology diagnosis groups, past diagnosis groups, and the hold-out test set in Study 2. Table S18: Comparisons between the self-past diagnostic group and the hold-out control group, as well as between the hold-out subclinical pathology group and the psychopathology diagnostic group.

## References

[1] Chen Z. S., Kulkarni P. P., Galatzer-Levy I. R., Bigio B., Nasca C., Zhang Y. (2022). Modern Views of Machine Learning for Precision Psychiatry. *Patterns*.

[2] Merten E. C., Cwik J. C., Margraf J., Schneider S. (2017). Overdiagnosis of Mental Disorders in Children and Adolescents (in Developed Countries). *Child and Adolescent Psychiatry and Mental Health*.

[3] Caspi A., Houts R. M., Belsky D. W. (2014). The p Factor: One General Psychopathology Factor in the Structure of Psychiatric Disorders?. *Clinical Psychological Science*.

[4] Caspi A., Moffitt T. E. (2018). All for One and One for All: Mental Disorders in One Dimension. *American Journal of Psychiatry*.

[5] Lynch S. J., Sunderland M., Newton N. C., Chapman C. (2021). A Systematic Review of Transdiagnostic Risk and Protective Factors for General and Specific Psychopathology in Young People. *Clinical Psychology Review*.

[6] Snyder H. R., Friedman N. P., Hankin B. L. (2019). Transdiagnostic Mechanisms of Psychopathology in Youth: Executive Functions, Dependent Stress, and Rumination. *Cognitive Therapy and Research*.

[7] McLaughlin K. A., Colich N. L., Rodman A. M., Weissman D. G. (2020). Mechanisms Linking Childhood Trauma Exposure and Psychopathology: A Transdiagnostic Model of Risk and Resilience. *BMC Medicine*.

[8] Lahey B. B., Krueger R. F., Rathouz P. J., Waldman I. D., Zald D. H. (2017). A Hierarchical Causal Taxonomy of Psychopathology Across the Life Span. *Psychological Bulletin*.

[9] Adam D. (2023). P Factor” Could Open an Important Window on Core Attributes of Mental Health Maladies. *Proceedings of the National Academy of Sciences of the United States of America*.

[10] Clark D. A., Beck A. T., Brown G. (1989). Cognitive Mediation in General Psychiatric Outpatients: A Test of the Content-Specificity Hypothesis. *Journal of Personality and Social Psychology*.

[11] Beck R., Perkins T. S. (2001). Cognitive Content-Specificity for Anxiety and Depression: A Meta-Analysis. *Cognitive Therapy and Research*.

[12] Stade E. C., Ungar L., Eichstaedt J. C., Sherman G., Ruscio A. M. (2023). Depression and Anxiety Have Distinct and Overlapping Language Patterns: Results From a Clinical Interview. *Journal of Psychopathology and Clinical Science*.

[13] Rai S., Stade E. C., Giorgi S. (2024). Key Language Markers of Depression on Social Media Depend on Race. *Proceedings of the National Academy of Sciences of the United States of America*.

[14] Sonnenschein A. R., Hofmann S. G., Ziegelmayer T., Lutz W. (2018). Linguistic Analysis of Patients With Mood and Anxiety Disorders During Cognitive Behavioral Therapy. *Cognitive Behaviour Therapy*.

[15] Teferra B. G., Rose J. (2023). Predicting Generalized Anxiety Disorder From Impromptu Speech Transcripts Using Context-Aware Transformer-Based Neural Networks: Model Evaluation Study. *JMIR Mental Health*.

[16] Schlosser A. E. (2020). Self-Disclosure Versus Self-Presentation on Social Media. *Current Opinion in Psychology*.

[17] Ramirez-Esparza N., Chung C., Kacewic E., Pennebaker J. (2008). The Psychology of Word Use in Depression Forums in English and in Spanish: Testing Two Text Analytic Approaches. *Proceedings of the International AAAI Conference on Web and Social Media*.

[18] Tackman A. M., Sbarra D. A., Carey A. L. (2019). Depression, Negative Emotionality, and Self-Referential Language: A Multi-Lab, Multi-Measure, and Multi-Language-Task Research Synthesis. *Journal of Personality and Social Psychology*.

[19] Mor N., Winquist J. (2002). Self-Focused Attention and Negative Affect: A Meta-Analysis. *Psychological Bulletin*.

[20] Murphy J. M., Horton N. J., Laird N. M., Monson R. R., Sobol A. M., Leighton A. H. (2004). Anxiety and Depression: A 40-Year Perspective on Relationships Regarding Prevalence, Distribution, and Comorbidity. *Acta Psychiatrica Scandinavica*.

[21] Koyuncu A., İnce E., Ertekin E., Tükel R. (2019). Comorbidity in Social Anxiety Disorder: Diagnostic and Therapeutic Challenges. *Drugs in Context*.

[22] Brockmeyer T., Zimmermann J., Kulessa D. (2015). Me, Myself, and I: Self-Referent Word Use as an Indicator of Self-Focused Attention in Relation to Depression and Anxiety. *Frontiers in Psychology*.

[23] Jafari M., Ansari-Pour N. (2019). Why, When and How to Adjust Your P Values?. *Cell Journal*.

[24] Benjamini Y., Hochberg Y. (1995). Controlling the False Discovery Rate: A Practical and Powerful Approach to Multiple Testing. *Journal of the Royal Statistical Society Series B: Statistical Methodology*.

[25] McHugh M. L. (2013). The Chi-Square Test of Independence. *Biochemia Medica*.

[26] Razali M. N., Wah Y. B. (2011). Power Comparisons of Shapiro-Wilk, Kolmogorov-Smirnov, Lilliefors and Anderson-Darling Tests. *Journal of Statistical Modeling and Analytics*.

[27] Akkoyun F. (2014). Projektif Teknikler.

[28] Koç G., Çolak B., Tatlı S. Z., İlhan R. S., Oncu B. (2021). Beier Sentence Completion Test Profiles of Adolescents and Emerging Adults With Internalizing and Externalizing Disorders. *Adolescent Psychiatry*.

[29] Chung C., Pennebaker W., Fiedler K. (2007). The Psychological Functions of Function Words. *Social Communication*.

[30] Havigerová J. M., Haviger J., Kučera D., Hoffmannová P. (2019). Text-Based Detection of the Risk of Depression. *Frontiers in Psychology*.

[31] Kaya M. S. (2022). The Use of Dynamic Cognitive Behavioural Therapy (DCBT) in Social Anxiety Disorder (SAD): A Theoretical Integration Initiative. *Medicina (Kaunas, Lithuania)*.

[32] Herdi O., Akaslan D. S., Ertan E. A., Alptekin G., Şentürk Cankorur V. (2019). Dinamik Yönelimli Etkileşim Grup Psikoterapisinde Iyileşmenin Beier Cümle Tamamlama Testi Ile Değerlendirilmesi. *Kriz Dergisi*.

[33] Yıldırım O. (2022). The Effect of Dynamic Oriented Brief-Intensive-Emergency Psychological Counseling on Depression. *Mehmet Akif Ersoy Üniversitesi Eğitim Fakültesi Dergisi*.

[34] Lovibond P. F., Lovibond S. H. (1995). The Structure of Negative Emotional States: Comparison of the Depression Anxiety Stress Scales (DASS) With the Beck Depression and Anxiety Inventories. *Behaviour Research and Therapy*.

[35] Sarıçam H. (2018). The Psychometric Properties of Turkish Version of Depression Anxiety Stress Scale-21 (DASS-21) in Community and Clinical Samples. *Journal of Cognitive-Behavioral Psychotherapy and Research*.

[36] Derogatis L. R. (1992). *The Brief Symptom Inventory (BSI): Administration, Scoring & Procedures Manual-II*.

[37] Sahin N. H., Durak A. (1994). Kisa Semptom Envanteri (Brief Symptom Invetory-BSI): Turk Gencleri Icin Uyarlanmasi. *Türk Psikoloji Dergisi*.

[38] Watson D., Clark L. A., Tellegen A. (1988). Development and Validation of Brief Measures of Positive and Negative Affect: The PANAS Scales. *Journal of Personality and Social Psychology*.

[39] Gençöz T. (2000). Positive and Negative Affect Schedule: A Study of Validity and Reliability. *Türk Psikoloji Yazıları*.

[40] Pennebaker J., Booth R., Boyd R., Francis M. (2015). *Linguistic Inquiry Andword Count: LIWC*.

[41] Müderrisoğlu S. Türkçe Psikolojik Metin Analizi Programı: LIWC Türkçe [Turkish psychological Text Analysis Program: Turkish LIWC] [Poster].

[42] Song Q. C., Tang C., Wee S. (2021). Making Sense of Model Generalizability: A Tutorial on Cross-Validation in R and Shiny. *Advances in Methods and Practices in Psychological Science*.

[43] Bokhari E., Hubert L. (2018). The Lack of Cross-Validation Can Lead to Inflated Results and Spurious Conclusions: A Re-Analysis of the MacArthur Violence Risk Assessment Study. *Journal of Classification*.

[44] Koul A., Becchio C., Cavallo A. (2018). Cross-Validation Approaches for Replicability in Psychology. *Frontiers in Psychology*.

[45] de Rooij M., Weeda W. (2020). Cross-Validation: A Method Every Psychologist Should Know. *Advances in Methods and Practices in Psychological Science*.

[46] Ng A. Y. (1997). Preventing “Overfitting” of Cross-Validation Data. *ICML*.

[47] Berrar D. (2016). Cross-Validation. *Reference Collection in Life Sciences*.

[48] Stone M. (1974). Cross-Validatory Choice and Assessment of Statistical Predictions. *Journal of the Royal Statistical Society Series B: Statistical Methodology*.

[49] Varma S., Simon R. (2006). Bias in Error Estimation When Using Cross-Validation for Model Selection. *BMC Bioinformatics*.

[50] Torrey L., Shavlik J., Olivas E. Soria (2010). Transfer Learning. *Handbook of Research on Machine Learning Applications and Trends: Algorithms, Methods, and Techniques*.

[51] Rogers A., Kovaleva O., Rumshisky A. (2020). A Primer in BERTology: What We Know About How BERT Works. *Transactions of the Association for Computational Linguistics*.

[52] Qasim R., Bangyal W. H., Alqarni M. A., Ali Almazroi A. (2022). A Fine-Tuned BERT-Based Transfer Learning Approach for Text Classification. *Journal of Healthcare Engineering*.

[53] Wang Z. (2023). A New Computationally Efficient Method to Tune BERT Networks—Transfer Learning. *Journal of Physics: Conference Series*.

[54] Chakkarwar V., Tamane S., Thombre A. (2023). A Review on BERT and Its Implementation in Various NLP Tasks. *Proceedings of the International Conference on Applications of Machine Intelligence and Data Analytics (ICAMIDA 2022)*.

[55] Alshammari Q., Akyüz S. (2024). Mental Health on Twitter in Turkey: Sentiment Analysis With Transformers. *Decision Making in Healthcare Systems*.

[56] Gwet K. L. (2008). Computing Inter-Rater Reliability and Its Variance in the Presence of High Agreement. *British Journal of Mathematical and Statistical Psychology*.

[57] Wongpakaran N., Wongpakaran T., Wedding D., Gwet K. L. (2013). A Comparison of Cohen’s Kappa and Gwet’s AC1 When Calculating Inter-Rater Reliability Coefficients: A Study Conducted With Personality Disorder Samples. *BMC Medical Research Methodology*.

[58] Zec S., Soriani N., Comoretto R., Baldi I. (2017). High Agreement and High Prevalence: The Paradox of Cohen’s Kappa. *The Open Nursing Journal*.

[59] O’brien R. M. (2007). A Caution Regarding Rules of Thumb for Variance Inflation Factors. *Quality & Quantity*.

[60] Lin F.-J. (2008). Solving Multicollinearity in the Process of Fitting Regression Model Using the Nested Estimate Procedure. *Quality & Quantity: International Journal of Methodology*.

[61] Doshi-Velez F., Kim B. (2017). Towards A Rigorous Science of Interpretable Machine Learning. http://arxiv.org/pdf/1702.08608.

[62] Lundberg S. M., Lee S.-I. (2017). A Unified Approach to Interpreting Model Predictions. *Advances in Neural Information Processing Systems*.

[63] Mosca E., Szigeti F., Tragianni S., Gallagher D., Georg G. SHAP-Based Explanation Methods: A Review for NLP Interpretability. https://aclanthology.org/2022.coling-1.406/.

[64] Hajian-Tilaki K. (2013). Receiver Operating Characteristic (ROC) Curve Analysis for Medical Diagnostic Test Evaluation. *Caspian Journal of Internal Medicine*.

[65] Nahm F. S. (2022). Receiver Operating Characteristic Curve: Overview and Practical Use for Clinicians. *Korean Journal of Anesthesiology*.

[66] Wandishin M. S., Mullen S. J. (2009). Multiclass ROC Analysis. *Weather and Forecasting*.

[67] Eichstaedt J. C., Smith R. J., Merchant R. M. (2018). Facebook Language Predicts Depression in Medical Records. *Proceedings of the National Academy of Sciences of the United States of America*.

[68] Nook E. C., Hull T. D., Nock M. K., Somerville L. H. (2022). Linguistic Measures of Psychological Distance Track Symptom Levels and Treatment Outcomes in a Large Set of Psychotherapy Transcripts. *Proceedings of the National Academy of Sciences of the United States of America*.

[69] Muñoz S., Iglesias C.Á. (2023). Detection of the Severity Level of Depression Signs in Text Combining a Feature-Based Framework With Distributional Representations. *Applied Sciences*.

[70] Dirkse D., Hadjistavropoulos H. D., Hesser H., Barak A. (2014). Linguistic Analysis of Communication in Therapist-Assisted Internet-Delivered Cognitive Behavior Therapy for Generalized Anxiety Disorder. *Cognitive Behaviour Therapy*.

[71] Romer A. L., Hariri A. R., Strauman T. J. (2021). Regulatory Focus and the *p* Factor: Evidence for Self-Regulatory Dysfunction as a Transdiagnostic Feature of General Psychopathology. *Journal of Psychiatric Research*.

[72] Lamers F., van Oppen P., Comijs H. C. (2011). Comorbidity Patterns of Anxiety and Depressive Disorders in a Large Cohort Study. *The Journal of Clinical Psychiatry*.

[73] Starr L. R., Davila J. (2012). Responding to Anxiety With Rumination and Hopelessness: Mechanism of Anxiety-Depression Symptom Co-Occurrence?. *Cognitive Therapy and Research*.

[74] Gallagher M. W., Long L. J., Richardson A. (2020). Examining Hope as a Transdiagnostic Mechanism of Change Across Anxiety Disorders and CBT Treatment Protocols. *Behavior Therapy*.

[75] Richardson A. L. (2023). Hope and Anxiety. *Current Opinion in Psychology*.

[76] Gilbert P. (2009). Depression and Stress: A Biopsychosocial Exploration of Evolved Functions and Mechanisms. *Stress (Amsterdam, Netherlands)*.

[77] Yetzer A. M., Pyszczynski T., Routledge C., Vess M. (2019). Chapter 18—Terror Management Theory and Psychological Disorder: Ineffective Anxiety-Buffer Functioning as a Transdiagnostic Vulnerability Factor for Psychopathology. *Handbook of Terror Management Theory*.

[78] Wang P. S., Berglund P. A., Olfson M., Kessler R. C. (2004). Delays in Initial Treatment Contact After First Onset of a Mental Disorder. *Health Services Research*.

[79] Kessler R. C., Amminger G. P., Aguilar-Gaxiola S., Alonso J., Lee S., Ustün T. B. (2007). Age of Onset of Mental Disorders: A Review of Recent Literature. *Current Opinion in Psychiatry*.

[80] Thompson A., Hunt C., Issakidis C. (2004). Why Wait? Reasons for Delay and Prompts to Seek Help for Mental Health Problems in an Australian Clinical Sample. *Social Psychiatry and Psychiatric Epidemiology*.

[81] Graham S., Depp C., Lee E. E. (2019). Artificial Intelligence for Mental Health and Mental Illnesses: An Overview. *Current Psychiatry Reports*.

