# Supplementary material for: From the p-Factor to Cognitive Content: Detection and Discrimination of Psychopathologies Based on Explainable Artificial Intelligence
Source: Depress Anxiety. 2025 May 19;2025:9943590. doi: 10.1155/da/9943590 (PMC12105905; doi:10.1155/da/9943590)
Supplement: Supporting Information — Figure S1: Flow diagram outlining the steps followed to create groups. Figure S2: Process of generating external test sets for the BERT model in Study 2. Figure S3: Process of generating external test sets for the SVM model in Study 2. Figure S4: Distribution of PANAS values for groups created for the BERT model in Study 1. Figure S5: It compares the average values of variables (I-Talk Count, Depression and Anxiety Word Count, PANAS scores) across Control, Depression, Anxiety, and Dep-Anx groups in Study 1. Figure S6: Distribution of I-Talk values for groups created for the BERT model in Study 1. Figure S7: Distribution of PANAS values for groups created for the SVM model in Study 1. Figure S8: It illustrates the distribution of I-Talk scores for Control, Depression, Anxiety, and Dep-Anx groups in Study 1 for the SVM analyses. Figure S9: The flow diagram of SVM analysis in Study 2. Figure S10: Global feature importance graph for the SVM analysis in Study 1. Figure S11: Global feature importance graph for the SVM analysis in Study 2. Figure S12: AUC-ROC values for the SVM and BERT models in Study 2. Figure S13: Confusion matrix for the models generated through cross-validation for Study 1. Figure S14: Model explanation for the SVM analysis using I_Talk_G_Count in Study 1. Figure S15: Self-reported diagnoses of pathology groups from Study 2. Figure S16: Workflow illustrating the scoring process of collected data by referees and its preparation for analysis. Figure S17: BCST scoring tool designed to assist referees, including example sentences. Figure S18: Correlation matrix evaluating multicollinearity for the SVM analysis. Figure S19: It compares the average values of variables (I_Talk Count, Depression and Anxiety Word Count, and PANAS scores) across HO_Cont_G, Self_Pat_Dia_G, HO_SubC_G, and Self_Pat_Dia_G groups in Study 2. Figure S20: Bar graph depicting the timing of the last treatment for the self-past diagnosis group created for the BERT [file 9943590.f1.docx]

From the p-factor to Cognitive Content: Detection and Discrimination of Psychopathologies Based on Explainable AI

Erkan Eyrikaya^1a^^[[1]](#footnote-1)^, İhsan Dağ^2^,

^1^Department of Clinical Psychology, Graduate School of Social Science, Ankara University, Ankara, Turkey

^2^Department of Clinical Psychology, Faculty of Letters, Hacettepe University, Ankara, Turkey.

Corresponding author: Erkan Eyrikaya^a^

**Email:**  [erkaneyrikaya@gmail.com](mailto:erkaneyrikaya@gmail.com)

**Author Contributions:** Erkan Eyrikaya designed research; performed research; analyzed data; and wrote the paper. İhsan Dağ is the supervisor of the thesis.

**Conflict of Interest:** The authors declare no competing interest. However, the publication fee for this article is covered by TÜBİTAK (The Scientific and Technological Research Council of Turkey).

Beier Sentence Completion Test (BSCT)

The Beier Sentence Completion Test (BSCT) is a semi-structured projective tool developed by the American psychologist Delton C. Beier to assess individuals' general attitudes, tendencies, and desires (Akkoyun, 2014). Unlike traditional sentence completion tests, the BSCT is distinguished by its extensive set of 67 prompts, each specifically designed to evaluate 13 distinct sub-dimensions of life (Akkoyun, 2014).The test comprises two versions, Form A and Form B, with Form B being applicable to individuals aged 16 and above. In the present study, Form B was administered to participants. The sub-dimensions assessed by the BSCT include: attitudes toward the past (five items), attitudes toward the future (five items), perceptions of self and personal abilities (six items), mother perception (five items), father perception (five items), attitudes toward home and family relationships (five items), attitudes toward friends (five items), attitudes toward authority (five items), fears and worries (five items), feelings of guilt (five items), attitudes toward school and work (five items), attitudes toward relationships with the opposite sex (five items), and general attitudes (six items). This comprehensive set of dimensions makes the BSCT uniquely suited for differentiating various psychopathologies, offering a depth of insight not typically found in other sentence completion tests (Akkoyun, 2014; Koç et al., 2021).

How to score BCST?

When scoring the BSCT test, we are interested in whether the sentence is negative, positive or neutral. In the evaluation of responses provided in the BCTT test, each incomplete sentence is marked as positive, negative, or neutral based on the options selected. The methodology proposed in the context of the emotional evaluation of these completions is as follows:

If the incomplete sentence is completed in a manner expressing healthy emotions, attitudes, interests, or similar, it should be marked as positive.

If the incomplete sentence is completed in a manner expressing unhealthy emotions, attitudes, interests, or similar, it should be marked as negative.

If the incomplete sentence is completed in a manner expressing neither healthy nor unhealthy emotions, attitudes, or interests, it should be marked as neutral. If the participant completes the sentence in a purely didactic way, the neutral option must also be marked. Additionally, if the sentence is left unfinished or blank, it should also be marked as neutral and categorized as meaningless.

**Group Creation Process**

Since BERT is a masked language model, the number of words and tokens varies across analyses. For SVM analyses, the number of words was limited based on the sample mean, whereas for BERT, the number of tokens was restricted. This distinction resulted in differences between the samples used for the SVM and BERT models. Consequently, the SVM analysis was considered a secondary, supporting study, as both the dataset composition and the analytical methodology differed from those of the BERT analysis. To account for these differences, preliminary analyses were conducted independently for both the BERT and SVM models.

The flow diagram illustrating the group formation process used in this research is presented in Fig. S1. The demographic characteristics of the sample for the BERT model in Study 1 are provided in Table S1, while the corresponding characteristics for the SVM model are presented in Table S2. Additionally, test sets for Study 2 were created based on the groups formed according to the diagram in Fig. S1, with details provided in Fig. S2 and S3.

**Preliminary analyzes for Study 1 (****PAS1)**

This section presents the preliminary analysis of Study 1.

**Evaluation of I-Talk and Negative Emotions.**

Detailed analyses were conducted to examine the distribution of first-person pronoun use (I-Talk) across groups and its relationship with negative emotionality. Initially, values from the Positive and Negative Affect Schedule (PANAS) were analyzed. While the increased statistical power associated with large samples often raises the likelihood of detecting statistically significant differences (Sullivan & Feinn, 2012), emotional state assessments focused on the significance of specific contrasts. These included the difference in PANAS-Negative scores between the Depression Group (Depression_G) and the Anxiety Group (Anxiety_G), as well as the difference in PANAS-Positive scores between the Control Group (Control_G) and the Anxiety Group.

Following this, analyses investigated whether significant differences in I-Talk use were present across all groups.

**Study 1: Assessment of Negative Emotionality for BERT.**

When examining the violin plot (Fig. S4) and the line graph (Fig. S5) from the BERT analysis in Study 1, distinct patterns in PANAS scores were observed across groups. The Anxiety Group (X̅ = 22.53) exhibited relatively lower PANAS-Negative values compared to the Depression Group (X̅ = 23.65), while the Depressive-Anxiety Group (X̅ = 29.39) reported the highest level of negative affect. A similar trend was observed for PANAS-Positive values; however, the Anxiety Group (X̅ = 33.30) reported relatively higher positive affect compared to the Control Group (X̅ = 31.88).

Outliers were identified in all groups except the Anxiety Group and examined in relation to scores on the Depression Anxiety Stress Scales (DASS-21) and the Brief Symptom Inventory (BSI). The outliers in the Control Group were consistent with typical control group data across both scales, and outliers in the pathology groups aligned with their respective group profiles. Therefore, no adjustments were made. As the Shapiro-Wilk test indicated that the data did not follow a normal distribution, the Mann-Whitney U test was employed for further analysis. No significant differences were found in PANAS-Negative scores between the Depression Group and the Anxiety Group (U = 15997, p > .05). Similarly, the difference in PANAS-Positive scores between the Control Group and the Anxiety Group was not significant (U = 16430.5, p > .05).

**Study 1: Evaluation of I-Talk for BERT.**

Word (token) counts were normalized based on standard deviation values, ensuring data consistency without requiring adjustments for outliers observed in the graphs (Fig. S6 and S7). This normalization process helped preserve data quality for subsequent analyses. To assess group differences in I_Talk_O_Count, the Shapiro-Wilk test was first applied to evaluate normality. Since the assumption of normality was not met, the Kruskal-Wallis H test was conducted. The analysis identified a significant difference in I_Talk_O_Count values among the groups (H(4) = 8.451, p = .037). Post hoc analysis using Dunn’s test revealed that this significance was driven by the difference between the Depression Group and the Anxiety Group (p < .05). Specifically, the Anxiety Group (X̅ = 6.86) exhibited relatively higher use of first-person pronouns compared to the Depression Group (X̅ = 5.61). However, the eta-squared value (η² = .004) indicated a very small effect size, suggesting that the observed difference was likely attributable to the large sample size rather than a meaningful effect.

For I_Talk_G_Count, the Shapiro-Wilk test again confirmed that the normality assumption was not met. Consequently, the Kruskal-Wallis H test was employed, which revealed no significant differences in I_Talk_G_Count values among the groups (H(4) = 4.93, p > .05).

**Study 1: Evaluation of Negative Emotionality for SVM.**

Negative emotionality was evaluated among the groups created for the SVM analysis in Study 1. Examination of the violin plot (Fig. S8) and the line graph (Fig. S5) revealed that the Anxiety Group (X̅ = 22.45) exhibited relatively lower PANAS-Negative values compared to the Depression Group (X̅ = 23.70), while the Depressive-Anxiety Group (X̅ = 29.43) reported the highest level of negative affect. A similar trend was observed for PANAS-Positive values, with the Anxiety Group (X̅ = 33.33) reporting slightly higher positive emotions compared to the Control Group (X̅ = 31.91).

Outliers identified in all groups except the Anxiety Group were further examined using the DASS-21 and BSI scores. Outliers in the Control Group were consistent with typical control group data across both scales, while outliers in the pathology groups aligned with their respective group profiles. Consequently, no adjustments were made.

To assess differences in emotional states, statistical analyses were conducted to evaluate the significance of PANAS-Negative scores between the Depression Group and the Anxiety Group, and PANAS-Positive scores between the Control Group and the Anxiety Group. The Shapiro-Wilk test indicated that PANAS-Negative scores for the Depression Group and Anxiety Group did not conform to a normal distribution. Thus, the Mann-Whitney U test was employed, which revealed no significant difference between these groups (U = 16002.5, p > .05). For PANAS-Positive scores between the Control Group and the Anxiety Group, the Shapiro-Wilk test confirmed normality, allowing for the use of a t-test. The analysis indicated that the difference was not significant (t = -1.948, p > .05).

**Study 1: Evaluation of I-Talk for SVM.**

For the SVM analysis in Study 1, I-Talk was evaluated across the generated groups (Fig. S8 and S5). Word counts were normalized based on standard deviation values, ensuring data consistency without requiring adjustments for outliers observed in the graphs. This approach preserved the integrity of the data for subsequent analyses. To assess group differences in I_Talk_O_Count, the Shapiro-Wilk test was conducted to evaluate the assumption of normality. As the normality criterion was not met, the Kruskal-Wallis H test was employed. The analysis revealed a significant difference in I_Talk_O_Count values among the groups (H(4) = 8.327, p = .039). Post hoc analysis using Dunn's test indicated that this significance was driven by differences between the Depression Group and the Anxiety Group (p = .035). Specifically, the Anxiety Group (X̅ = 6.89) used more self-referential statements than the Depression Group (X̅ = 5.63), albeit with a small magnitude. The eta-squared value (η² = .004) confirmed that the effect size was extremely small, suggesting that the observed difference is likely attributable to the sample size.

For I_Talk_G_Count, the Shapiro-Wilk test again indicated that the assumption of normality was not met, prompting the use of the Kruskal-Wallis H test. The analysis showed no significant differences in I_Talk_G_Count values among the groups (H(4) = 4.584, p > .05).

Overall, the analyses demonstrated that negative emotionality did not have a confounding effect across groups. The observed significance in I-Talk comparisons was likely attributable to the sample size rather than substantive group differences.

**Analysis**

This section provides information about the methodology used for data analysis**.**

**Computational Environment.**

All analyses were conducted in Python 3.8. BERT analyses utilized a V100 GPU, whereas SVM analyses were performed using Google’s standard CPU. All computations were executed using a Google Colaboratory PRO+ subscription.

**BERT Training.**

A pre-trained 128k cased BERT model (BERTurk)^[[2]](#footnote-2)^ from the Hugging Face library was fine-tuned using commonly applied parameters in the field. For Study 1, a learning rate of 2e-5, 8 epochs, and a batch size of 16 were configured with the Adam optimization algorithm. For Study 2, a learning rate of 2e-5, 5 epochs, and a batch size of 16 were applied with the same optimization algorithm. Both studies employed a 10-repeat, 10-fold stratified cross-validation method to ensure robust evaluation.

**SVM Training.**

For Study 1, a 2-repeat, 10-fold nested stratified cross-validation approach was utilized for both hyperparameter tuning and the outer loop . In Study 2, due to the inclusion of four distinct test sets, a 10-fold nested cross-validation method was employed alongside parameter tuning (see Fig. S9). Parameter selection was based on commonly used configurations in the field. A Grid Search with cross-validation was conducted using the f1_weighted metric, allowing variations in the C and Gamma parameters: For Study 1, both C and Gamma parameters were adjusted in multiples of 10, with C ranging from 0.01 to 100 and Gamma from 0.0001 to 0.01, including the 'scale' option. The 'ovo' (one-vs-one) parameter was used as the decision function. For Study 2, values in multiples of 10 were used, ranging from 0.001 to 100 for C, and from 0.0001 to 1 for Gamma. Both the Radial Basis Function (RBF) and linear kernels were preferred for Study 1 and Study 2.

Class weights were calculated using the sklearn library for both BERT and SVM models in Study 1 to address class imbalance and ensure adequate representation of minority classes.

**XAI-SHAP Analysis.**

To interpret the model outputs, the SHAP (SHapley Additive exPlanations) library, regarded as the gold standard for model interpretability, was employed (Lundberg & Lee, 2017). SHAP quantifies the contribution of individual features to the model's predictions, identifying key factors and their impact. The PartitionExplainer was utilized to explain BERT model outputs (see Tables S3–S5 for Study 1; Table S6 and S7 for Study 2)^[[3]](#footnote-3)^, while the KernelExplainer was used for SVM models (global feature importance for Study 1, see Fig. S10; the SHAP graph for the SVM model in Study 2, see Fig. S11).

**Results.**

The other performance metrics obtained from model training are summarized in Tables S8–S12. Additionally, Fig. S12 illustrates the AUC-ROC curves for the SVM and BERT models in Study 2, demonstrating highly comparable performance between the two approaches. Also, confusion matrices for Study 1 are given in Fig. S13.

To further investigate the role of I-Talk variables, SVM models were retrained using the same data, replacing the I_Talk_O_Count variable with I_Talk_G_Count. The results revealed minimal performance differences between Turkish-specific explicit "I" (I_Talk_O_Count) and the combined explicit and implicit I-Talk (I_Talk_G_Count). These findings underscore I-Talk as a robust marker of anxiety (see Fig. S14).


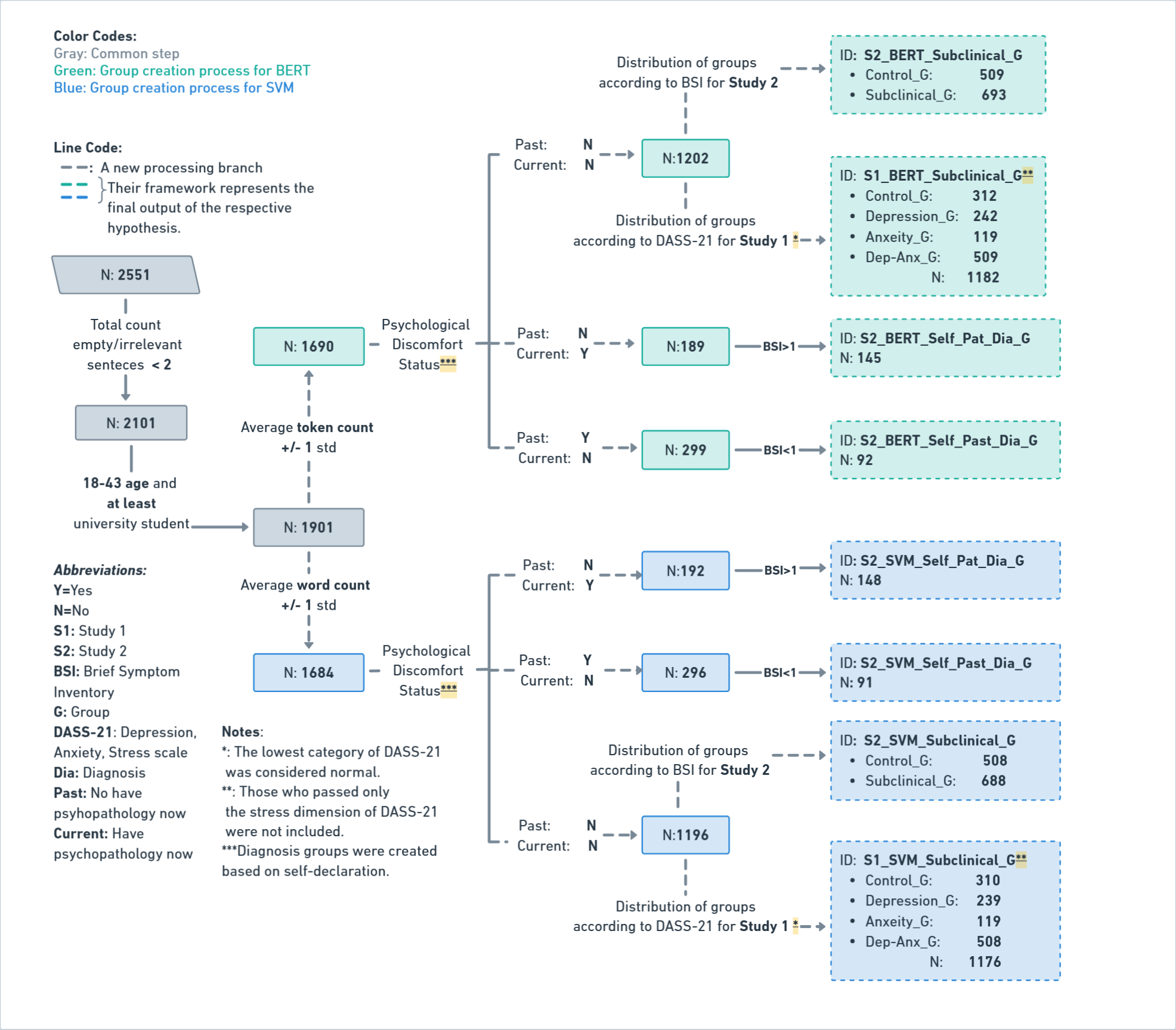


**Fig. S1.** Flow diagram of the path followed in the process of creating groups. Green indicates the groups belonging to the Bert model and blue indicates the groups belonging to the SVM model. The gray ones are the common steps.

**
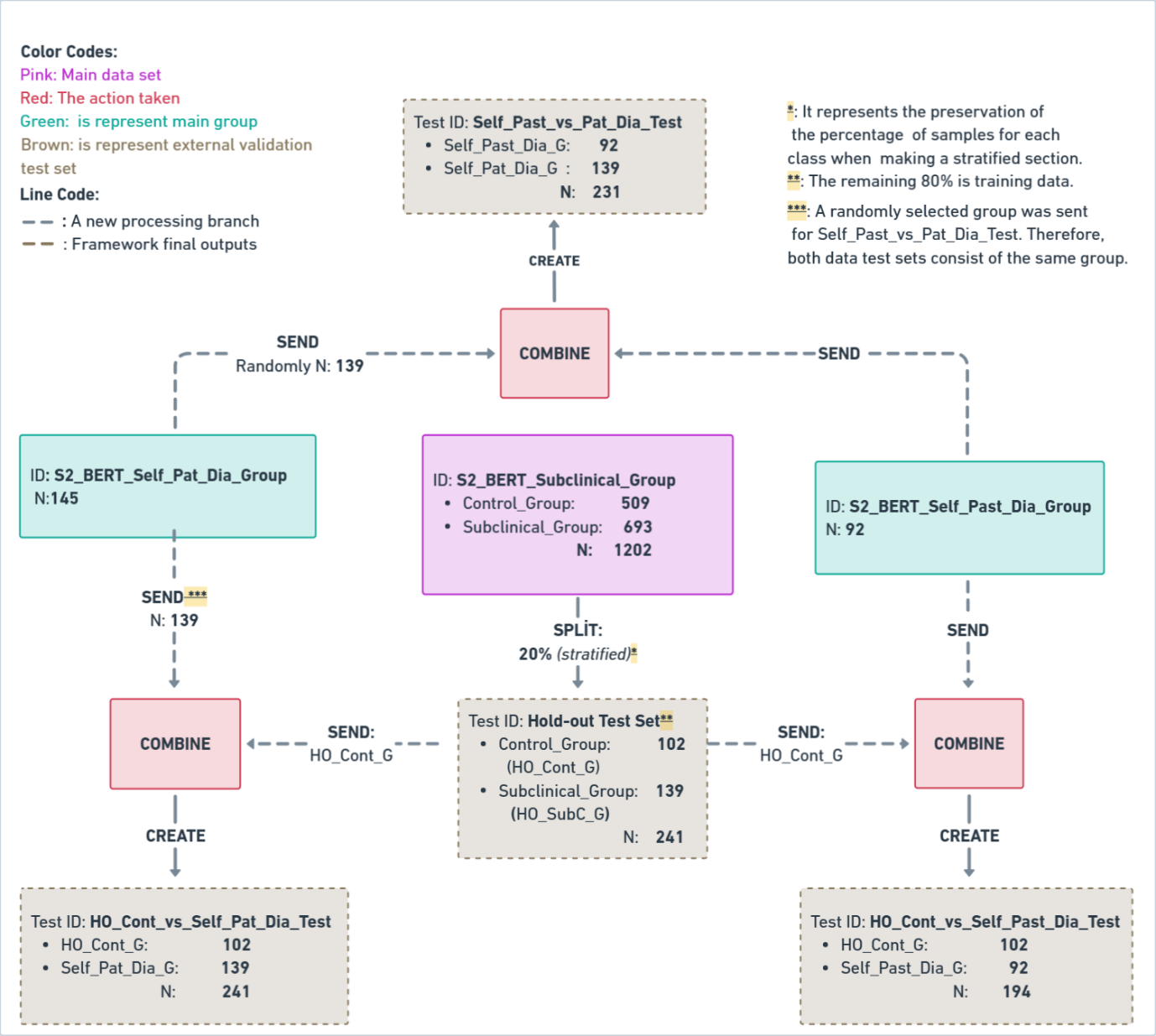
Fig. S2.** It shows the creation of external test sets for the BERT model in Study 2.


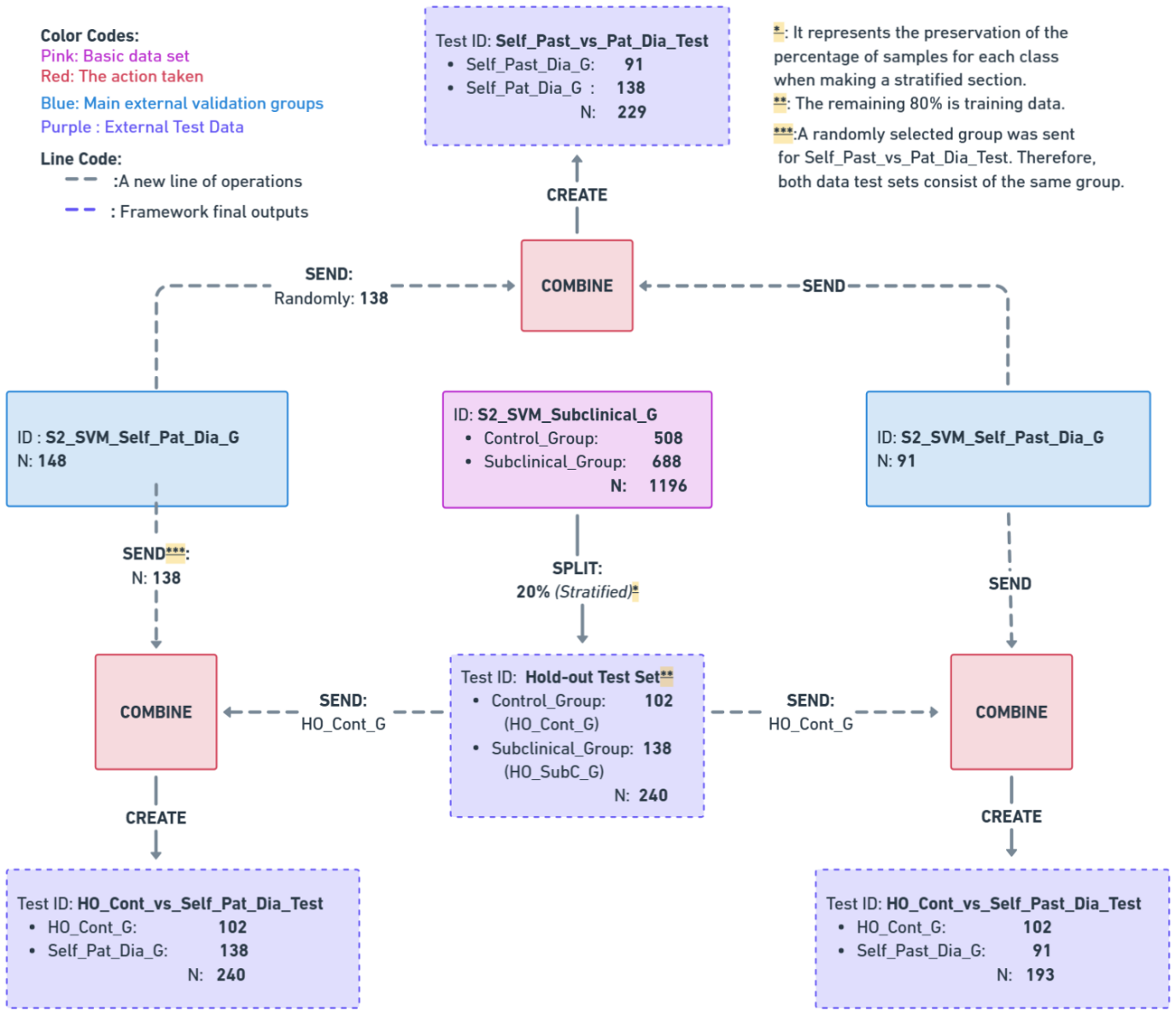


**Fig. S3.** It shows the creation of external test sets for the SVM model in Study 2.


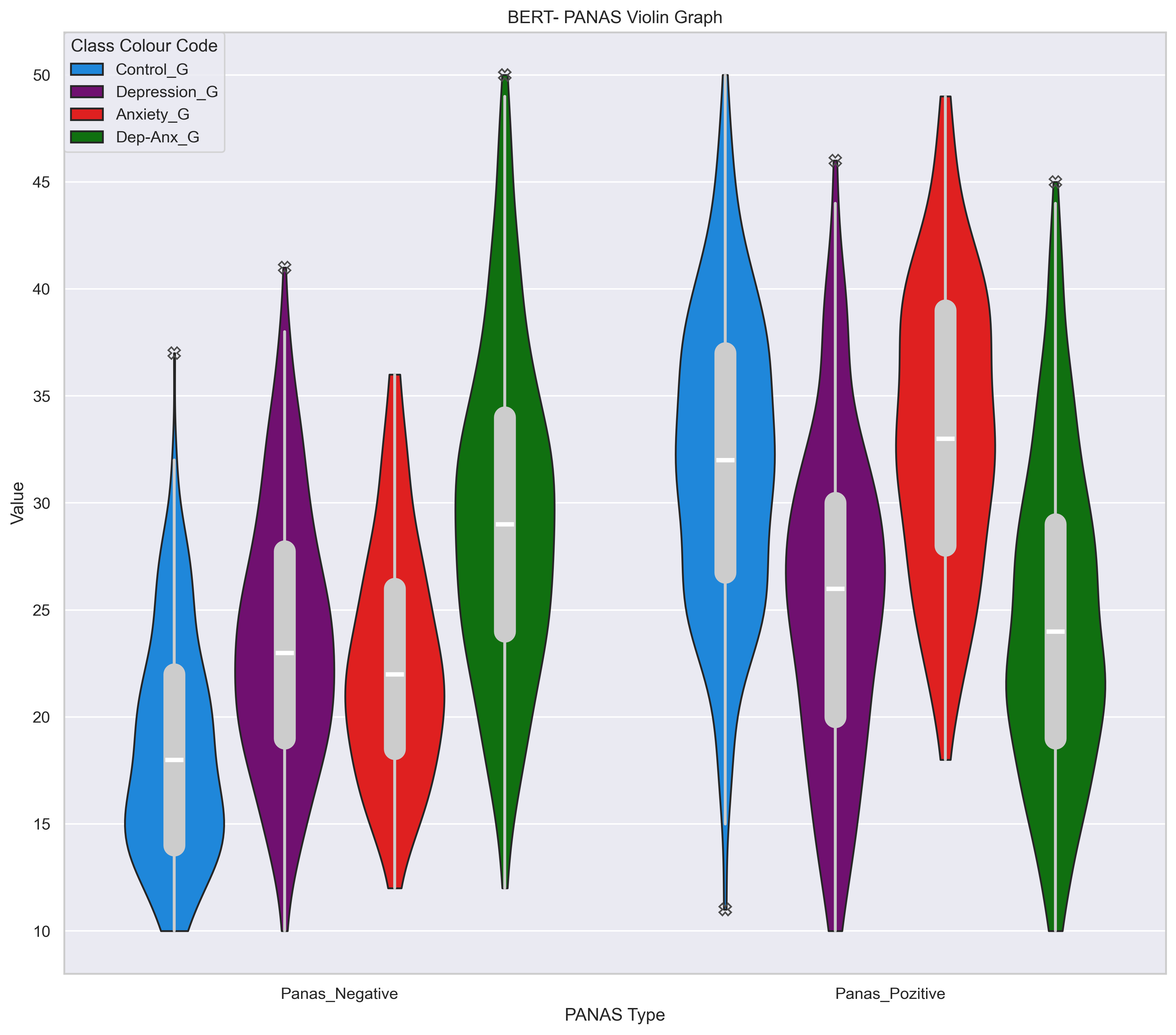


**Fig. S4.** It illustrates the distribution of PANAS scores for Control, Depression, Anxiety, and Dep-Anx groups in Study 1 for the BERT analyses. Violin plots visualize the spread and density of scores for PANAS_Negative and PANAS_Positive categories. Extreme values are marked with stars for each group.


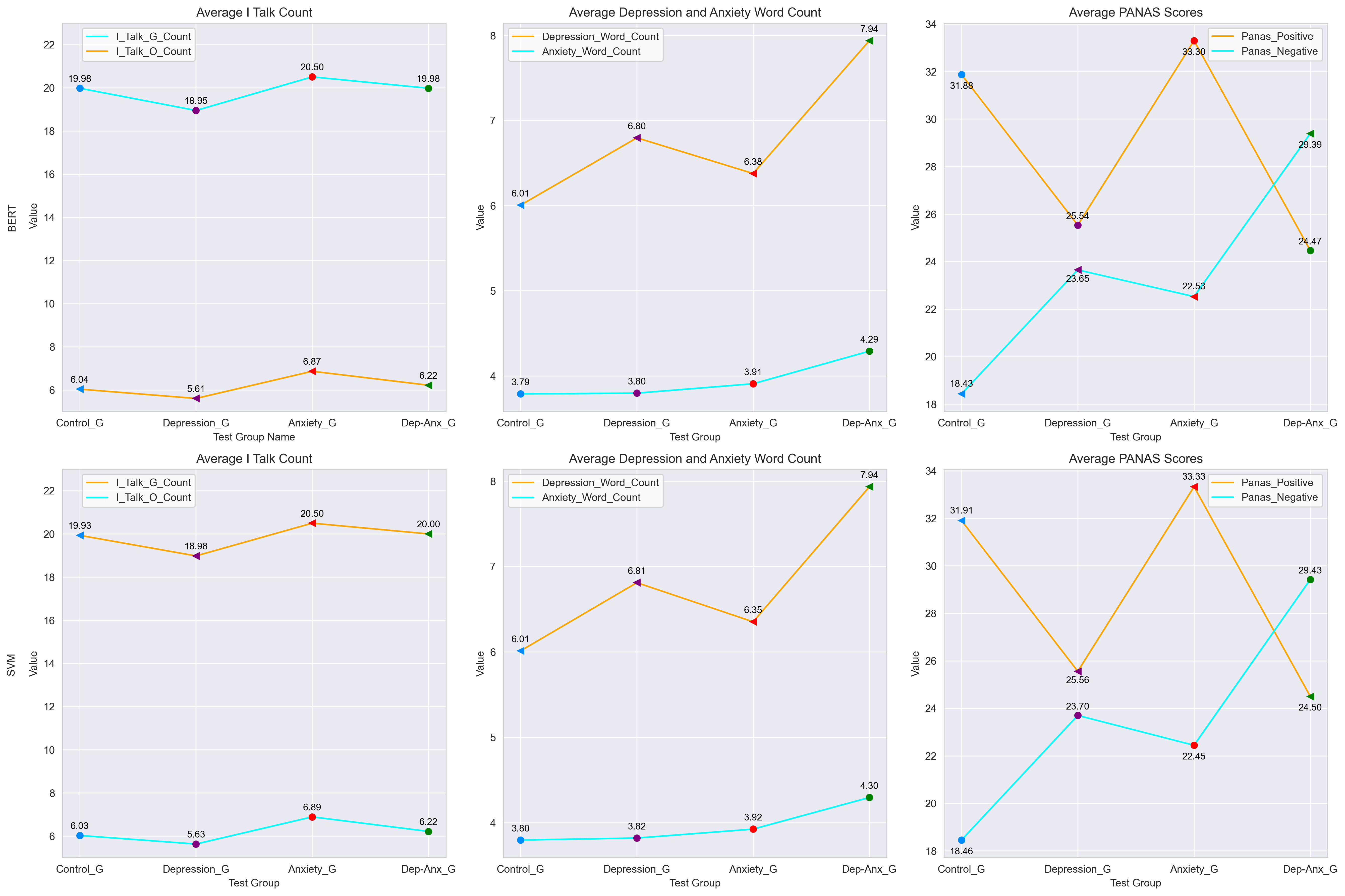


**Fig. S5.** It compares the average values of variables (I_Talk_Count, Depression and Anxiety Word Count, PANAS scores) across Control, Depression, Anxiety, and Dep-Anx groups in Study 1. The top row represents average value in the group for the BERT analyses, and the bottom row represents average value in the group for the SVM analyses. Trends highlight group-specific differences for each variable.


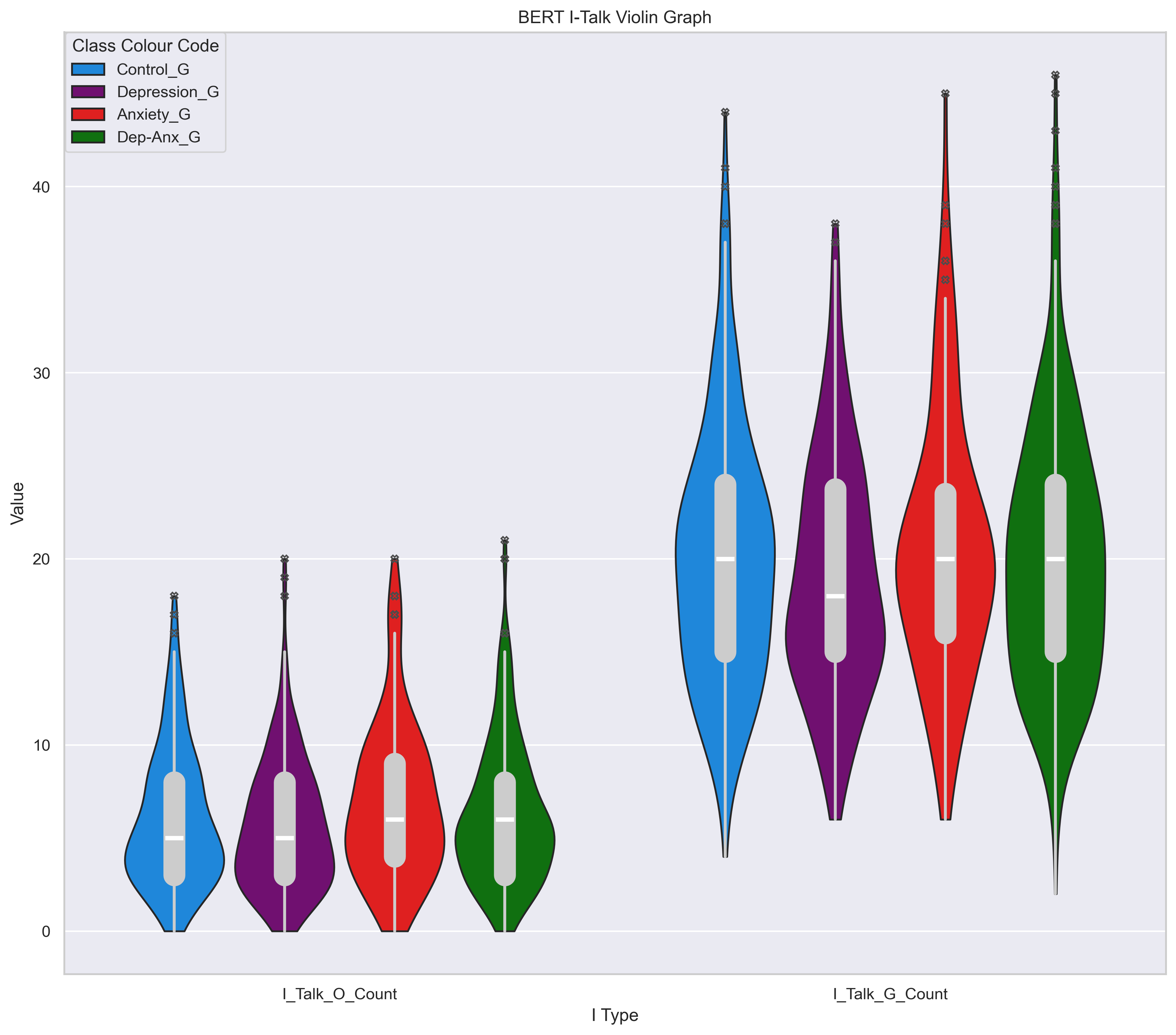


**Fig. S6.** It illustrates the distribution of I-Talk scores for Control, Depression, Anxiety, and Dep-Anx groups in Study 1 for the BERT analyses. Violin plots visualize the spread and density of scores for I_Talk_O_Count and I_Talk_G_Count categories. Extreme values are marked with stars for each group.


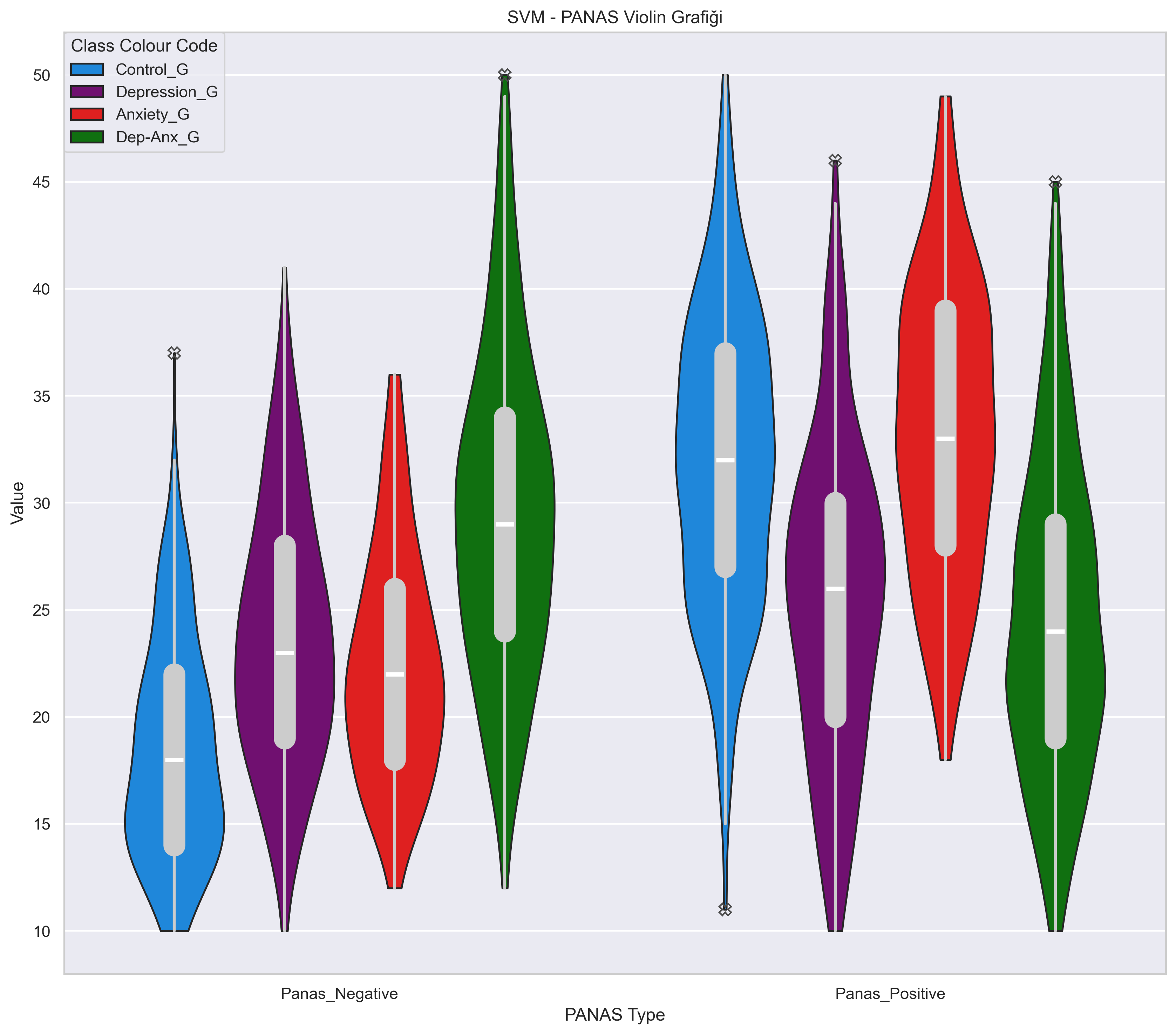


**Fig. S7.** It illustrates the distribution of PANAS scores for Control, Depression, Anxiety, and Dep-Anx groups in Study 1 for the SVM analyses. Violin plots visualize the spread and density of scores for PANAS_Negative and PANAS_Positive categories. Extreme values are marked with stars for each group.


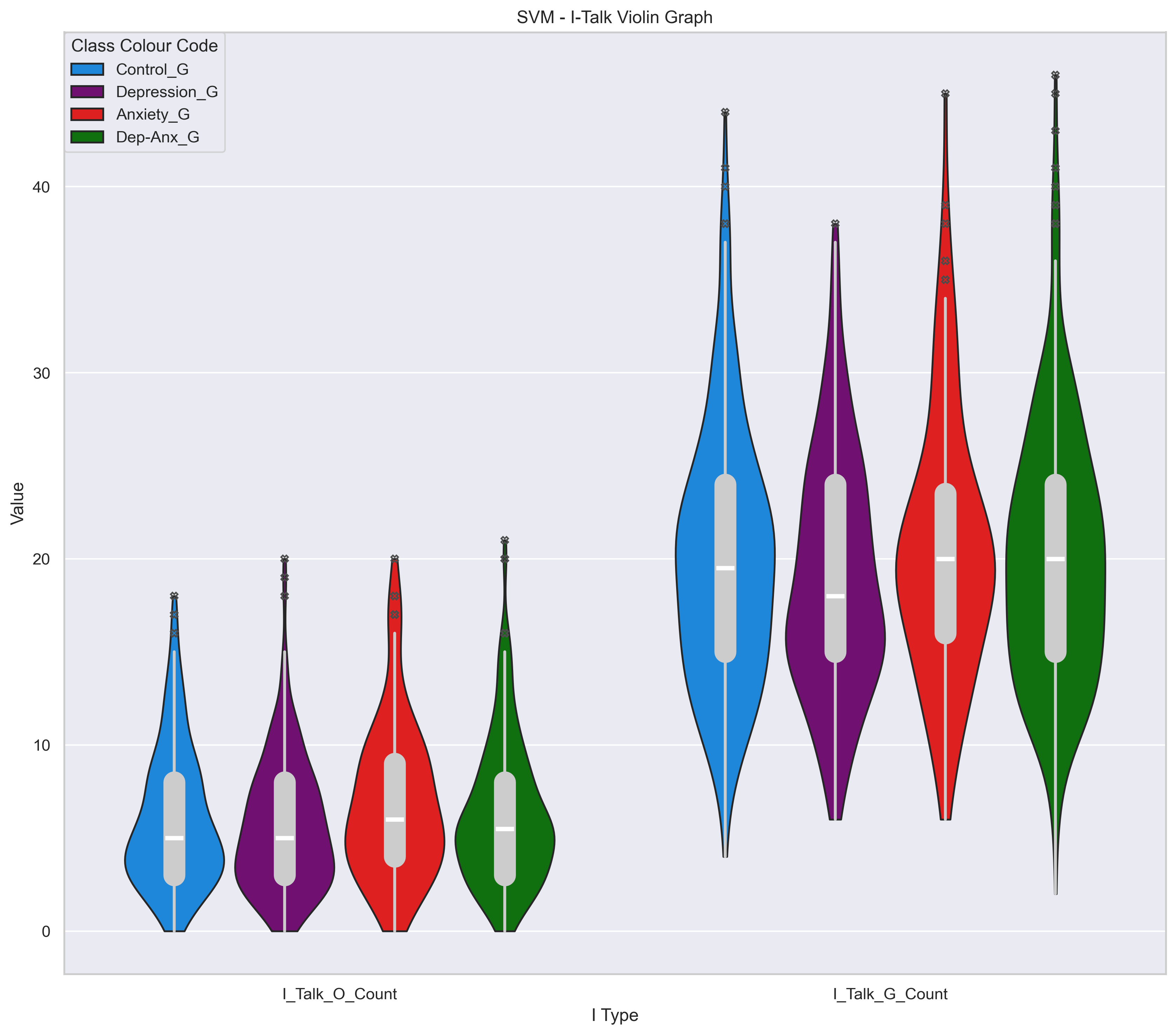


**Fig. S8.** It illustrates the distribution of I-Talk scores for Control, Depression, Anxiety, and Dep-Anx groups in Study 1 for the SVM analyses. Violin plots visualize the spread and density of scores for I_Talk_O_Count and I_Talk_G_Count categories. Extreme values are marked with stars for each group.


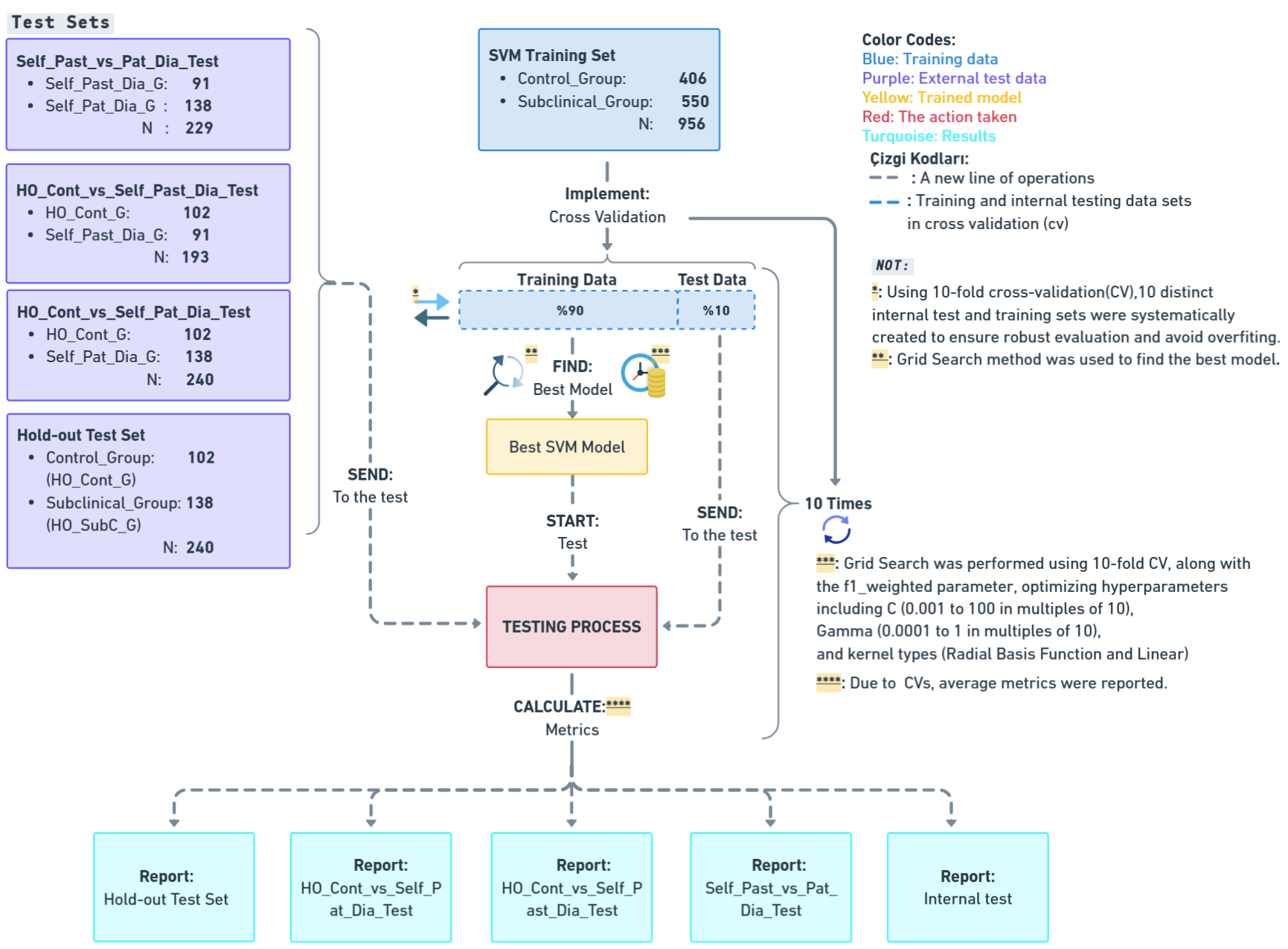


**Fig. S9.** Shows the SVM analysis of Study 2.

**
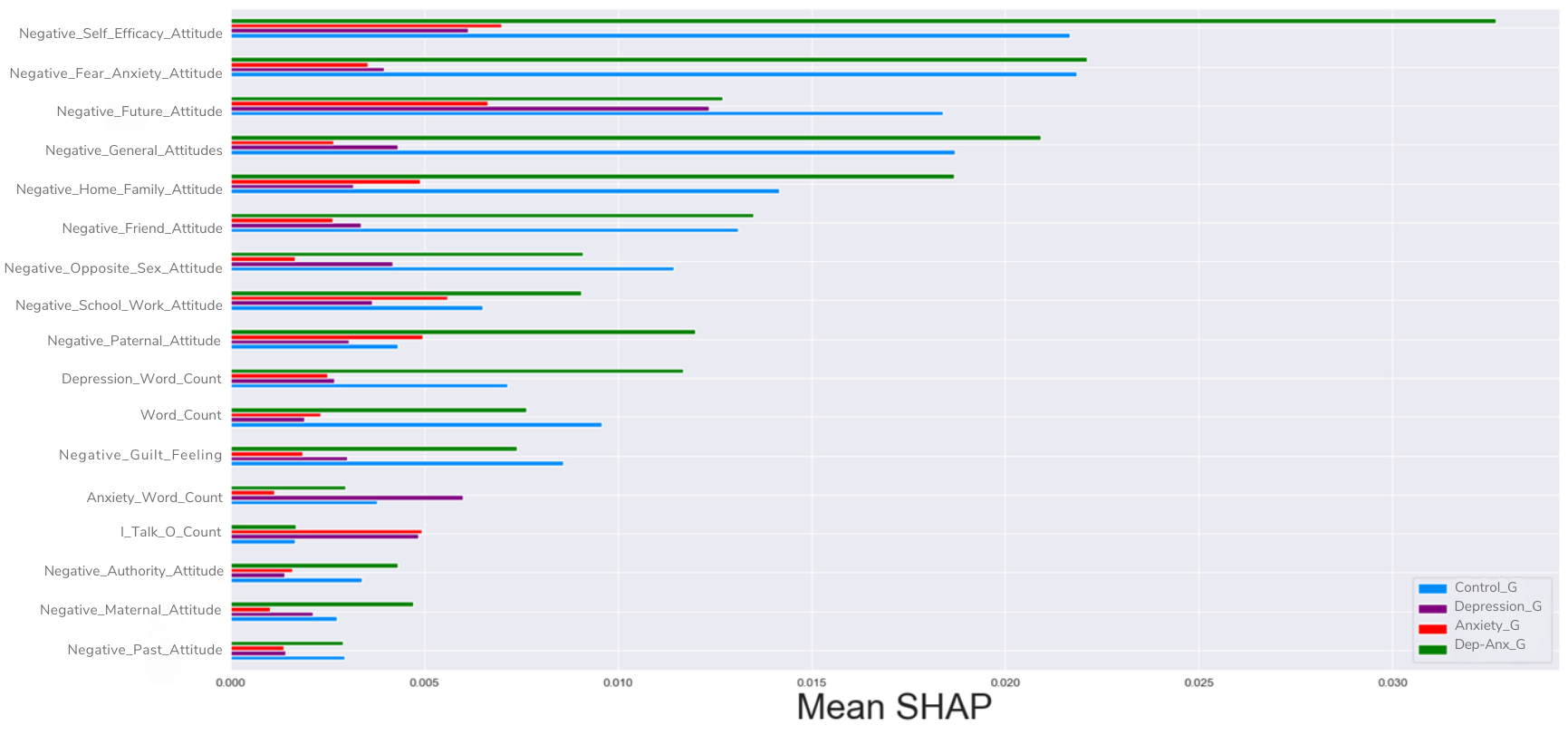
**

**Fig. S10.** This graph is the global features importance graph for the SVM analysis of Study 1. They are listed according to the feature that is most effective in the decision-making of the model and their distribution is given according to classes.


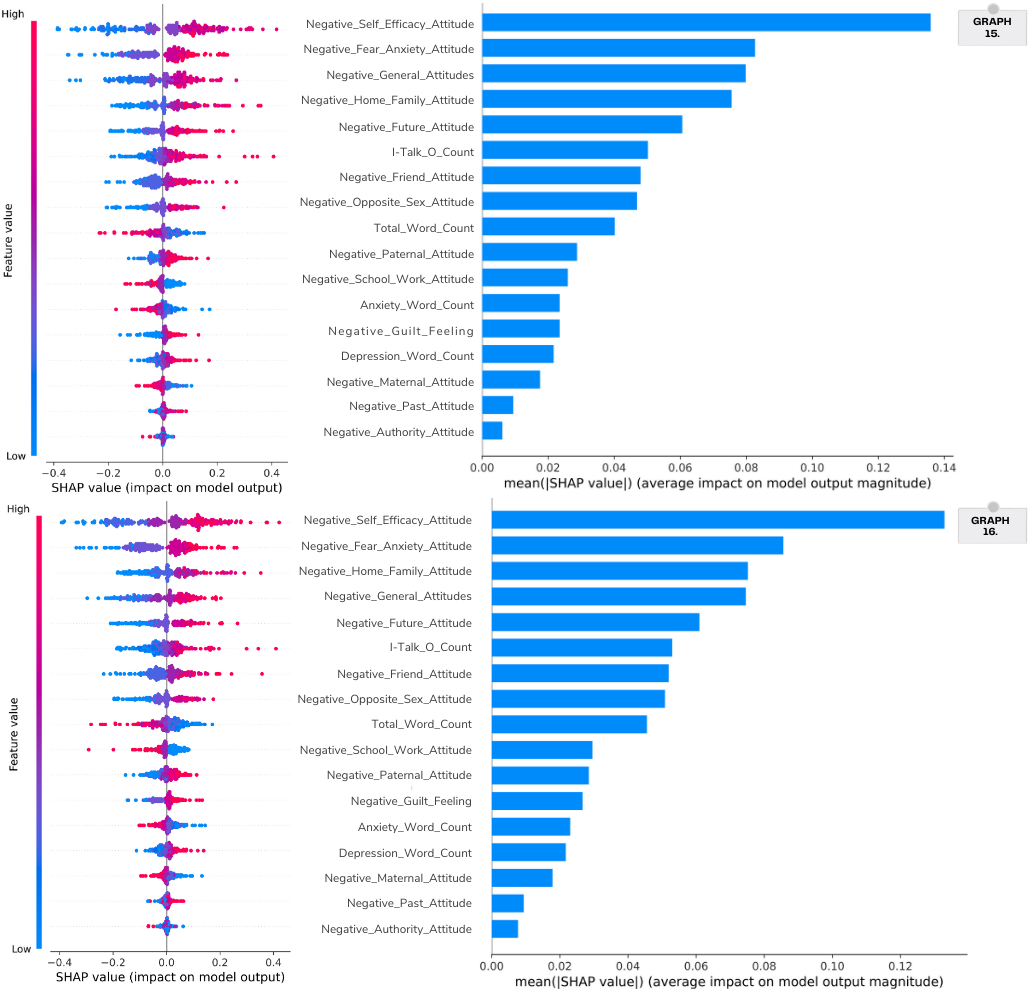
**Fig. S11.** The above graph was generated by explaining the SVM model from Study 2. The top graph represents the model explanation for the internal test set. The bottom graph corresponds to the external validation set (HO_Cont_vs_Self_Pat_Dia_Test), created with the control group and the self-reported pathology diagnosis group from the external set. In the scatter plot, the right side of the y-axis represents the subclinical pathology group for the top graph and the self-reported pathology diagnosis group for the bottom graph. Red dots indicate high values of the corresponding feature, while blue dots represent low values. Each dot in the graph corresponds to a data point. For example, an increase in I-Talk values predicts the pathology group in both test sets, while decreasing values predict the control group. The bar charts show the global importance of the respective features.


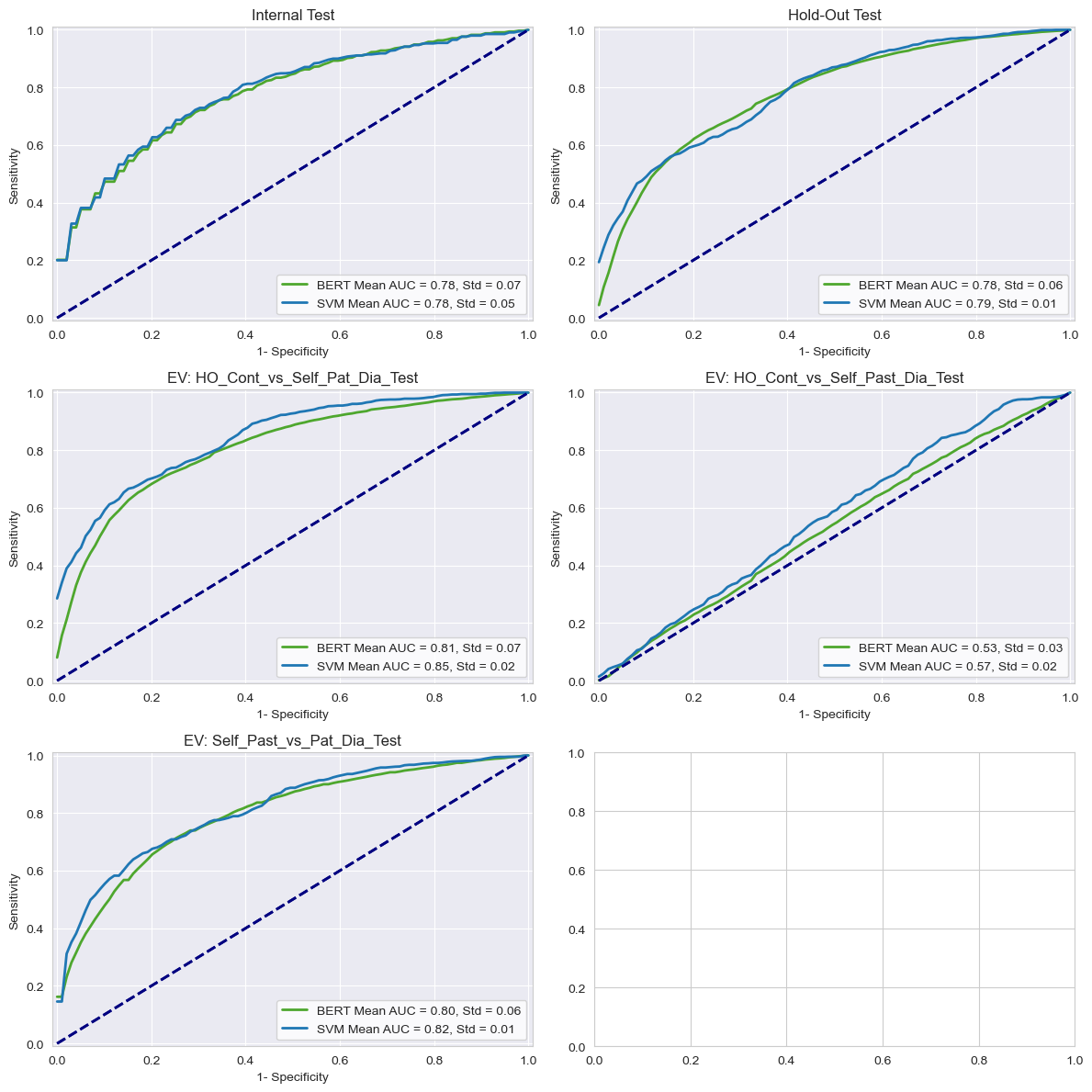


**Fig. S12.** This figure presents the AUC-ROC values for SVM (using the I_Talk_O_Count variable) and BERT models in Study 2. The graphs compare model performance across different tests, including internal, hold-out, and evaluation tests. The results highlight the mean AUC values and standard deviations for both models under various testing conditions.


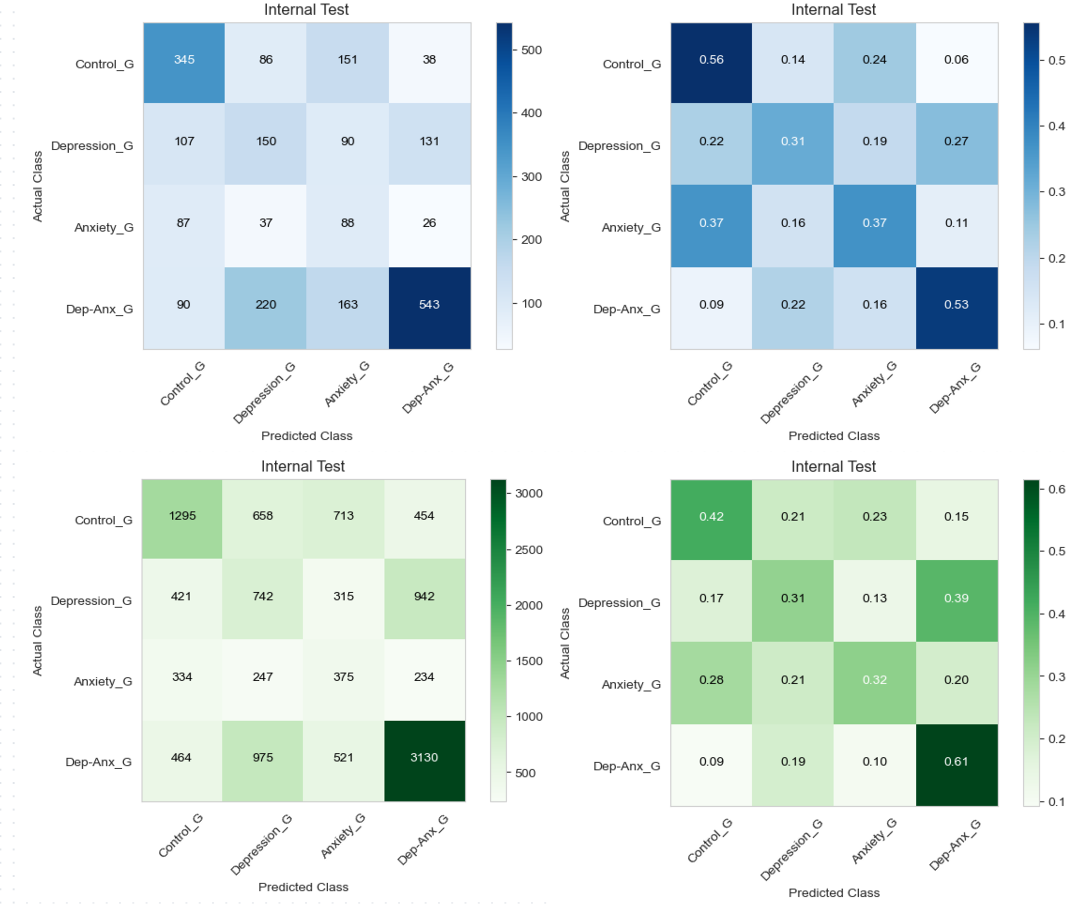


**Fig. S13.** The graph shows the confusion matrix for the models resulting from cross-validation for Study 1. Blue shows the matrix for SVM analysis, green shows the confusion matrix for BERT analysis. The normalized confusion matrices are those on the right.

**
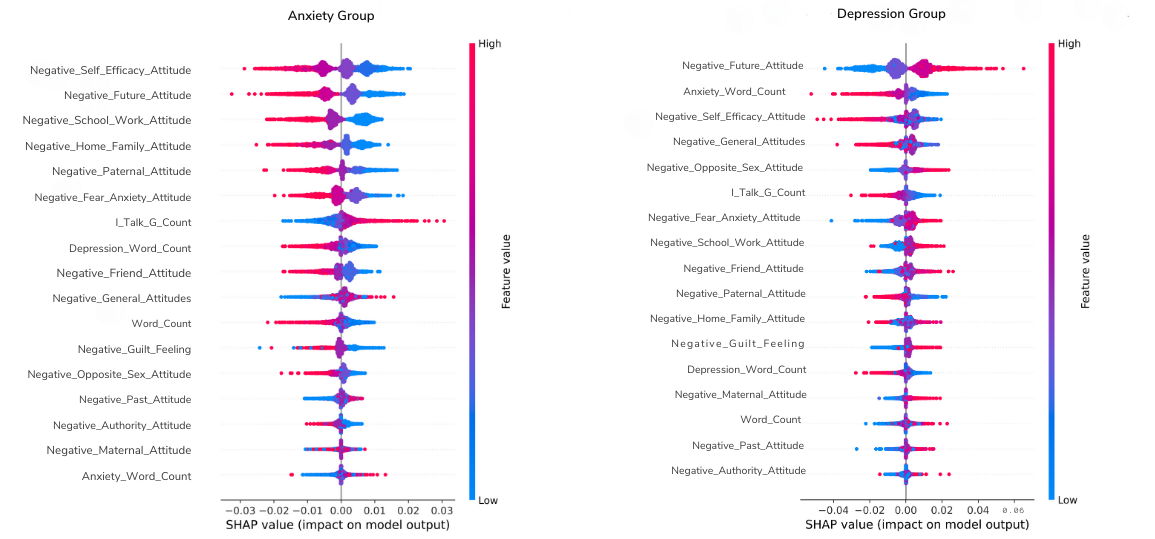
**

**Fig. S14.**  It illustrates the locally aggregated SHAP values for the SVM models trained in Study 1, highlighting the impact of features like I_Talk_G_Count on the probability of belonging to the Anxiety and Depression groups. Red dots indicate high feature values, while blue dots represent low values, showing that increasing I-Talk values are associated with a higher likelihood of belonging to the Anxiety group.


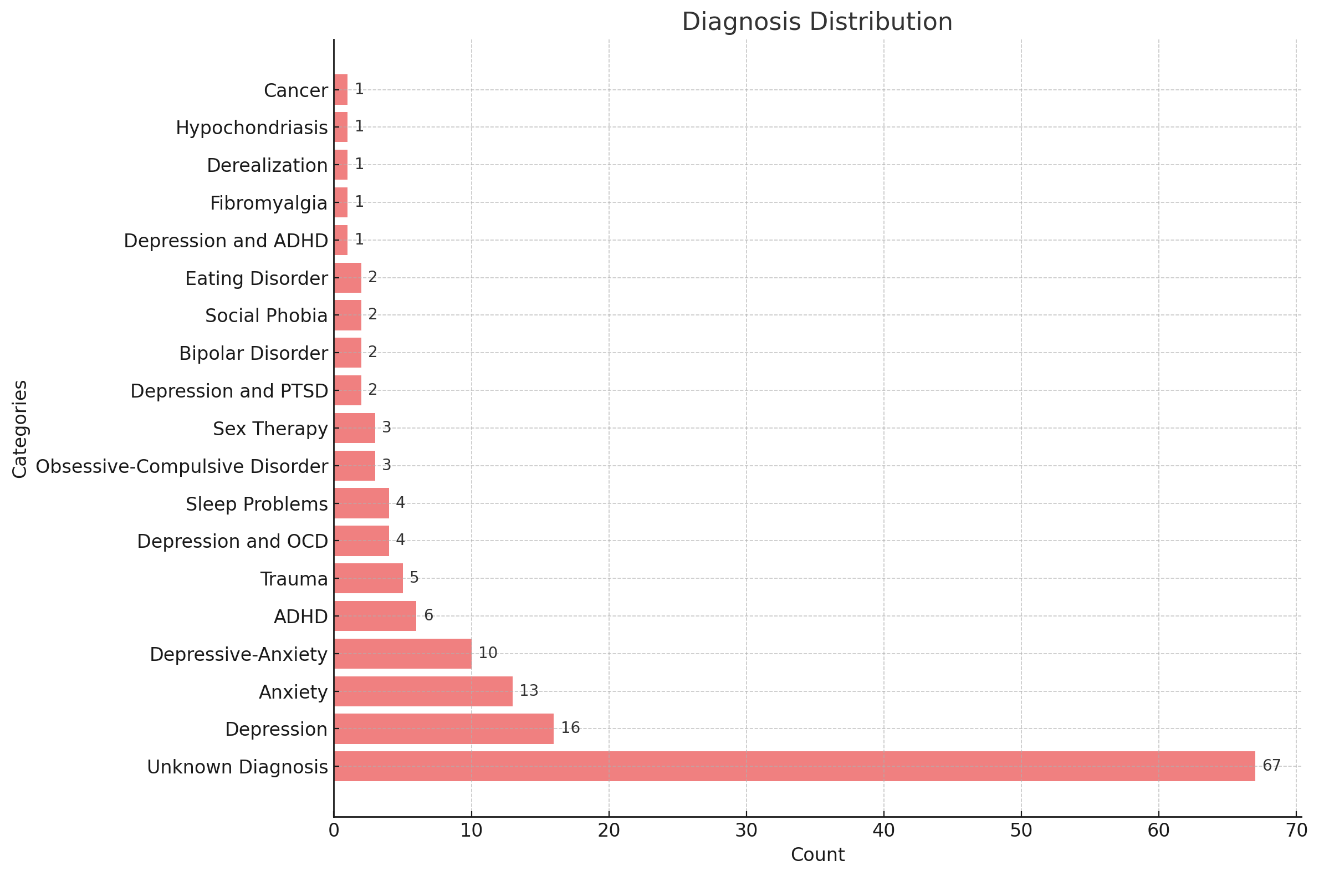


**Fig. S15.** It shows the self-reported diagnoses of the pathology groups in Study 2. While more than half of the participants (67) did not specify a direct diagnosis (classified as 'Unknown Diagnosis'), the remaining participants reported conditions such as Depression (16), Anxiety (13), Depressive-Anxiety (10), and other less frequent diagnoses.

**
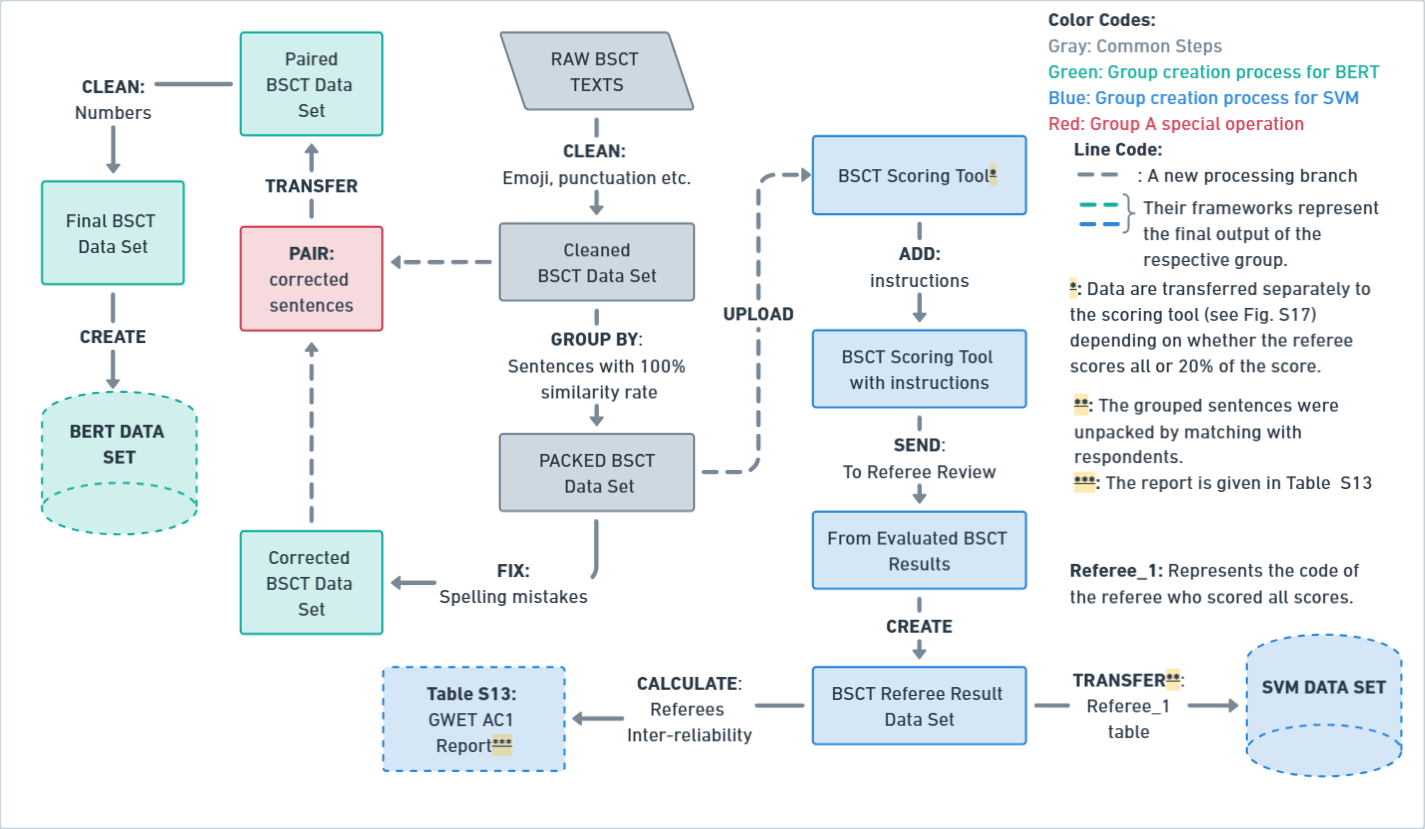
**

**Fig. S16.** It shows the workflow for scoring the collected data by the referees and preparing the data for analysis.


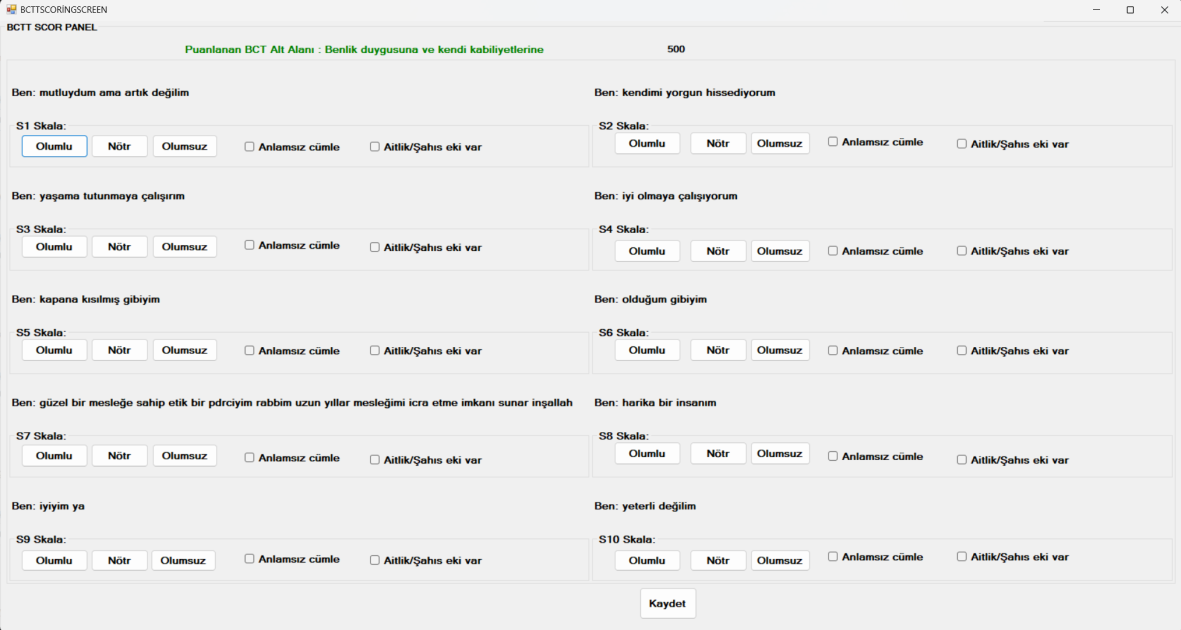


**Fig. S17.** This graphic shows the BCST scoring tool prepared to facilitate referees' scoring and sample sentences. The interface allows referees to assign scores (Positive, Neutral, Negative, Unintelligible).


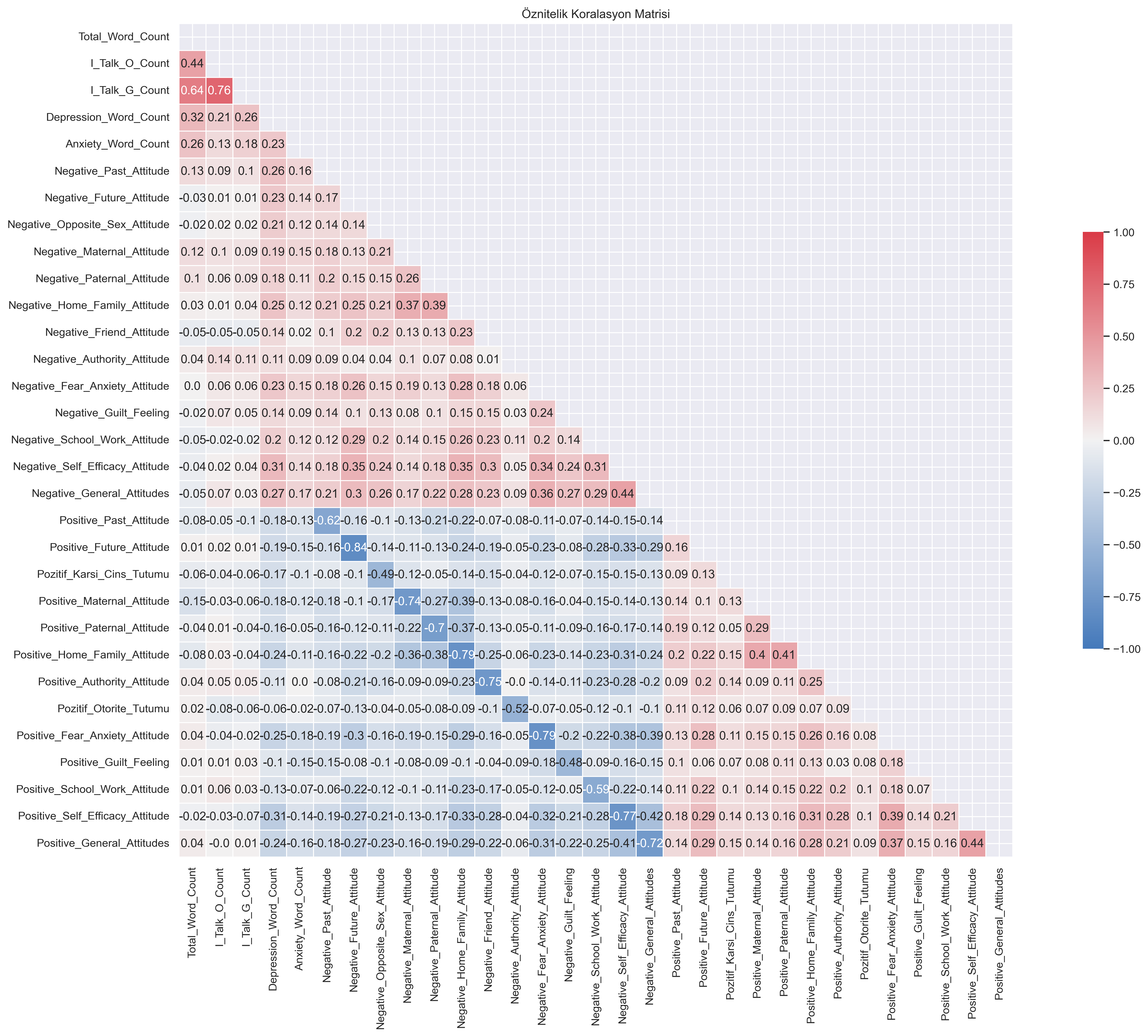


**Fig. S18.** Evaluation of multicollinearity Correlation matrix for the SVM analyses. The heatmap uses a color gradient from red (positive correlation) to blue (negative correlation), with values ranging from -1 to 1. Strong correlations (either positive or negative) are highlighted, indicating potential multicollinearity concerns that may affect the SVM model's performance. This visualization helps identify highly correlated variables to address collinearity issues in the dataset.


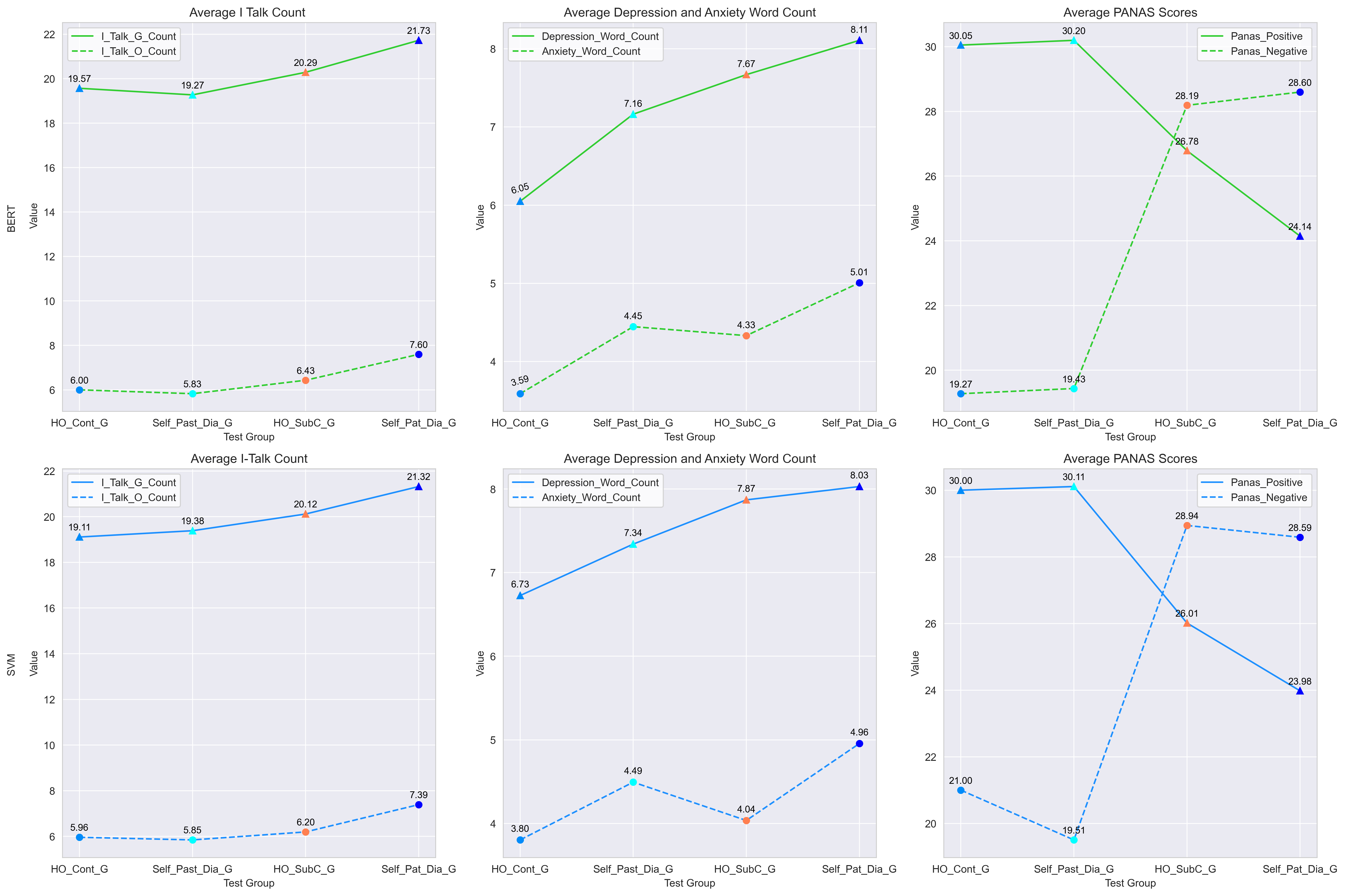


Fig. S19. It compares the average values of variables (I-Talk Count, Depression and Anxiety Word Count, PANAS scores) across HO_Cont_G, Self_Past_Dia_G, HO_SubC_G, and Self_Pat_Dia_G groups in Study 2. The top row represents average value in the group for the BERT analyses, and the bottom row represents average value in the group for the SVM analyses. Trends highlight group-specific differences for each variable.


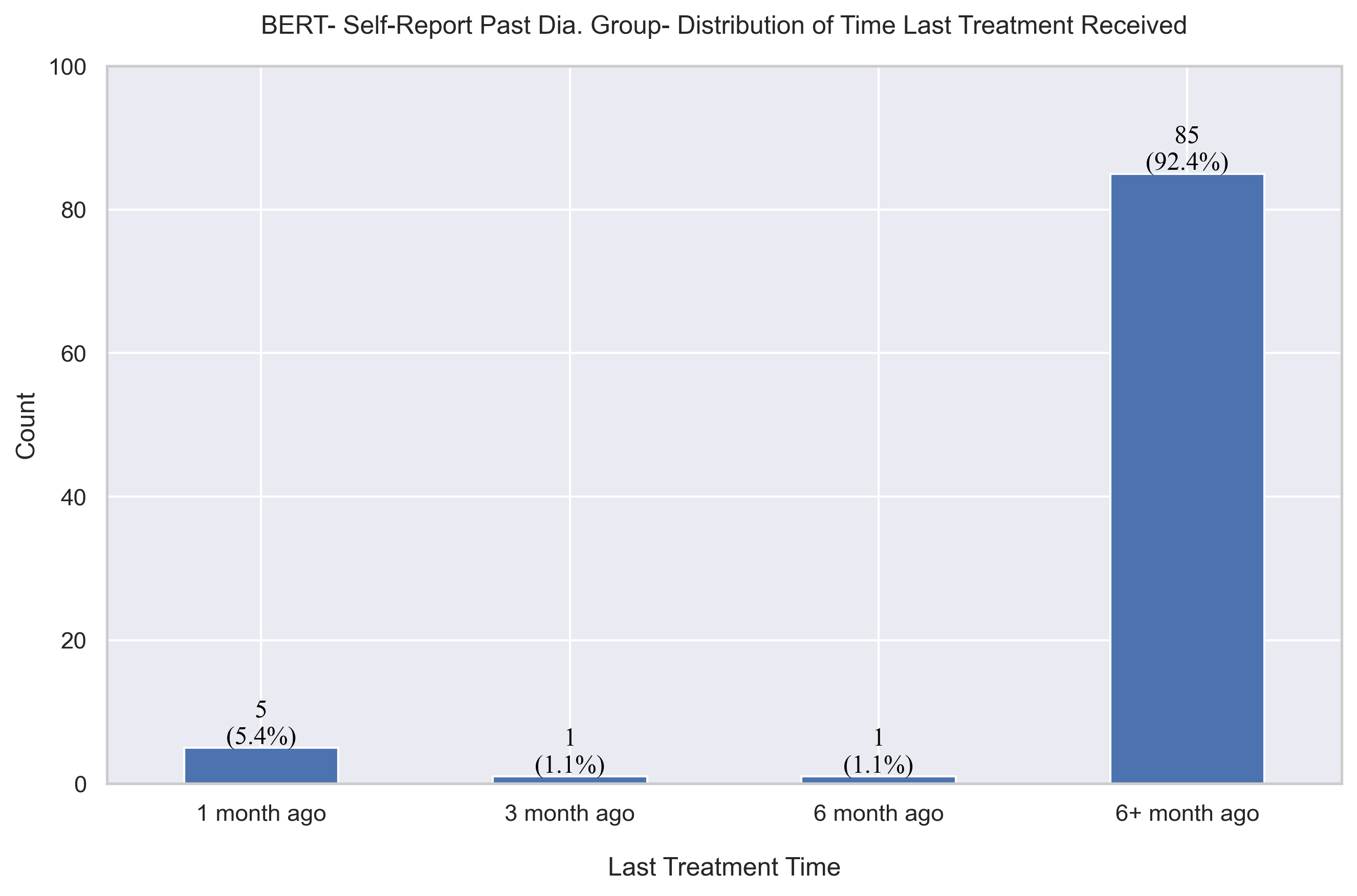


**Fig. S20.** It shows a bar graph depicting the distribution of the time since the last treatment for the self-reported past diagnosis group created for the BERT analysis in Study 2. The majority (92.4%) received their last treatment over six months ago, with smaller proportions receiving treatment within the last month (5.4%) or three to six months ago (1.1% each).


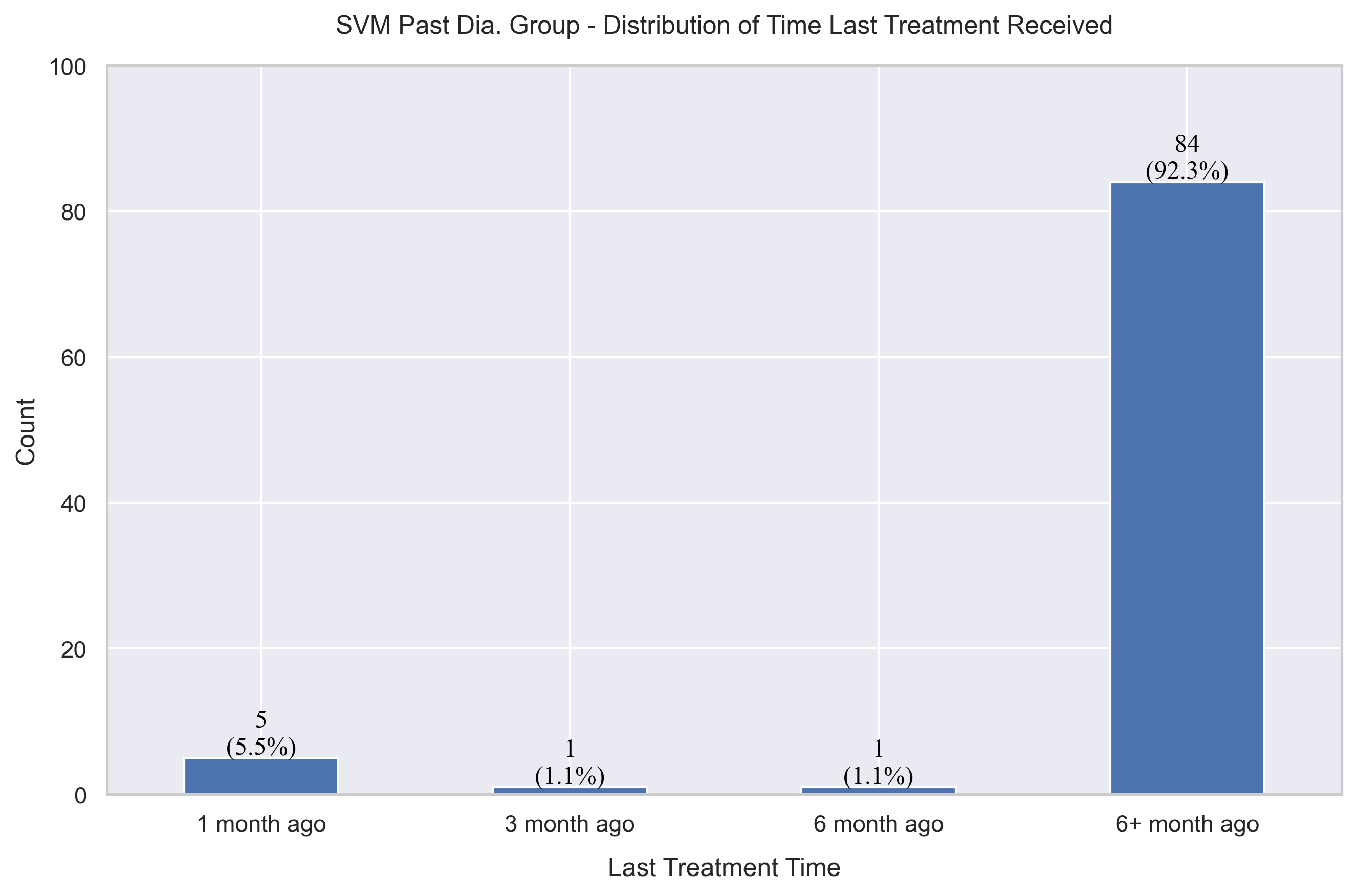


**Fig. S21.** It displays a bar graph illustrating the distribution of the time since the last treatment for the self-reported past diagnosis group created for the SVM analysis in Study 2. The majority (92.3%) received their last treatment over six months ago, with smaller proportions receiving treatment within the last month (5.5%) or three to six months ago (1.1% each).


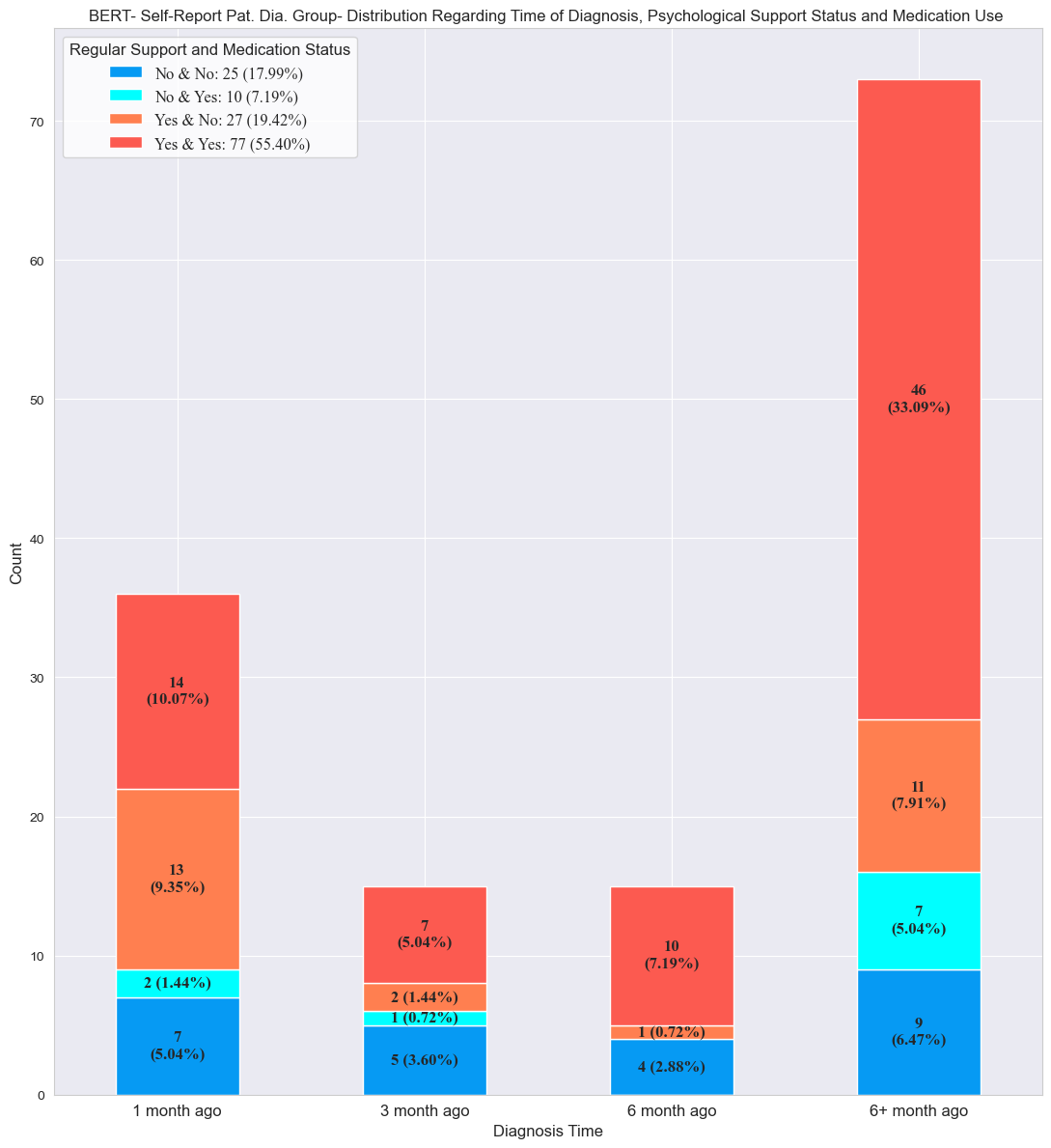


**Fig. S22.** **It** shows the distribution of current treatment status within the self-reported pathology diagnosis group based on the time since diagnosis, psychological support status, and medication use in Study 2 for the BERT analyses. The bar graph categorizes participants into four groups: no support/no medication, no support/yes medication, yes support/no medication, and yes support/yes medication, with the majority receiving both regular support and medication, particularly those diagnosed more than six months ago.


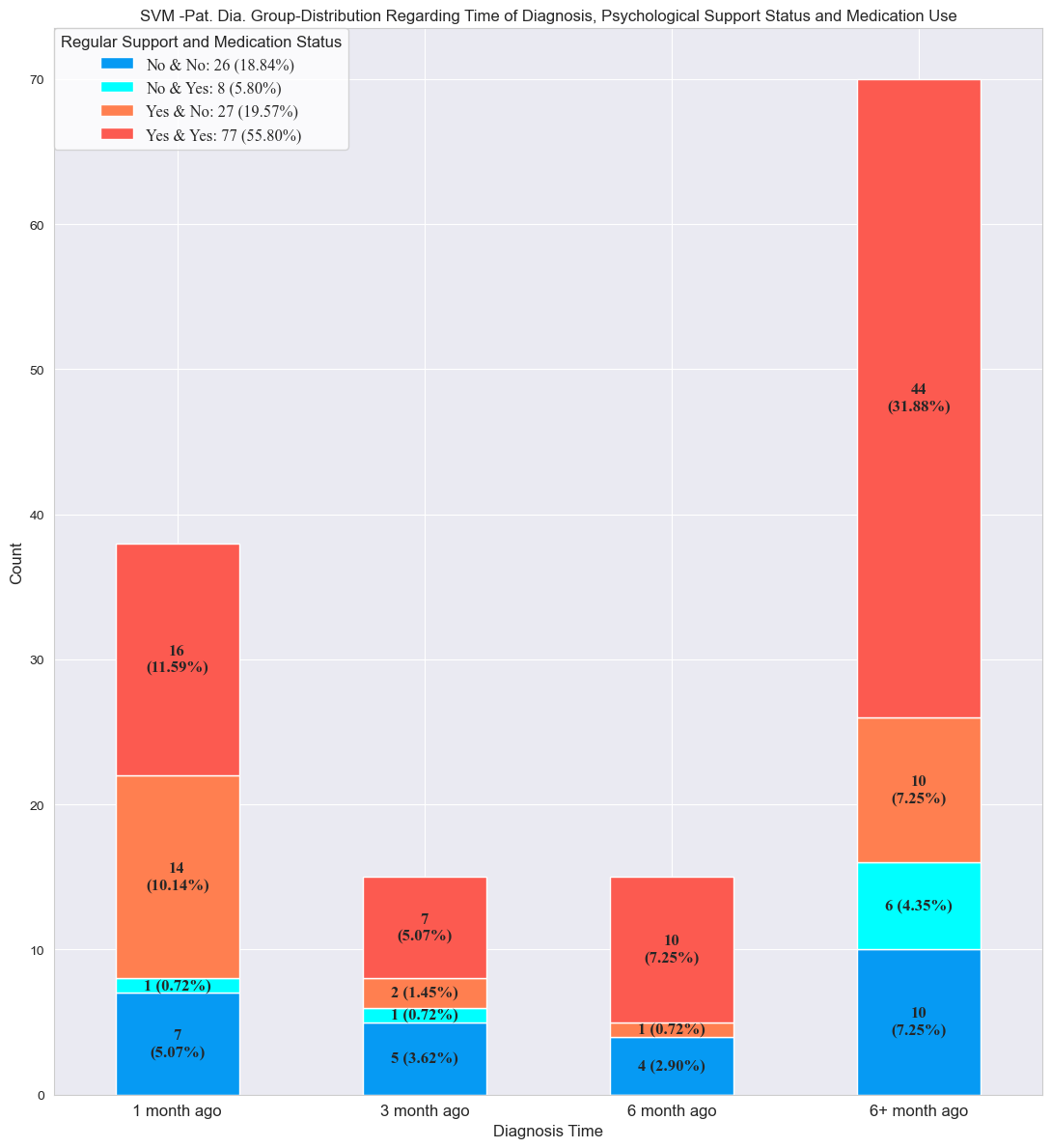


**Fig. S23.** **It** presents the distribution of current treatment status in the self-reported pathology diagnosis group based on time since diagnosis, psychological support status, and medication use for the SVM analysis in Study 2 for the SVM analyses. Participants are divided into four categories: no support/no medication, no support/yes medication, yes support/no medication, and yes support/yes medication, with the majority receiving both support and medication, especially those diagnosed over six months ago.

**Table S1.** Shows the demographic characteristics of the sample for the BERT model in Study 2

| **ID:** S2_BERT_Subclinical_Group | | Control Group | | Subclinical Group | |
| --- | --- | --- | --- | --- | --- |
|  |  | *N* | % | *N* | % |
| Gender | Women | 345 | 68 | 522 | 75 |
|  | Men | 164 | 32 | 171 | 25 |
| Age | 18-29 | 367 | 72 | 579 | 84 |
|  | 30-43 | 142 | 28 | 114 | 16 |
| Income  Level | Lower-Middle | 31 | 06 | 74 | 11 |
|  | Middle | 172 | 34 | 297 | 43 |
|  | Middle -Upper | 306 | 60 | 322 | 46 |
| Education  Level | Associate's or Bachelor's Degree | 434 | 85 | 607 | 88 |
|  | Master Degree | 60 | 12 | 73 | 10 |
|  | Doctorate Degree | 15 | 03 | 13 | 02 |

**Table S2.** Shows the demographic characteristics of the sample for the SVM model in Study 2

| **ID:**  S2_SVM_ Subclinical_Group | | Control Group | | Subclinical Group | |
| --- | --- | --- | --- | --- | --- |
|  |  | *N* | % | *N* | % |
| Gender | Women | 345 | 68 | 520 | 76 |
|  | Men | 163 | 32 | 168 | 24 |
| Age | 18-29 | 365 | 72 | 575 | 84 |
|  | 30-43 | 143 | 28 | 113 | 16 |
| Income  Level | Lower-Middle | 32 | 06 | 75 | 11 |
|  | Middle | 172 | 33 | 294 | 43 |
|  | Middle -Upper | 304 | 60 | 319 | 46 |
| Education  Level | Associate's or Bachelor's Degree | 433 | 85 | 602 | 87 |
|  | Master Degree | 60 | 12 | 73 | 11 |
|  | Doctorate Degree | 15 | 03 | 13 | 2 |
|  |  |  |  |  |  |

Table S3. Words that have the most impact on the model's decision in Study 1

| **İndex** | Word | Control Group | Depression Group | Anxiety Group | Depressive-Anxiety Group | Global SHAP Value |
| --- | --- | --- | --- | --- | --- | --- |
|  | belirsiz (uncertain) | 0.572 | 0.865 | 0.573 | 0.637 | 0.662 |
|  | karamsar (pessimistic) | 0.388 | 0.690 | 0.349 | 0.790 | 0.554 |
|  | karanlık (darkness, as in mood) | 0.331 | 0.725 | 0.288 | 0.808 | 0.538 |
|  | kaygı (anxiety) | 0.376 | 0.598 | 0.389 | 0.760 | 0.531 |
|  | samimiyetsiz (insincere) | 0.333 | 0.494 | 0.529 | 0.710 | 0.517 |
|  | vermiyor (withholding, as in emotions or information) | 0.428 | 0.581 | 0.418 | 0.593 | 0.505 |
|  | belirsizlik (uncertainty) | 0.428 | 0.567 | 0.313 | 0.479 | 0.447 |
|  | umutsuz (hopeless) | 0.289 | 0.578 | 0.250 | 0.659 | 0.444 |
|  | umutsuzluk (hopelessness) | 0.316 | 0.448 | 0.358 | 0.488 | 0.403 |
|  | sıkıcı (tedious) | 0.277 | 0.538 | 0.263 | 0.532 | 0.403 |
|  | yalnızlık (loneliness) | 0.308 | 0.449 | 0.304 | 0.525 | 0.396 |
|  | depresif (depressed) | 0.281 | 0.437 | 0.253 | 0.589 | 0.390 |
|  | dengesiz (unstable) | 0.290 | 0.440 | 0.301 | 0.488 | 0.380 |
|  | berbat (awful) | 0.276 | 0.461 | 0.259 | 0.470 | 0.367 |
|  | kaygılı (anxious) | 0.324 | 0.399 | 0.299 | 0.440 | 0.365 |
|  | eğlenceli (enjoyable) | 0.260 | 0.391 | 0.320 | 0.424 | 0.349 |
|  | kararsız (indecisive) | 0.313 | 0.362 | 0.305 | 0.401 | 0.345 |
|  | anlamsız (meaningless) | 0.244 | 0.421 | 0.203 | 0.493 | 0.340 |
|  | yetersiz (inadequate) | 0.268 | 0.407 | 0.298 | 0.387 | 0.340 |
|  | mutsuz (unhappy) | 0.230 | 0.387 | 0.245 | 0.468 | 0.333 |
|  | yorucu (exhausting) | 0.239 | 0.384 | 0.255 | 0.452 | 0.332 |
|  | yoruldum (I'm tired) | 0.233 | 0.425 | 0.197 | 0.434 | 0.322 |
|  | bozuk (dispiritedness/ dysfunctional) | 0.222 | 0.388 | 0.242 | 0.428 | 0.320 |
|  | yorgunum (I'm exhausted) | 0.295 | 0.361 | 0.248 | 0.374 | 0.319 |
|  | huzursuz (restless / uneasy) | 0.283 | 0.376 | 0.224 | 0.389 | 0.318 |
|  | ilgisiz (indifferent) | 0.255 | 0.346 | 0.263 | 0.376 | 0.310 |
|  | korku (fear) | 0.210 | 0.357 | 0.211 | 0.454 | 0.308 |
|  | uzak (distant) | 0.253 | 0.366 | 0.220 | 0.385 | 0.306 |
|  | duygusuz (emotionless) | 0.221 | 0.317 | 0.230 | 0.456 | 0.306 |
|  | *-ilmez (-unable, -inable, cannot be)* | 0.207 | 0.326 | 0.297 | 0.392 | 0.306 |
|  | gereksiz(unnecessary) | 0.248 | 0.368 | 0.221 | 0.362 | 0.300 |
|  | zorlanıyorum (I'm struggling) | 0.194 | 0.345 | 0.184 | 0.426 | 0.287 |
|  | aciz (helpless) | 0.243 | 0.328 | 0.205 | 0.355 | 0.283 |
|  | korkutucu (frightening) | 0.134 | 0.337 | 0.224 | 0.435 | 0.282 |
|  | acımasız (cruel) | 0.209 | 0.306 | 0.199 | 0.400 | 0.279 |
|  | keyifli (pleasurable) | 0.257 | 0.334 | 0.208 | 0.311 | 0.277 |
|  | gergin (nervous) | 0.234 | 0.338 | 0.180 | 0.356 | 0.277 |
|  | yorgun (tired) | 0.233 | 0.303 | 0.226 | 0.340 | 0.276 |
|  | mutluyum (I am happy) | 0.218 | 0.329 | 0.228 | 0.300 | 0.269 |
|  | *-sizlik ("-lessness" veya "-less")* | 0.241 | 0.296 | 0.208 | 0.308 | 0.263 |
|  | salak (foolish) | 0.178 | 0.319 | 0.238 | 0.313 | 0.262 |
|  | aptal (stupid) | 0.184 | 0.310 | 0.185 | 0.365 | 0.261 |
|  | uyumak (to sleep) | 0.235 | 0.290 | 0.252 | 0.254 | 0.258 |
|  | tehlikeli (dangerous) | 0.248 | 0.314 | 0.186 | 0.280 | 0.257 |
|  | umursamaz (indifferent / unconcerned) | 0.188 | 0.301 | 0.203 | 0.334 | 0.257 |
|  | garip (odd / peculiar) | 0.218 | 0.321 | 0.239 | 0.245 | 0.256 |
|  | tedirgin (nervous / uneasy) | 0.213 | 0.280 | 0.193 | 0.336 | 0.256 |
|  | iyiyim (I'm well / fine) | 0.187 | 0.327 | 0.178 | 0.330 | 0.255 |
|  | tembel (lazy) | 0.221 | 0.309 | 0.191 | 0.274 | 0.249 |
|  | uyuyamıyorum (I can't sleep) | 0.143 | 0.292 | 0.190 | 0.356 | 0.245 |

Table S4. Words and SHAP values ​​that have the most impact on being included in the relevant class in Study 1

|  | Control Group | | Depression Group | | Anxiety Group | | Depressive-Anxiety Group | |
| --- | --- | --- | --- | --- | --- | --- | --- | --- |
| **İndex** | Word | SHAP Value | Word | SHAP Value | Word | SHAP Value | Word | SHAP Value |
|  | keyifli (pleasant) | 0.232 | belirsiz (uncertain) | 4.808 | heyecan (excitement) | 2.189 | karanlık (dark) | 0.800 |
|  | eğlenceli (fun) | 0.222 | vermiyor (withholding, as in emotions or information) | 2.135 | eğlenceli (fun) | 2.166 | karamsar (pessimistic) | 0.787 |
|  | mutluyum (I'm happy) | 0.173 | sapık (pervert) | 1.947 | tatlı (sweet) | 1.898 | kaygı (anxiety) | 0.683 |
|  | saygılı (respectful) | 0.156 | garip (weird / strange) | 1.727 | sabırlı (patient) | 1.764 | samimiyetsiz (insincere) | 0.680 |
|  | okurum (I read) | 0.155 | umutsuzluk (despair) | 1.613 | enerjik (energetic) | 1.602 | umutsuz (hopeless) | 0.645 |
|  | aktif (active) | 0.150 | gelmeyecek (will not come) | 1.470 | sınavı (the exam) | 1.481 | depresif (depressive) | 0.582 |
|  | huzurlu (peaceful) | 0.143 | yetersiz (insufficient) | 1.456 | doğum (birth) | 1.480 | belirsiz (uncertain) | 0.574 |
|  | gerekli (necessary) | 0.135 | sapıklık (perversion) | 1.439 | sevecen (affectionate) | 1.466 | vermiyor (withholding, as in emotions or information) | 0.570 |
|  | iyiyim (I'm well) | 0.134 | yoksun (deprived) | 1.304 | kızım (my daughter) | 1.451 | yalnızlık (loneliness) | 0.520 |
|  | severim (I love / I like) | 0.133 | ilkel (primitive) | 1.279 | kibar (polite) | 1.429 | sıkıcı (boring) | 0.508 |
|  | berrak (clear) | 0.132 | düşük (low) | 1.238 | heyecanlı (excited) | 1.345 | anlamsız (meaningless) | 0.490 |
|  | memnunum (I'm pleased) | 0.128 | maalesef (unfortunately) | 1.228 | bazen (sometimes) | 1.344 | umutsuzluk (despair) | 0.486 |
|  | ilgilendirmez (it doesn't concern) | 0.123 | gelmiyor (is not coming) | 1.224 | disiplinli (disciplined) | 1.303 | duygusuz (emotionless) | 0.448 |
|  | uyumlu (compatible / harmonious) | 0.122 | ülke (country) | 1.208 | mutluyum (I'm happy) | 1.303 | berbat (terrible) | 0.443 |
|  | yerinde (appropriate / in place) | 0.117 | sıradan (ordinary) | 1.151 | yararlı (useful) | 1.283 | mutsuz (unhappy) | 0.440 |
|  | sağlam (solid / sturdy) | 0.115 | değilim (I am not) | 1.151 | uyumak (to sleep) | 1.282 | yoruldum (I'm tired) | 0.430 |
|  | dengeli (balanced) | 0.109 | stabil (stable) | 1.134 | psikolog (psychologist) | 1.228 | dengesiz (unstable) | 0.425 |
|  | güzel (beautiful) | 0.108 | edemey (cannot / unable to) | 1.133 | abim (my brother) | 1.227 | kaygılı (anxious) | 0.421 |
|  | yararlı (useful) | 0.108 | sevgisiz (loveless) | 1.110 | azimli (determined) | 1.218 | bozuk (broken / malfunctioning) | 0.411 |
|  | olmasın (shouldn't be) | 0.107 | belirsizlik (uncertainty) | 1.106 | uyuma (sleeping) | 1.156 | zorlanıyorum (I'm struggling) | 0.397 |
|  | ideal (ideal) | 0.103 | Türkiye (Turkey) | 1.079 | yanımda (by my side) | 1.124 | korkutucu (frightening) | 0.395 |
|  | tatlı (sweet) | 0.102 | mutluydum (I was happy) | 1.074 | harika (wonderful) | 1.113 | korku (fear) | 0.392 |
|  | yaratıcı (creative) | 0.100 | tembellik (laziness) | 1.063 | priz (socket / outlet) | 1.111 | belirsizlik (uncertainty) | 0.389 |
|  | bayılıyorum (I'm crazy about / I adore) | 0.100 | tehlikelidir (is dangerous) | 1.054 | pozitif (positive) | 1.107 | kararsız (indecisive) | 0.388 |
|  | olmalıdır (must be / should be) | 0.099 | geriyor (is stretching) | 1.012 | keyifli (pleasant) | 1.057 | yorucu (exhausting) | 0.387 |
|  | gereklidir (is necessary) | 0.098 | ortalama (average) | 1.011 | muhteşem (magnificent) | 1.055 | acımasız (cruel) | 0.377 |
|  | disiplinli (disciplined) | 0.096 | duymaz (deaf to / does not hear) | 1.000 | huzurlu (peaceful) | 1.047 | yetersiz (insufficient) | 0.373 |
|  | *mıyorum (I am not doing)* | 0.096 | *eh (well / mediocre*) | 0.996 | sınav (exam) | 1.046 | itici (repulsive) | 0.372 |
|  | heyecanlı (excited) | 0.095 | *miyor (is not ving "doing" for "yapmıyor")* | 0.945 | uyuyorum (I'm sleeping) | 1.024 | huzursuz (restless) | 0.369 |
|  | sabırlı (patient) | 0.094 | hatırlamıyorum (I do not remember) | 0.943 | bayılıyorum (I adore / I'm fainting) | 0.992 | sıkıldım (I'm bored / fed up) | 0.356 |
|  | olmamalı (shouldn't be) | 0.092 | uyandır (wake up) | 0.932 | uyuya (falling asleep) | 0.964 | ilgisiz (indifferent) | 0.355 |
|  | faydalı (beneficial) | 0.091 | yetmiyor (is not enough) | 0.923 | *abilirim (I can do, form of the verb "to be able")* | 0.963 | yorgunum (I'm exhausted) | 0.346 |
|  | biriyimdir (I am someone) | 0.090 | cahil (ignorant) | 0.902 | sinirlenir (gets angry) | 0.963 | aciz (helpless) | 0.334 |
|  | dinlerim (I listen) | 0.089 | *gelmeye (not going to come)* | 0.875 | ailem (my family) | 0.940 | *-ilmez (-unable, -inable, cannot be)* | 0.333 |
|  | *şük (grateful - "şükran")* | 0.089 | olmamak (to not be) | 0.862 | uyur (sleeps) | 0.907 | aptal (stupid) | 0.330 |
|  | iyi (good) | 0.089 | zevkli (enjoyable) | 0.858 | dersleri (the lessons) | 0.904 | uzak (distant) | 0.328 |
|  | sahibiyim (I own / I have) | 0.089 | vefasız (faithless / ungrateful) | 0.849 | ameliyat (surgery) | 0.894 | yalnızım (I'm alone) | 0.327 |
|  | temiz (clean) | 0.088 | galiba (apparently / it seems) | 0.842 | mıyorum (I am not verbing) | 0.846 | uyuyamıyorum (I can't sleep) | 0.327 |
|  | hareketli (lively / active) | 0.087 | istismar (abuse) | 0.834 | uykum (my sleep) | 0.845 | gergin (tense) | 0.316 |
|  | ilgilendirir (it concerns) | 0.086 | iyilik (kindness / good deed) | 0.823 | iyiyim (I'm well) | 0.837 | umursamaz (indifferent / neglectful) | 0.316 |
|  | *zorlan (struggle - the act of struggling)* | 0.086 | olamıyor (cannot be / is not able to be) | 0.822 | uyku (sleep) | 0.833 | gereksiz (unnecessary) | 0.308 |
|  | *-lıyım (I am + adjective, e.g., "happy" for "mutluyum" or I must do)* | 0.085 | yok (do not have/has/not present) | 0.815 | kendim (myself) | 0.800 | tedirgin (anxious / uneasy) | 0.307 |
|  | yaparım (I do / I make) | 0.085 | gereksizdir (is unnecessary) | 0.810 | *zorlan (struggle - form of the verb "to struggle")* | 0.796 | egoist (egotistical) | 0.288 |
|  | bırakmaz (doesn't give up / doesn't let go) | 0.085 | sanmıyorum (I do not think) | 0.807 | saygılı (respectful) | 0.793 | kapanık (introverted / closed off) | 0.287 |
|  | pozitif (positive) | 0.085 | akılsız (foolish) | 0.803 | zorlanma (struggle - the act of) | 0.791 | sıkıntılı (troublesome) | 0.287 |
|  | önemli (important) | 0.085 | *madım (was not ving)* | 0.794 | bırakmaz (does not leave / does not give up) | 0.776 | bulanık (blurry / murky) | 0.286 |
|  | iyidir (is good) | 0.085 | travmatik (traumatic) | 0.779 | annem (my mother) | 0.742 | parasız (without money) | 0.284 |
|  | yolunda (on track / going well) | 0.084 | herhalde (probably) | 0.778 | zevkli (enjoyable) | 0.727 | salak (idiotic) | 0.278 |
|  | sevimli (cute) | 0.083 | bilgisiz (uninformed / unknowledgeable) | 0.778 | severim (I love / like) | 0.725 | duyarsız (insensitive) | 0.269 |
|  | uyuyorum (I'm sleeping) | 0.083 | aptalca  (silly) | 0.755 | dedem (my grandfather) | 0.725 | yapmacık (pretentious / artificial) | 0.268 |

Table S5. Words and SHAP values ​​that have the most impact on not being included in the relevant class in Study 1

|  | Control Group | | Depression Group | | Anxiety Group | | Depressive-Anxiety Group | |
| --- | --- | --- | --- | --- | --- | --- | --- | --- |
| **İndexs** | Word | SHAP Value | Word | SHAP Value | Word | SHAP Value | Word | SHAP Value |
|  | egoist (egotistic) | -0.162 | alıngan (touchy / sensitive) | -0.958 | sallantı (instability) | -1.110 | ideal (ideal) | -0.106 |
|  | vefasız (unfaithful / disloyal) | -0.163 | uyku (sleep) | -0.970 | adaletsizlik (injustice) | -1.119 | okurum (I read) | -0.107 |
|  | salak (idiot) | -0.163 | *-abilirim (I can do)* | -0.973 | sahtekar (fraudster / imposter) | -1.125 | bayılıyorum (I adore / I'm crazy about) | -0.108 |
|  | aptal (stupid) | -0.166 | tedirginlik (anxiety / unease) | -0.992 | geriyor (is stretching / straining) | -1.129 | sabırlı (patient) | -0.110 |
|  | tembel (lazy) | -0.170 | *uyuya (by sleeping / falling asleep)* | -0.994 | aciz (helpless) | -1.138 | iyidir (is good) | -0.111 |
|  | zorlanıyorum (I'm struggling) | -0.173 | enerjik (energetic) | -0.996 | adaletsiz (unjust) | -1.140 | olmasın (shouldn't be) | -0.111 |
|  | kibirli (arrogant) | -0.176 | sinirlenir (gets angry) | -1.003 | umursamaz (indifferent) | -1.141 | normal (normal) | -0.111 |
|  | umursamaz (careless / indifferent) | -0.176 | sabır (patience) | -1.012 | kaygılı (anxious) | -1.148 | temiz (clean) | -0.112 |
|  | *-ilmez* *(-unable,-inable, cannot be)* | -0.179 | doğum (birth) | -1.014 | sıkıldım (I am bored) | -1.158 | dinlerim (I listen) | -0.113 |
|  | parasız (broke / penniless) | -0.182 | korkutucu (scary / frightening) | -1.024 | yapmacık (fake / artificial) | -1.169 | yolunda (on track / going well) | -0.116 |
|  | sapık (pervert) | -0.185 | huzursuz (restless) | -1.032 | akılsız (mindless / foolish) | -1.188 | komik (funny) | -0.118 |
|  | garip (weird / odd) | -0.188 | yararlı (useful) | -1.045 | duyarsız (insensitive) | -1.190 | iyiydi (was good) | -0.120 |
|  | acımasız (cruel / ruthless) | -0.192 | okurum (I read) | -1.066 | gereksiz (unnecessary) | -1.208 | *zorlan (struggle - as in experiencing difficulty)* | -0.120 |
|  | yorgun (tired) | -0.194 | stres (stress) | -1.070 | yorucu (exhausting) | -1.218 | saygılı (respectful) | -0.121 |
|  | duyarsız (insensitive) | -0.195 | uyur (sleeps) | -1.085 | düşük (low) | -1.276 | doğal (natural) | -0.121 |
|  | gergin (tense / on edge) | -0.196 | azimli (determined) | -1.123 | ilgisiz (uninterested) | -1.283 | pozitif (positive) | -0.121 |
|  | yoksun (deprived / lacking) | -0.202 | psikolog (psychologist) | -1.132 | gelmeyecek (will not come) | -1.295 | *şük ("şükür" which means "gratitude" or "thanks")* | -0.122 |
|  | tedirgin (anxious / uneasy) | -0.204 | sıkıntı (trouble / distress) | -1.135 | etmiyor (is not doing) | -1.310 | normaldir (is normal) | -0.124 |
|  | mutsuz (unhappy) | -0.205 | saygılı (respectful) | -1.144 | bulanık (blurry) | -1.311 | enerjik (energetic) | -0.125 |
|  | bozuk (dispiritedness/ dysfunctional) | -0.209 | ezik (crushed / oppressed) | -1.156 | uzak (far) | -1.338 | tatlı (sweet) | -0.126 |
|  | duygusuz (unfeeling / emotionless) | -0.215 | acımasız (merciless) | -1.163 | belirsizlik (uncertainty) | -1.391 | iyilik (goodness / good deed) | -0.127 |
|  | yorucu (exhausting) | -0.216 | bıktım (I'm fed up) | -1.167 | depresif (depressive) | -1.438 | gezmek (to travel / to stroll) | -0.128 |
|  | sizlik (nonexistence / void) | -0.218 | sıkıntılı (troublesome) | -1.170 | mutsuz (unhappy) | -1.487 | yerinde (in place / appropriate) | -0.129 |
|  | uzak (distant / far) | -0.219 | kibar (polite) | -1.176 | duygusuz (emotionless) | -1.502 | gereklidir (is necessary) | -0.129 |
|  | gereksiz (unnecessary) | -0.221 | pişman (regretful) | -1.178 | yoksunu (devoid of) | -1.512 | mükemmel (perfect) | -0.131 |
|  | yoruldum (I'm tired) | -0.225 | sevecen (affectionate) | -1.194 | *miyor (suffix used to indicate negative continuous tense; "is not doing")* | -1.569 | yararlı (useful) | -0.132 |
|  | aciz (helpless) | -0.230 | ailem (my family) | -1.217 | itici (repulsive) | -1.611 | verimli (efficient / productive) | -0.132 |
|  | tehlikeli (dangerous) | -0.232 | şiddet (violence) | -1.223 | kararsız (indecisive) | -1.626 | stabil (stable) | -0.133 |
|  | ilgisiz (uninterested / indifferent) | -0.237 | sevgilim (my lover / my darling) | -1.235 | garip (strange) | -1.651 | rahatım (I'm comfortable) | -0.139 |
|  | anlamsız (meaningless) | -0.237 | zorlanıyorum (I'm struggling) | -1.240 | korkutucu (scary) | -1.660 | ilginç (interesting) | -0.140 |
|  | huzursuz (restless) | -0.246 | yanımda (by my side) | -1.258 | yoksun (deprived) | -1.703 | yapmaz (does not do) | -0.141 |
|  | sevgisiz (loveless) | -0.247 | kırgın (resentful / hurt) | -1.288 | tutarsız (inconsistent) | -1.742 | sağlam (solid / sturdy) | -0.143 |
|  | sıkıcı (boring) | -0.251 | karamsar (pessimistic) | -1.336 | anlamsız (meaningless) | -1.758 | harika (wonderful) | -0.144 |
|  | yetersiz (inadequate) | -0.255 | kızım (my daughter) | -1.380 | berbat (awful) | -1.763 | severim (I love / I like) | -0.147 |
|  | yorgunum (I'm exhausted) | -0.267 | uykum (my sleep) | -1.402 | yoruldum (I am tired) | -1.781 | iyi (good) | -0.151 |
|  | depresif (depressive) | -0.269 | abim (my brother) | -1.419 | salak (stupid) | -1.801 | güzel (beautiful) | -0.152 |
|  | dengesiz (unstable) | -0.271 | gergin (tense / strained) | -1.427 | dengesiz (unstable) | -1.861 | aktif (active) | -0.159 |
|  | umutsuz (hopeless) | -0.273 | itici (repulsive / off-putting) | -1.444 | bozuk (dispiritedne) | -1.972 | uyumlu (compatible / harmonious) | -0.159 |
|  | berbat (awful) | -0.273 | uyuma (sleeping - the act of) | -1.451 | umutsuz (hopeless) | -1.999 | disiplinli (disciplined) | -0.164 |
|  | yalnızlık (loneliness) | -0.293 | hataları (mistakes) | -1.463 | sıkıcı (boring) | -2.075 | heyecanlı (excited) | -0.165 |
|  | samimiyetsiz (insincere) | -0.296 | uyuyamıyorum (I can't sleep) | -1.493 | karanlık (darkness) | -2.095 | *mıyorum (the negation suffix for continuous tense in Turkish; "I am not Ving")* | *-0.167* |
|  | kaygı (anxiety) | -0.299 | sabırlı (patient) | -1.607 | -*ilmez ((-unable, -inable, cannot be))* | -2.123 | ilgilendirmez (it does not concern / not of interest) | -0.168 |
|  | kararsız (indecisive) | -0.300 | sıkıldım (I'm bored) | -1.649 | yalnızlık (loneliness) | -2.333 | gerekli (necessary) | -0.184 |
|  | kaygılı (anxious) | -0.305 | tatlı (sweet) | -1.660 | yetersiz (insufficient) | -2.633 | huzurlu (peaceful) | -0.200 |
|  | umutsuzluk (despair) | -0.316 | depresif (depressive) | -1.690 | karamsar (pessimistic) | -2.678 | heyecan (excitement) | -0.201 |
|  | karanlık (darkness) | -0.323 | korku (fear) | -1.713 | kaygı (worry) | -2.923 | zevkli (pleasurable / enjoyable) | -0.205 |
|  | belirsizlik (uncertainty) | -0.361 | umutsuz (hopeless) | -1.721 | umutsuzluk (despair) | -3.320 | mutluyum (I'm happy) | -0.256 |
|  | karamsar (pessimistic) | -0.385 | uyumak (to sleep) | -1.740 | vermiyor (withholding, as in emotions or information) | -3.635 | iyiyim (I'm well / I'm fine) | -0.283 |
|  | vermiyor (withholding, as in emotions or information) | -0.420 | uykusuz (sleepless / insomniac) | -1.900 | samimiyetsiz (insincere) | -4.157 | keyifli (pleasant) | -0.297 |
|  | belirsiz (uncertain) | -0.549 | karanlık (darkness / dark) | -2.669 | belirsiz (uncertain) | -5.056 | eğlenceli (fun) | -0.399 |

Table S6. The first 50 words that most predict pathology groups in HO_Cont_vs_Self_Pat_Dia_Test, Self_Past_vs_Pat_Dia_Test sets, with Subclinical Group in the Internal test in Study 2

|  | Internal Test | HO_Cont_vs_Self_Pat_Dia_Test | | | Self_Past_vs_Pat_Dia_Test | |
| --- | --- | --- | --- | --- | --- | --- |
|  | Subclinical Group | Self_Pat_Dia_G | | | Self_Pat_Dia_G | |
| **İndexs** | Words | SHAP Value | Words | SHAP Value | Words | SHAP Value |
|  | karamsar (pessimistic) | 5.893 | edemezler (they cannot do) | 12.664 | edemezler (they cannot do) | 12.664 |
|  | yoksun (deprived) | 5.876 | vicdansız (heartless) | 8.578 | acayip (weird) | 8.727 |
|  | korkuyorum (I am scared) | 5.821 | karamsar (pessimistic) | 8.089 | vurdumduymaz (indifferent) | 7.419 |
|  | sapık (pervert) | 5.622 | uyuyamıyorum (I cannot sleep) | 7.966 | vicdansız (heartless) | 7.291 |
|  | umutsuz (hopeless) | 5.537 | yoksunu (deprived) | 7.753 | kayıp (loss) | 7.284 |
|  | berbat (terrible) | 5.240 | yoruldu (got tired) | 7.051 | yoruldu (got tired) | 7.051 |
|  | samimiyetsiz (insincere) | 5.161 | umutsuz (hopeless) | 6.964 | psikopat (psychopath) | 7.037 |
|  | tutarsız (inconsistent) | 4.914 | verimsiz (unproductive) | 6.963 | beklenti (expectation) | 6.608 |
|  | mutsuz (unhappy) | 4.687 | toksik (toxic) | 6.905 | karamsar (pessimistic) | 6.560 |
|  | belirsiz (uncertain) | 4.597 | yapamayan (unable to do) | 6.793 | yordu (tired) | 6.398 |
|  | yoksunu (deprived of) | 4.560 | yordu (tired) | 6.764 | vermiyor (does not give) | 6.369 |
|  | zorlanıyorum (I am struggling) | 4.442 | problemi (problem) | 5.814 | umutsuz (hopeless) | 6.356 |
|  | ilkel (primitive) | 4.434 | karanlık (dark) | 5.575 | verimsiz (unproductive) | 6.227 |
|  | duygusuz (emotionless) | 4.219 | bilmeden (unknowingly) | 5.574 | azalıyor (is decreasing) | 6.053 |
|  | uyuyamıyorum (I cannot sleep) | 4.213 | korkak (coward) | 5.528 | kıran (breaker) | 5.959 |
|  | anksiyete (anxiety) | 4.176 | bıkkın (weary) | 5.524 | fakir (poor) | 5.955 |
|  | depresif (depressive) | 3.843 | alıyor (takes) | 5.356 | uzaktır (is distant) | 5.862 |
|  | aciz (helpless) | 3.767 | kahrol (be devastated) | 5.226 | karanlık (dark) | 5.802 |
|  | vermiyor (does not give) | 3.764 | duygusuz (emotionless) | 5.205 | kibirli (arrogant) | 5.725 |
|  | karanlık (dark) | 3.722 | kırgın (hurt) | 5.100 | belası (curse) | 5.606 |
|  | aptal (stupid) | 3.645 | tedirgin (uneasy) | 4.977 | toksik (toxic) | 5.600 |
|  | yorgunum (I am tired) | 3.610 | depresif (depressive) | 4.884 | yedim (I ate) | 5.551 |
|  | bozuk (broken) | 3.494 | anlayamaz (cannot understand) | 4.823 | unut (forget) | 5.532 |
|  | bitkin (exhausted) | 3.488 | ihanetin (betrayal’s) | 4.822 | boku (crap) | 5.279 |
|  | kaygı (worry) | 3.476 | lanetli (cursed) | 4.750 | azalacak (will decrease) | 5.049 |
|  | tatmin (satisfaction) | 3.476 | samimiyetsiz (insincere) | 4.646 | anlayamaz (cannot understand) | 4.823 |
|  | sapıklık (perversion) | 3.347 | atalet (inertia) | 4.486 | yoruyor (it tires) | 4.777 |
|  | sorunlu (problematic) | 3.301 | *edemey (cannot do*) | 4.430 | korkak (coward) | 4.753 |
|  | yorgun (tired) | 3.223 | kaderim (my destiny) | 4.425 | kaderim (my destiny) | 4.425 |
|  | tedirgin (uneasy) | 3.180 | kesmeye (to cut) | 4.335 | duygusuz (emotionless) | 4.372 |
|  | egoist (selfish) | 3.173 | bensiz (without me) | 4.248 | bitecek (will end) | 4.369 |
|  | garip (weird) | 3.128 | sahte (fake) | 4.067 | kesmeye (to cut) | 4.335 |
|  | kör (blind) | 3.126 | kapanık (introverted) | 3.951 | tedirgin (uneasy) | 4.249 |
|  | adaletsiz (unfair) | 3.080 | tehditler (threats) | 3.948 | yapamam (I cannot do) | 4.046 |
|  | bozuluyor (it breaks) | 3.076 | bahtsız (unfortunate) | 3.937 | kompleks (complex) | 3.962 |
|  | kırgın (hurt) | 3.062 | tatmin (satisfaction) | 3.852 | tehditler (threats) | 3.948 |
|  | huzursuz (restless) | 3.054 | ihtiyar (elderly) | 3.767 | midem (my stomach) | 3.861 |
|  | bilmeden (unknowingly) | 3.053 | kapanır (closes) | 3.737 | *devir (era)* | 3.859 |
|  | vasat (mediocre) | 3.048 | *çen (jaw)* | 3.714 | kararsız (indecisive) | 3.850 |
|  | *Sizlik (not, non-existent, , -lessness))* | 3.020 | korkuyorum (I am scared) | 3.699 | rezil (disgraceful) | 3.772 |
|  | korku (fear) | 3.009 | belirsiz (uncertain) | 3.698 | *çen (jaw)* | 3.714 |
|  | yetersiz (insufficient) | 2.994 | yaramaz (naughty) | 3.619 | yapamayan (unable to do) | 3.551 |
|  | *edemey (cannot do)* | 2.971 | bozulmaya (to deteriorate) | 3.592 | *evham (paranoia)* | 3.540 |
|  | yapmacık (fake) | 2.968 | bihaber (unaware) | 3.534 | bihaber (unaware) | 3.534 |
|  | umutsuzluk (hopelessness) | 2.961 | karakter (character) | 3.515 | unutmak (to forget) | 3.533 |
|  | yalnız (lonely) | 2.952 | zorlanıyorum (I am struggling) | 3.471 | *vah (alas)* | 3.477 |
|  | vefasız (ungrateful) | 2.947 | yoruyor (it tires) | 3.466 | kapanır (closes) | 3.471 |
|  | sıkıcı (boring) | 2.933 | bozuluyor (it breaks) | 3.446 | *boz (break)* | 3.465 |
|  | belirsizlik (uncertainty) | 2.849 | sıkıntılı (troubled) | 3.415 | belirsizlik (uncertainty) | 3.440 |
|  | yıpran (worn out) | 2.838 | kör (blind) | 3.395 | kazanma (winning) | 3.436 |

Table S7. The first 50 words that most predict control groups in the Internal test, HO_Cont_vs_Self_Pat_Dia_Test, Self_Past_vs_Pat_Dia_Test sets.

|  | Internal Test | HO_Cont_vs_Self_Pat_Dia_Test | | | Self_Past_vs_Pat_Dia_Test | |
| --- | --- | --- | --- | --- | --- | --- |
|  | Internal Control Group | HO_Cont_G | | | Self_Past_Dia_G | |
| **İndex** | Words | SHAP Value | Words | SHAP Value | Words | SHAP Value |
|  | keyifli (enjoyable) | 4.657 | yoktu (was not) | 5.675 | zevkli (pleasant) | 7.082 |
|  | eğlenceli (fun) | 4.345 | geniş (wide) | 5.339 | tatili (vacation) | 5.259 |
|  | olmasın (let it not be) | 3.853 | aktif (active) | 5.038 | mutlulukla (with happiness) | 4.894 |
|  | aktif (active) | 3.621 | mutlulukla (with happiness) | 4.894 | yoktu (was not) | 4.702 |
|  | bırakmaz (does not leave) | 3.276 | aktiviteler (activities) | 4.440 | keyifli (enjoyable) | 4.478 |
|  | korkutma (do not scare) | 3.174 | keyifli (enjoyable) | 3.943 | geniş (wide) | 4.414 |
|  | geniş (wide) | 2.921 | uyarır (warns) | 3.837 | eğlenceli (fun) | 4.088 |
|  | ilgilendirmez (does not concern) | 2.897 | ilkeli (principled) | 3.682 | hareketli (lively) | 4.033 |
|  | aktiviteler (activities) | 2.663 | liydi (was with) | 3.651 | rahatlık (comfort) | 4.025 |
|  | olmaması (not being) | 2.636 | geldim (I came) | 3.465 | kapitalizmi (capitalism) | 3.947 |
|  | severim (I like) | 2.495 | eğlenceli (fun) | 3.406 | uyumlu (compatible) | 3.839 |
|  | enerjik (energetic) | 2.456 | takıl (hang out) | 3.272 | uyarır (warns) | 3.837 |
|  | tatlı (sweet) | 2.444 | suda (in water) | 2.970 | bırakmaz (does not leave) | 3.819 |
|  | yapmaz (does not do) | 2.390 | olmazıdır (it should not be) | 2.937 | kurar (sets up) | 3.800 |
|  | bazen (sometimes) | 2.388 | tabi (of course) | 2.931 | yardımsever (helpful) | 3.738 |
|  | pozitif (positive) | 2.315 | kazandır (make gain) | 2.927 | disiplinli (disciplined) | 3.716 |
|  | dinlerim (I listen) | 2.263 | duyarlı (sensitive) | 2.802 | yapmadım (I did not do) | 3.707 |
|  | iyidir (it is good) | 2.219 | hafif (light) | 2.800 | ilgilenirim (I am interested) | 3.319 |
|  | normaldir (it is normal) | 2.159 | *lidir (is with)* | 2.719 | takıl (hang out) | 3.272 |
|  | tercihim (my preference) | 2.090 | tanır (recognizes) | 2.698 | yetenekli (talented) | 3.214 |
|  | *eh (meh)* | 2.081 | moral (morale) | 2.673 | mutludur (is happy) | 3.157 |
|  | *şahan (magnificent*) | 2.075 | yetenekli (talented) | 2.620 | dengeli (balanced) | 3.120 |
|  | destekler (supports) | 2.047 | takılır (hangs out) | 2.619 | pozitif (positive) | 3.046 |
|  | doğum (birth) | 2.039 | vericiydi (was giving) | 2.612 | detay (detail) | 3.016 |
|  | olmamak (not being) | 2.033 | ilgilendirmez (does not concern) | 2.590 | *suda (in water)* | 2.970 |
|  | genelde (generally) | 2.032 | farkındalık (awareness) | 2.586 | olmazıdır (it should not be) | 2.937 |
|  | gezmek (to wander) | 2.026 | serbest (free) | 2.556 | hormonlar (hormones) | 2.880 |
|  | Dedem (my grandfather) | 2.016 | etmeliyim (I should do) | 2.549 | entel (intellectual) | 2.863 |
|  | olumlu (positive) | 2.007 | mecburi (mandatory) | 2.514 | moral (morale) | 2.673 |
|  | güzel (beautiful) | 1.998 | erkeklerin (of men) | 2.513 | getirmesini (to bring) | 2.662 |
|  | verimli (productive) | 1.993 | yapmadım (I did not do) | 2.507 | sevinirim (I would be happy) | 2.660 |
|  | okurum (I read) | 1.976 | istemez (does not want) | 2.445 | takılır (hangs out) | 2.619 |
|  | iyiyim (I am good) | 1.963 | nispeten (relatively) | 2.439 | vericiydi (was giving) | 2.612 |
|  | süper (super) | 1.911 | malum (obvious) | 2.438 | spor (sport) | 2.583 |
|  | bayılıyorum (I love it) | 1.908 | evcil (domestic) | 2.428 | severim (I like) | 2.576 |
|  | bakarım (I look) | 1.886 | muhteşem (magnificent) | 2.387 | *gez (wander)* | 2.571 |
|  | bırakmadı (did not leave) | 1.884 | beslenir (is fed) | 2.342 | dokunmaz (does not touch) | 2.567 |
|  | ideal (ideal) | 1.878 | samimiyetle (sincerely) | 2.328 | serbest (free) | 2.556 |
|  | tabi (of course) | 1.869 | verebilmek (to be able to give) | 2.318 | orantılı (proportional) | 2.554 |
|  | sevecen (loving) | 1.843 | etmez (does not do) | 2.313 | sarılır (hugs) | 2.461 |
|  | hareketli (lively) | 1.825 | sayılır (is considered) | 2.306 | sakinlik (calmness) | 2.460 |
|  | gerekli (necessary) | 1.803 | yardımsever (helpful) | 2.299 | inşallah (hopefully) | 2.458 |
|  | zevkli (pleasant) | 1.802 | getiriliyor (is being brought) | 2.281 | trol (troll) | 2.433 |
|  | doğal (natural) | 1.795 | atlattı (overcame) | 2.278 | evcil (domestic) | 2.428 |
|  | etmezler (they do not do) | 1.779 | destekler (supports) | 2.260 | olsunlar (let them be) | 2.408 |
|  | *mıyorum (I am not)* | 1.749 | azimle (with determination) | 2.251 | *memeli (shouldn't*  *V1)* | 2.387 |
|  | muhteşem (magnificent) | 1.748 | eksiksiz (complete) | 2.235 | keyifle (with pleasure) | 2.364 |
|  | yardımsever (helpful) | 1.734 | keyifle (with pleasure) | 2.229 | saat (hour) | 2.358 |
|  | *liydi (was with)* | 1.716 | bırakmaz (does not leave) | 2.207 | beslenir (is fed) | 2.342 |
|  | saygılı (respectful) | 1.698 | mesajı (the message) | 2.206 | samimiyetle (sincerely) | 2.328 |

Table S8. This table shows average model performance metrics for BERT and SVM in Study 1.

|  | Study 1 | | | | | | | | | | | |
| --- | --- | --- | --- | --- | --- | --- | --- | --- | --- | --- | --- | --- |
| Internal Test | BERT | | | | | | SVM | | | | | |
|  | Precision | | Recall | | F1 | | Precision | | Recall | | F1 | |
|  | Macro | Weighted | Macro | Weighted | Macro | Weighted | Macro | Weighted | Macro | Weighted | Macro | Weighted |
| Value | .412 | .498 | .413 | .469 | .398 | .471 | .443 | .543 | .444 | .479 | .429 | .498 |
| sd. | .08 | .09 | .06 | .05 | .07 | .07 | .04 | .04 | .05 | .05 | .04 | .05 |

Table S9. This table shows average model performance metrics for SVM in Study 1.

|  | SVM in Study1 | | | | | | | | | | | |
| --- | --- | --- | --- | --- | --- | --- | --- | --- | --- | --- | --- | --- |
| Internal Test | I_Talk_O_Count | | | | | | I_Talk_G_Count | | | | | |
|  | Precision | | Recall | | F1 | | Precision | | Recall | | F1 | |
|  | Macro | Weighted | Macro | Macro | Weighted | Macro | Macro | Weighted | Macro | Weighted | Macro | Weighted |
| Value | .443 | .543 | .444 | .479 | .429 | .498 | .433 | .537 | .429 | .474 | .418 | .493 |
| sd. | .04 | .04 | .05 | .05 | .04 | .05 | .03 | .04 | .03 | .04 | .03 | .04 |

Table S10. This table shows average model performance metrics for BERT and SVM in Study 2.

|  | Study 2 | | | | | |
| --- | --- | --- | --- | --- | --- | --- |
| Test Set | BERT | | | SVM | | |
|  | Precision | Recall | F1 | Precision | Recall | F1 |
| Internal Test | .710 | .712 | .702 | .720 | .717 | .715 |
| Hold-out Test | .706 | .713 | .703 | .692 | .692 | .692 |
| HO_Cont_vs_Self_Pat_Dia_Test | .736 | .741 | .729 | .748 | .749 | .745 |
| HO_Cont_vs_Self_Past_Dia_Test | .512 | .523 | .506 | .540 | .543 | .539 |
| Self_Past_vs_Pat_Dia_Test | .725 | .732 | .720 | .720 | .724 | .717 |

Table S11. This table presents a comparison of the results obtained in the SVM analysis when I_Talk_O_Count was removed and the variable I_Talk_G_Count was used instead in Study 2

|  | **I_Talk_O_Count** | | | **I_Talk_G_Count** | | |
| --- | --- | --- | --- | --- | --- | --- |
| **Test Name** | **Precision** | **Recall** | **F1** | **Precision** | **Recall** | **F1** |
| Internal Test | .720 | .717 | .715 | .720 | .717 | .716 |
| Hold-out Test | .692 | .692 | .692 | .696 | .696 | .696 |
| HO_Cont_vs_Self_Pat_Dia_Test | .748 | .749 | .745 | .747 | .747 | .744 |
| HO_Cont_vs_Self_Past_Dia_Test | .540 | .543 | .539 | .533 | .536 | .532 |
| Self_Past_vs_Pat_Dia_Test | .720 | .724 | .717 | .724 | .728 | .722 |

Table S12. This table presents a comparison of the results obtained in the SVM analysis when I_Talk_O_Count was removed and the variable I_Talk_G_Count was used instead in Study 2

| **Test Name** | **I_Talk_O_Count** | | **I_Talk_G_Count** | |
| --- | --- | --- | --- | --- |
|  | *AUC* | *sd* | *AUC* | *sd* |
| Internal Test | .78 | .05 | .78 | .05 |
| Hold-out Test | .78 | .01 | .79 | .01 |
| HO_Cont_vs_Self_Pat_Dia_Test | .84 | .02 | .85 | .02 |
| HO_Cont_vs_Self_Past_Dia_Test | .56 | .01 | .57 | .02 |
| Self_Past_vs_Pat_Dia_Test | .81 | .02 | .82 | .01 |

**Table S13**. Shows GWET AC1 values ​​calculated after referee scoring.

| **Sentences No** | **Agreement** | **GWET AC1** | **Confidence Interval %95** | ***p*** |
| --- | --- | --- | --- | --- |
| 52* | 0.32 | 0.01 | [0.051,0.068] | 0.77 |
| 26* | 0.37 | 0.06 | [0.032,0.089] | 0.00 |
| 17* | 0.39 | 0.21 | [0.19,0.238] | 0.00 |
| 18 | 0.53 | 0.41 | [0.375,0.452] | 0.00 |
| 50 | 0.58 | 0.44 | [0.401,0.481] | 0.00 |
| 41 | 0.58 | 0.41 | [0.351,0.456] | 0.00 |
| 23 | 0.59 | 0.42 | [0.379,0.461] | 0.00 |
| 45 | 0.59 | 0.43 | [0.371,0.483] | 0.00 |
| 44 | 0.62 | 0.44 | [0.396,0.485] | 0.00 |
| 48 | 0.63 | 0.52 | [0.476,0.56] | 0.00 |
| 65 | 0.68 | 0.53 | [0.482,0.576] | 0.00 |
| 56 | 0.69 | 0.57 | [0.518,0.622] | 0.00 |
| 19 | 0.70 | 0.61 | [0.57,0.657] | 0.00 |
| 2 | 0.70 | 0.56 | [0.518,0.61] | 0.00 |
| 57 | 0.70 | 0.56 | [0.512,0.603] | 0.00 |
| 14 | 0.71 | 0.58 | [0.539,0.631] | 0.00 |
| 55 | 0.72 | 0.58 | [0.532,0.623] | 0.00 |
| 60** | 0.73 | 0.59 | [0.547,0.634] | 0.00 |
| 36 | 0.73 | 0.66 | [0.608,0.705] | 0.00 |
| 9 | 0.74 | 0.61 | [0.563,0.651] | 0.00 |
| 28 | 0.74 | 0.61 | [0.567,0.658] | 0.00 |
| 25 | 0.74 | 0.62 | [0.58,0.664] | 0.00 |
| 42 | 0.75 | 0.63 | [0.586,0.68] | 0.00 |
| 11 | 0.76 | 0.64 | [0.596,0.682] | 0.00 |
| 4** | 0.76 | 0.64 | [0.596,0.682] | 0.00 |
| 39 | 0.76 | 0.66 | [0.62,0.701] | 0.00 |
| 59 | 0.76 | 0.64 | [0.6,0.687] | 0.00 |
| 13 | 0.76 | 0.66 | [0.614,0.7] | 0.00 |
| 24 | 0.77 | 0.73 | [0.692,0.766] | 0.00 |
| 29 | 0.77 | 0.69 | [0.642,0.729] | 0.00 |
| 67 | 0.78 | 0.67 | [0.629,0.715] | 0.00 |
| 12 | 0.78 | 0.69 | [0.648,0.73] | 0.00 |
| 40 | 0.78 | 0.67 | [0.628,0.713] | 0.00 |
| 49 | 0.78 | 0.67 | [0.626,0.714] | 0.00 |
| 21 | 0.79 | 0.69 | [0.648,0.731] | 0.00 |
| 46 | 0.79 | 0.69 | [0.644,0.728] | 0.00 |
| 61 | 0.80 | 0.70 | [0.658,0.745] | 0.00 |
| 63 | 0.80 | 0.71 | [0.673,0.757] | 0.00 |
| 31 | 0.80 | 0.74 | [0.703,0.781] | 0.00 |
| 10 | 0.80 | 0.71 | [0.668,0.75] | 0.00 |
| 34 | 0.81 | 0.72 | [0.683,0.764] | 0.00 |
| 58 | 0.81 | 0.74 | [0.695,0.778] | 0.00 |
| 53 | 0.81 | 0.77 | [0.732,0.808] | 0.00 |
| 20 | 0.81 | 0.78 | [0.744,0.814] | 0.00 |
| 16 | 0.82 | 0.74 | [0.703,0.779] | 0.00 |
| 8 | 0.82 | 0.73 | [0.689,0.769] | 0.00 |
| 15 | 0.82 | 0.73 | [0.695,0.775] | 0.00 |
| 6** | 0.82 | 0.74 | [0.697,0.774] | 0.00 |
| 62 | 0.82 | 0.74 | [0.698,0.78] | 0.00 |
| 7 | 0.82 | 0.74 | [0.699,0.778] | 0.00 |
| 30 | 0.83 | 0.80 | [0.771,0.839] | 0.00 |
| 27 | 0.83 | 0.76 | [0.724,0.8] | 0.00 |
| 47 | 0.83 | 0.75 | [0.714,0.792] | 0.00 |
| 33 | 0.83 | 0.82 | [0.788,0.848] | 0.00 |
| 66 | 0.83 | 0.80 | [0.761,0.832] | 0.00 |
| 51 | 0.83 | 0.75 | [0.715,0.792] | 0.00 |
| 64 | 0.84 | 0.82 | [0.788,0.851] | 0.00 |
| 35 | 0.84 | 0.81 | [0.779,0.848] | 0.00 |
| 5 | 0.85 | 0.83 | [0.798,0.86] | 0.00 |
| 54 | 0.86 | 0.84 | [0.807,0.867] | 0.00 |
| 38** | 0.86 | 0.80 | [0.761,0.835] | 0.00 |
| 32 | 0.87 | 0.85 | [0.821,0.88] | 0.00 |
| 22 | 0.88 | 0.83 | [0.797,0.864] | 0.00 |
| 37 | 0.89 | 0.88 | [0.856,0.906] | 0.00 |
| 1 | 0.91 | 0.86 | [0.835,0.895] | 0.00 |
| 3 | 0.91 | 0.90 | [0.871,0.92] | 0.00 |
| 43 | 0.93 | 0.92 | [0.9,0.944] | 0.00 |
| *NOTES. Questions are listed in ascending order according to AC1 Value.*  ** Since Gwet AC1 values ​​are low, they are not included in the SVM analysis.*  *** Since it was seen that the majority of the answers consisted of single-word answers such as 'none, private or I do not work', they were not included in both analaysis (BERT and SVM)* | | | | |

**Table S14.** Table of VIF values ​​for SVM analyses

| Feature | VIF | Tolerance Value |
| --- | --- | --- |
| Word_Count | 2.06 | .48 |
| I_Talk_O_Count * | 2.54 | .39 |
| I_Talk_G_Count | 3.45 | .29 |
| Depression_Word_Count | 1.43 | .70 |
| Anxiety_Word_Count | 1.19 | .84 |
| Negative_Past_Attitude | 1.80 | .56 |
| Negative_Future_Attitude | 3.58 | .28 |
| Negative_Opposite_Sex_Attitude | 1.48 | .67 |
| Negative_Maternal_Attitude | 2.38 | .41 |
| Negative_Paternal_Attitude | 2.14 | .47 |
| Negative_Home_Family_Attitude | 3.01 | .33 |
| Negative_Friend_Attitude | 2.43 | .41 |
| Negative_Authority_Attitude | 1.44 | .69 |
| Negative_Fear_Anxiety_Attitude | 2.80 | .36 |
| Negative_Guilt_Feeling | 1.44 | .69 |
| Negative_School_Work_Attitude | 1.76 | .57 |
| Negative_Self_Efficacy_Attitude | 2.78 | .36 |
| Negative_General_Attitudes | 2.40 | .41 |
| Positive_Past_Attitude | 1.73 | .58 |
| Positive_Future_Attitude | 3.50 | .28 |
| Positive_Opposite_Sex_Attitude | 1.37 | .73 |
| Positive_Maternal_Attitude | 2.44 | .41 |
| Positive_Paternal_Attitude | 2.14 | .47 |
| Positive_Home_Family_Attitude | 3.01 | .33 |
| Positive_Friend_Attitude | 2.39 | .41 |
| Positive_Authority_Attitude | 1.44 | .69 |
| Positive_Fear_Anxiety_Attitude | 3.00 | .33 |
| Positive_Guilt_Feeling | 1.39 | .72 |
| Positive_School_Work_Attitude | 1.61 | .62 |
| Positive_Self_Efficacy_Attitude | 2.70 | .37 |
| Positive_General_Attitudes | 2.25 | .44 |
| *Not*. * In Turkish, 'I' can be expressed explicitly with the pronoun 'ben' (e.g., Ben depresyondayım [I am depressed]) or implicitly through grammatical structures like verb conjugations and possessive suffixes (e.g., Depresyondayım [I am depressed]). Accordingly, SVM analyses were performed in two formats: one for explicit 'I' statements (I_Talk_O_Count) and another encompassing both implicit and explicit forms. | | |

**Table S15.** External validation created for Study 2 shows multiple comparisons to show that the test sets had the same demographic characteristics, word count, and emotion as the training set.

|  | | **Train Data** | | | | | | | | | | | | | |
| --- | --- | --- | --- | --- | --- | --- | --- | --- | --- | --- | --- | --- | --- | --- | --- |
|  |  | Gender | | Education Level | | Income  Level | | Age | | Word Count | | Panas_Negative | | Panas_Positive | |
|  |  | *χ²* | *p* | *χ²* | *p* | *χ²* | *p* | *χ²* | *p* | *U* | *p* | *U* | *p* | *U* | *p* |
| **For BERT** | Hold-Out Test | .43 | .76 | 1.34 | .76 | 1.04 | .90 | 2.35 | .76 | 117434 | .86 | 118724 | .76 | 110963 | .76 |
|  | HO_Cont_vs_Self_Pat_Dia_Test | .44 | .66 | 1.90 | .53 | 2.95 | .66 | 2.21 | .48 | 111311 | .57 | 116518.5 | .88 | 123769 | .48 |
|  | HO_Cont_vs_Self_Past_Dia_Test | .92 | .48 | .98 | .48 | 4.41 | .48 | .53 | .48 | 88501 | .48 | 131250 | .00* | 76061.5 | .00* |
|  | Self_Past_vs_Pat_Dia_Test | 1.41 | .41 | 1.49 | .77 | 4.90 | .42 | 2.31 | .30 | 102953 | .30 | 109350 | .76 | 118925 | .30 |
| **For SVM** | Hold-Out Test | .55 | .80 | 1.57 | .80 | .96 | .92 | 1.98 | .56 | 113829.5 | .92 | 103477.5 | .13 | 115253.5 | .92 |
|  | HO_Cont_vs_Self_Pat_Dia_Test | .07 | .79 | .81 | .74 | 6.07 | .34 | 1.69 | .34 | 110633 | .55 | 106538 | .30 | 123960.5 | .30 |
|  | HO_Cont_vs_Self_Past_Dia_Test | 1.04 | .43 | 3.43 | .15 | 1.79 | .86 | .03 | .86 | 86773 | .34 | 120173 | .00* | 75888 | .00* |
|  | Self_Past_vs_Pat_Dia_Test | .26 | .86 | .008 | .93 | .85 | .93 | .71 | .93 | 101342 | .28 | 105617 | .71 | 119472.5 | .22 |
| *NOTES. Each test set was compared with its own training set.*  ** Indicates significance after Benjamini-Hochberg correction (p < .001).* | | | | | | | | | | | | | | | |

Table S16. The external validation test sets created for Study 2 show their internal comparisons. In addition, each test set was compared within itself in terms of demographic characteristics and number of words, and the confounding effect of the number of words was checked.

|  | | **In-group^1^** | | | | | | | | | | **Word Count^2^ vs** | | | | | |
| --- | --- | --- | --- | --- | --- | --- | --- | --- | --- | --- | --- | --- | --- | --- | --- | --- | --- |
|  |  | Gender | | Education Level | | Income  Level | | Age | | Word Count | | Age | | Gender | | Education Level | |
|  |  | *χ²* | *p* | *χ²* | *p* | *χ²* | *p* | *χ²* | *p* | *U* | *p* | *U* | *p* | *U* | *p* | *U* | *p* |
| **For BERT** | Hold-Out Test | 3.84 | .28 | 2.34 | .34 | 5.32 | .28 | 5.84 | .58 | 6755.5 | .65 | 4584 | .78 | 6216 | .65 | 4584 | .61 |
|  | HO_Cont_vs_Self_Pat_Dia_Test | 9.32 | .02* | 2.93 | .34 | 1.03 | .78 | .62 | .78 | 7407 | .78 | 5799.5 | .48 | 6123 | .78 | 3379.5 | .92 |
|  | HO_Cont_vs_Self_Past_Dia_Test | .92 | .59 | .81 | .59 | 4.41 | .87 | .60 | .59 | 4331 | .59 | 3909 | .62 | 4394 | .62 | 2833.5 | .62 |
|  | Self_Past_vs_Pat_Dia_Test | 1.65 | .40 | 7.00 | .07 | 1.31 | .75 | .15 | .80 | 6105.5 | .75 | 6269 | .37 | 4948 | .40 | 3371.5 | .87 |
| **For SVM** | Hold-Out Test | 1.49 | .48 | 1.13 | .48 | 2.02 | .48 | 1.05 | .48 | 6956.5 | .87 | 5183 | .75 | 6166 | .48 | 4493.5 | .14 |
|  | HO_Cont_vs_Self_Pat_Dia_Test | 7.32 | .054 | .16 | .79 | .06 | .97 | .97 | .79 | 6766 | .79 | 6136 | .79 | 5651.5 | .79 | 2459.5 | .79 |
|  | HO_Cont_vs_Self_Past_Dia_Test | 1.96 | .40 | 3.62 | .23 | 1.11 | .63 | 5.05 | .20 | 4194 | .63 | 4398.5 | .40 | 4004.5 | .63 | 2278.5 | .40 |
|  | Self_Past_vs_Pat_Dia_Test | 1.23 | .52 | 6.07 | .11 | .83 | .75 | 2.61 | .42 | 5862 | .53 | 6275 | .53 | 4632.5 | .53 | 3355 | .91 |
| *NOTES. * Indicates significance after Benjamini-Hochberg correction.*  *1: In-group comparison of the demographic variables of the groups forming the test sets.*  *2: A comparison to assess the confounding effect of the demographic variables of the participants in the test set on word count* | | | | | | | | | | | | | | | | | |

**Table S17.** Shows multiple comparisons of whether the self-reported pathology diagnosis and past diagnosis groups created for Study 2 differ from the Hold-out test set.

|  | | **Hold-Out Test Set** | | | | | | | | | | | | | | |
| --- | --- | --- | --- | --- | --- | --- | --- | --- | --- | --- | --- | --- | --- | --- | --- | --- |
|  |  | Gender | | Education Level | | | Income Level | | Age | | Word Count | | Panas_Negative | | Panas_Positive | |
|  |  | *χ²* | *p* | | *χ²* | *p* | *χ²* | *p* | *χ²* | *p* | *U* | *p* | *U* | *p* | *U* | *p* |
| **For BERT** | Self_Past_vs_Pat_Dia_Test | .30 | .69 | | .20 | .69 | 12.82 | .09 | .16 | .69 | 25431 | .24 | 26767.5 | .69 | 30895.5 | .14 |
| **For SVM** | Self_Past_vs_Pat_Dia_Test | 2.48 | .40 | | 1.15 | .40 | 4.45 | .44 | .09 | .76 | 25603 | .40 | 29112 | .40 | 29765 | .40 |
| *NOT.* *Benjamini-Hochberg correction was applied to p values.* | | | | | | | | | | | | | | | |  |

Table S18. It shows comparisons between the self-past diagnostic group (Self_Past_Dia_G) and the hold-out control group(HO_Cont_G) and between the hold-out subclinical group(HO_SubC_G) and the self-psychopathology diagnostic group (Self_Pat_Dia_G) in various aspects.

|  | | I-Talk_O_Count | | I-Talk_G_Count | | Depression Word Count | | Anxiety Word Count | | BSI SCORE | |
| --- | --- | --- | --- | --- | --- | --- | --- | --- | --- | --- | --- |
|  |  | *U* | *p* | *U* | *p* | *U* | *p* | *U* | *p* | *U* | *p* |
| **For BERT** | HO_SubC_G vs Self_Pat_Dia_G | 7762.5 | .02* | 8719.5 | .32 | 9179.5 | .47 | 8221.5 | .12 | 8384 | .17 |
|  | HO_**Cont**_G vs Self_Past_Dia_G | 4747 | .88 | 4770.5 | .88 | 3617.5 | .02* | 3505 | .01* | 3996.5 | .22 |
| **For SVM** | HO_SubC_G vs Self_Pat_Dia_G | 7385.5 | .005* | 8507.5 | .25 | 9169.5 | .59 | 7683.5 | .02* | 7901 | .05 |
|  | HO_**Cont**_G vs Self_Past_Dia_G | 4466.5 | .67 | 4477.5 | .67 | 4048.5 | .50 | 3942 | .34 | 4501.5 | .72 |
| *NOT.* * It is significant after Benjamini-Hochberg correction. (*p* < .05). | | | | | | | | | | | |

References

Akkoyun, F. (2014). *Projektif teknikler* (3. Basım). *Yayın No: 1086. Eğitim No: 237*. Nobel.

Benjamini, Y., & Hochberg, Y. (1995). Controlling the False Discovery Rate: A Practical and Powerful Approach to Multiple Testing. *Journal of the Royal Statistical Society: Series B (Methodological)*, *57*(1), 289–300. https://doi.org/10.1111/j.2517-6161.1995.tb02031.x

Chen, C.‑F., & Rothschild, R. (2010). An Application of Hedonic Pricing Analysis to the Case of Hotel Rooms in Taipei. *Tourism Economics*, *16*(3), 685–694. https://doi.org/10.5367/000000010792278310

Dohoo, I. R., Ducrot, C., Fourichon, C., Donald, A., & Hurnik, D. (1997). An overview of techniques for dealing with large numbers of independent variables in epidemiologic studies. *Preventive Veterinary Medicine*, *29*(3), 221–239. https://doi.org/10.1016/S0167-5877(96)01074-4

Gwet, K. L [Kilem Li] (2008). Computing inter-rater reliability and its variance in the presence of high agreement. *The British Journal of Mathematical and Statistical Psychology*, *61*(Pt 1), 29–48. https://doi.org/10.1348/000711006X126600

Jafari, M., & Ansari-Pour, N. (2019). Why, When and How to Adjust Your P Values? *Cell Journal*, *20*(4), 604–607. https://doi.org/10.22074/cellj.2019.5992

Koç, G., Çolak, B., Tatlı, S. Z., İlhan, R. S., & Oncu, B. (2021). Beier Sentence Completion Test Profiles of Adolescents and Emerging Adults With Internalizing and Externalizing Disorders. *Adolescent Psychiatry*, *11*(4), 240–259. https://doi.org/10.2174/2210676611666211124144004

Lin, F.‑J. (2008). Solving Multicollinearity in the Process of Fitting Regression Model Using the Nested Estimate Procedure. *Quality & Quantity: International Journal of Methodology*, *42*(3), 417–426. https://ideas.repec.org/a/spr/qualqt/v42y2008i3p417-426.html

Lundberg, S. M., & Lee, S.‑I. (2017). A Unified Approach to Interpreting Model Predictions. *Advances in Neural Information Processing Systems*, *30*.

O’brien, R. M. (2007). A Caution Regarding Rules of Thumb for Variance Inflation Factors. *Quality & Quantity*, *41*(5), 673–690. https://doi.org/10.1007/s11135-006-9018-6

Razali, M. N., & Wah, Y. B. (2011). *Power Comparisons of Shapiro-Wilk, Kolmogorov-Smirnov, Lilliefors and Anderson-Darling Tests* (Vol. 2). https://www.researchgate.net/publication/267205556_Power_Comparisons_of_Shapiro-Wilk_Kolmogorov-Smirnov_Lilliefors_and_Anderson-Darling_Tests

Sullivan, G. M., & Feinn, R. (2012). Using Effect Size-or Why the P Value Is Not Enough. *Journal of Graduate Medical Education*, *4*(3), 279–282. https://doi.org/10.4300/JGME-D-12-00156.1

Wongpakaran, N., Wongpakaran, T., Wedding, D., & Gwet, K. L [Kilem L.] (2013). A comparison of Cohen's Kappa and Gwet's AC1 when calculating inter-rater reliability coefficients: A study conducted with personality disorder samples. *BMC Medical Research Methodology*, *13*(1), 61. https://doi.org/10.1186/1471-2288-13-61

Zec, S., Soriani, N., Comoretto, R., & Baldi, I. (2017). High Agreement and High Prevalence: The Paradox of Cohen's Kappa. *The Open Nursing Journal*, *11*, 211–218. https://doi.org/10.2174/1874434601711010211

1. This study is derived from Erkan Eyrikaya's master's thesis in clinical psychology. [↑](#footnote-ref-1)
2. https://huggingface.co/dbmdz/bert-base-turkish-128k-cased [↑](#footnote-ref-2)
3. In the model explanations, SHAP values for words are context-dependent. However, the model output itself consists of individual words. Therefore, the English translations were made based on the direct meanings of the words, without consideration for specific grammatical categories such as adjectives or adverbs. Additionally, due to subword tokenization, words not included in the model's vocabulary are divided into subword units (for this reason, words that are considered to be split are italicized in the table). As a result, some words may be interpreted as meaningless or convey a different meaning from their original form. This issue represents an existential limitation of explainable artificial intelligence (XAI) in the field of NLP rather than a limitation specific to this study. [↑](#footnote-ref-3)
